# Fluctuations in arousal reflect latent state transitions that facilitate behavioural optimization

**DOI:** 10.1038/s41562-026-02533-1

**Published:** 2026-08-05

**Authors:** Tiantian Li, Harrison Marble, Timothy Chen, Niloufar Razmi, Matthew R. Nassar

**Affiliations:** 1 Department of Neuroscience, Brown University, Providence, RI USA; 2 Robert J. & Nancy D. Carney Institute for Brain Science, Brown University, Providence, RI USA

**Keywords:** Cognitive control, Human behaviour

## Abstract

People are often faced with surprising events that defy expectations. Such events elicit transient activity in the locus coeruleus/norepinephrine system and elevation of peripheral arousal markers including pupil dilation and a late positive peak in stimulus evoked EEG response, the P300, but the function of these signals remains unclear. We propose that they reflect latent state transitions that dynamically control the mental context governing learning and perception. We tested and confirmed five preregistered predictions of this theory using EEG and pupil measurements collected from people performing a colour prediction and reproduction task. Task-induced latent state transitions elicited pupil dilation and amplified event-related potentials including the P3a component of the P300. These EEG and pupil measures related to behavioural signatures of latent state updating, including reduced bias and bidirectional adjustment of learning, both across trials and across individuals. Our findings support the theory that locus coeruleus/norepinephrine-linked arousal systems optimize behaviour by signalling environmental transitions to facilitate rapid adjustments of mental context.

## Abstract (web summary)

Li, Marble et al. examine how moment-to-moment fluctuations in arousal optimize behaviour by signalling environmental transitions and facilitate rapid adjustments of mental context.

## Main

Learning in a complex and dynamic world is difficult and inevitably involves violations of our expectations. For example, when visiting a familiar restaurant, we enter with an expectation about the quality of food, but occasionally these expectations will be challenged by discordant perceptual information (for example, a spicier-than-expected meal). Such surprising events are accompanied by transient arousal responses evident in peripheral markers such as pupil dilation^1–4^. While previous work has made great strides in uncovering the biological bases of transient arousal responses^2,5–11^, their function and contributions to behaviour remain issues of ongoing scientific debate.

Transient fluctuations in arousal are mediated in part by the locus coeruleus/norepinephrine (LC/NE) system. The LC/NE system in humans is composed of fewer than 100,000 NE-releasing neurons with cell bodies located in the LC that project broadly, providing the majority of NE modulation to the brain^9,12,13^. The transient release of NE leads to systematic changes in cortical processing^14–16^, large-scale electrophysiological signatures reminiscent of the P300 orienting response^9–11^ and peripheral markers of arousal such as pupil dilation^1,17–19^. These changes in arousal are accompanied by altered behavioural performance in domains such as learning and perception^2,6,10,20–25^. Despite the frequency with which such fluctuations in arousal occur during everyday life, and the consequences that these fluctuations have on neural processing and behaviour, the exact function that they serve remains unknown.

We recently proposed a theory for how arousal systems are modulated to optimize behaviour across domains^8,26,27^. Our theory posits that the transient activation of the LC/NE system signals the need to update active representations of mental context, which allows the brain to avoid interference that would be caused by combining information across distinct contexts. This theory predicts that fluctuations in LC/NE, and their peripheral arousal signatures, should relate to the optimization of dynamics through which learning^10,25,28–31^ and perceptual bias^20,23,24^ are adjusted in complex environments, as we describe in more detail below.

Our framework builds on theoretical and empirical research suggesting that both learning and perceptual inference are improved through the maintenance of explicit representations of the latent causal process giving rise to observable data, which we refer to here as latent states^32–34^. In complex environments, effective learning and perception requires updating such latent states in real time, in order to effectively attribute learning to the most relevant context and to regularize perception in a context-appropriate manner^31,33,35^. In the above-mentioned example of visiting a familiar restaurant, if we discover that the normal chef is out sick (that is, a different latent state), it might affect how we perceive the food quality (that is, perception), and it also might affect the degree to which we adjust our future expectations about the restaurant according to our current experience (that is, learning). The timing of such latent state transitions is critical for both optimal learning and perception. If outdated latent states linger, then perception will be biased towards irrelevant expectations (for example, when we enter the restaurant thinking we have the normal chef, our gustatory experience will be biased towards the one we expected, rather than accurately reflecting the food in front of us), and credit will be incorrectly assigned for learning (for example, the wrong chef will be blamed for a bad meal). People undergo dynamic adjustments of both learning and bias, consistent with the idea that they are optimizing these processes according to perceived latent state transitions^23,35^. The need for latent state updating is often signalled by an external event that is surprising when viewed through the lens of the active latent state representation (for example, the normal chef makes delicious soup, but today’s soup tastes terrible).

The LC/NE system is well positioned to signal the need for latent state updating. The LC projects broadly throughout the brain and responds transiently to surprising events^13,36^. While the effects of such neuromodulation are varied across tasks and systems, a confluence of data suggests that it may facilitate a rapid reorganization of cortical firing patterns, which has been referred to as a “network reset”^14^. We postulate that the functional role of this network reset is to facilitate a transition to a new active latent state (for example, realizing that this food was not made by the regular chef), thereby promoting an immediate disengagement from expectations stored in the associations linked to the previously active state^8,26,27^. In doing so, such a mechanism would reduce biases to perceive new data in accordance with recent expectations, thereby optimizing the dynamics of perception, and ensure that credit for the change (for example, a bad meal) is attributed to the correct source (for example, the responsible chef), thereby optimizing the dynamics of learning.

While the latent state transition theory (LSTT) accounts for a wealth of data linking arousal signals to surprise^1,37–41^, prediction errors (PEs)^4,42–45^, and behaviours including learning^2,6,29,46–48^ and perceptual biases^20,21,23,24,49,50^, it also makes a number of novel predictions that have yet to be tested. Related to learning, the LSTT was developed to explain why electroencephalography (EEG)-derived signatures of arousal, such as the P300, correspond to increased learning after changepoint events but decreased learning after oddball events^31^. But it further predicts that these bidirectional learning relationships should also hold for other peripheral measures of arousal such as pupil dilation, which has yet to be tested. Related to bias, the LSTT accounts for findings that pupil dilations correspond to reduced perceptual bias in changing environments^21,23,24^. But it further predicts that this relationship should hold across different statistical environments (changepoints/oddballs) and across different signals that are sensitive to transient changes in arousal, such as the P300. Both of these predictions have yet to be tested. Finally, if arousal impacts both learning and bias by energizing latent state transitions, then individuals who carve time into discrete latent states should show stronger relationships between arousal and both behavioural measures, supporting a single arousal construct. Here we develop an empirical framework that allows us to test these and other key predictions of the LSTT, using EEG and pupillometry-based markers for arousal as indirect proxies for LC/NE activity.

We tested five preregistered predictions of the LSTT by recording EEG and pupil area as proxies for LC-linked arousal systems during the performance of a colour prediction and reproduction task facilitating measurements of both bias and learning^26^ (OSF ID: AHYNJ). Our results confirmed all five predictions with respect to pupil dilation and the P3a component of the P300. Namely, these arousal markers, when locked to stimulus presentation, reflected latent state transitions (1), co-occurred with reduced perceptual biases (2) and related to learning in a context-appropriate bidirectional manner (3). Furthermore, pupil dilations aligned to predictions were consistent with reflecting an internally generated state transition signal necessary to avoid interference from oddball events (4). Finally, consistent with our theory of a common mechanism for the relationships of arousal to learning and bias, we show that individuals with stronger arousal modulation of bias also showed stronger arousal modulation of learning (5). While our results include several conceptual replications of previous work, we note that predictions 2–5 each include completely new empirical observations that were predicted by the LSTT. It is noteworthy that, while our preregistration did not distinguish between different subcomponents of the P300 response, we found that only the early P3a component related to latent state transitions and their theorized downstream effects on behaviour, whereas the later P3b component was not reliably modulated by latent state transitions. Taken together, our results provide strong support for and constraints on the hypothesis that arousal systems optimize behaviour by signalling the need to update active representations reflecting latent states of the world.

## Results

Our results are organized as follows. First, we introduce a prediction and reproduction task capable of measuring trial-to-trial dynamics in learning and perceptual bias. Next, we validate the task, finding in particular that participants used their predictions to improve reproduction accuracy. We then test each of our five preregistered hypotheses concerning the latent states theory of arousal (osf.io/ahynj; H1–H5).

### A combined prediction and reproduction task for testing latent states theory of arousal

**Fig. 1:**
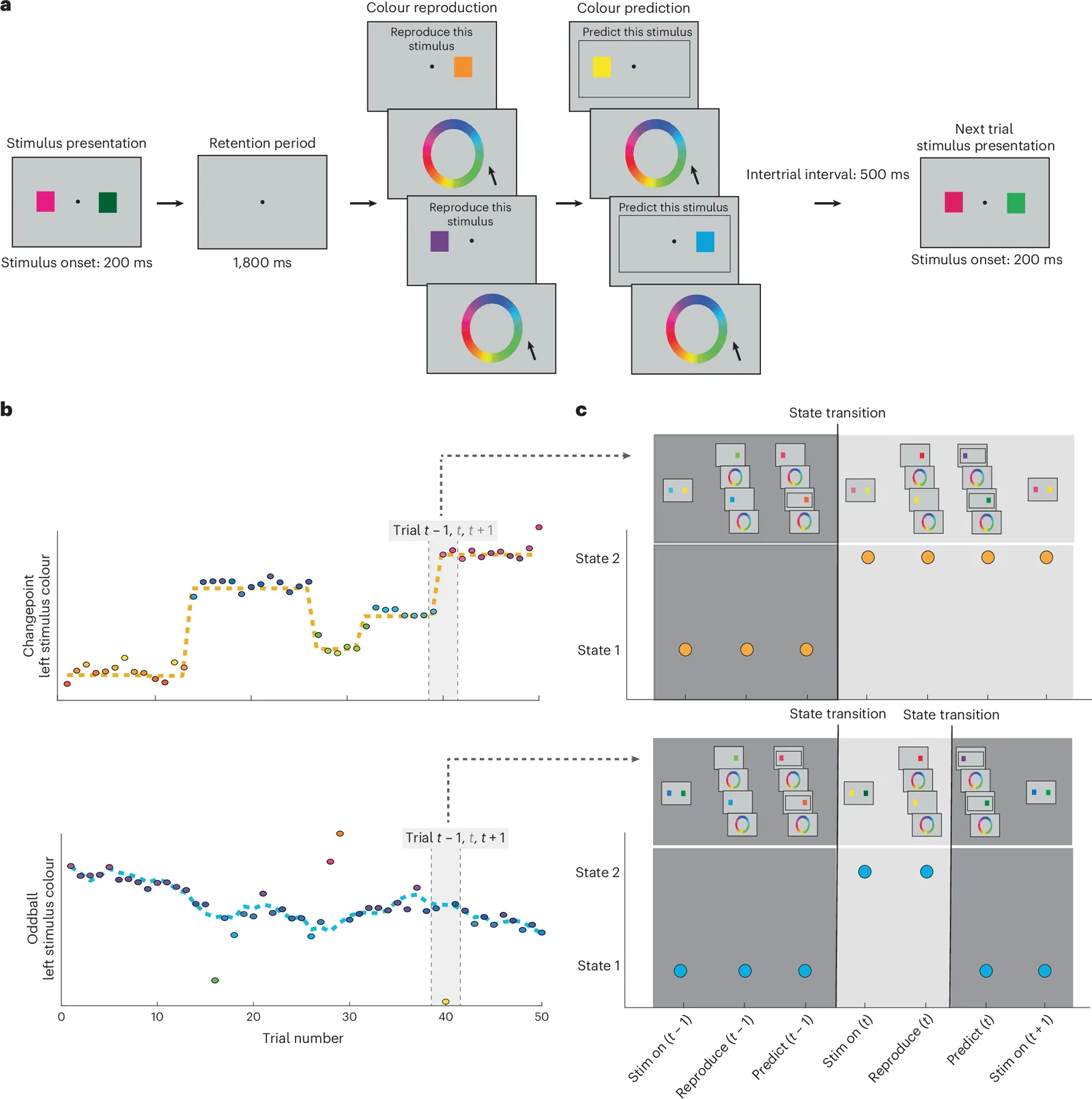
Task schematics. **a**, On each trial, participants went through three main phases: stimulus presentation, colour reproduction and colour prediction. **b**, Examples of how the colours of one stimulus evolved across trials in the two different conditions. As shown by the colours of the circles in this figure, stimuli with close values in degree (*y* axis) were similar in colour. **c**, A detailed look into latent state transition structures across two conditions at each task step before and after a surprising trial. In **c**, both the changepoint and oddball conditions had a latent state transition on trial *t*. In the changepoint condition, the latent state transitioned at stimulus onset on trial *t* and stayed in the newly transitioned state until the next surprise stimulus. In the oddball condition, after transitioning to a new state with a surprising stimulus, the state transitioned back to its previous state after colour reproduction in trial *t* to prepare for the next stimulus at trial *t* + 1. So, the key difference between the two conditions was that changepoints gave rise to persistent changes in latent state, whereas oddballs led to a transient change in latent state and rapid reversion to the previously active latent state.

**Extended Data Fig. 1:**
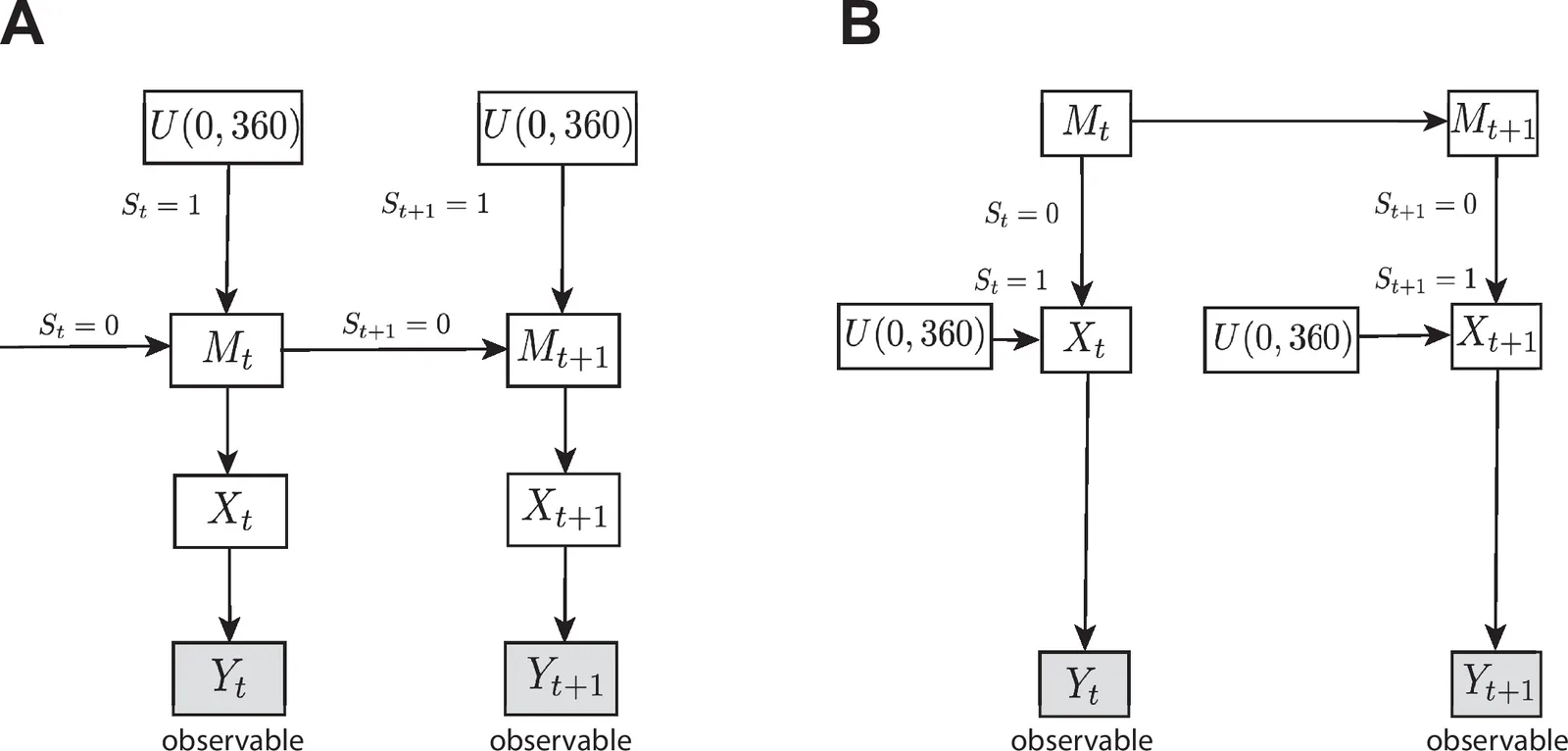
Task generative model creates changepoint and oddball structures. **(a)** Generative model for the changepoint block. Each trial’s presented color, *X*, is generated about the underlying mean, *M*. On non-changepoint trials, *M* remains constant from previous trials. On changepoint trials, *M* is sampled randomly from a uniform distribution about the color space. **(b)** Generative model for the oddball block. On normal trials, presented color, *X*, is generated about an underlying mean, *M*. On oddball trials, *X* is selected from a uniform distribution about the color space. Between each trial, *M* drifts with variance, *σ*.

To test whether arousal systems relate to the optimization of learning and perceptual bias dynamics in line with our latent states theory, we developed a prediction/reproduction task that allowed us to measure both learning and perceptual bias in two different conditions differing in their latent state dynamics. On each trial, participants (1) briefly viewed two coloured patches (200 ms), (2) sequentially reproduced each colour from memory and then (3) sequentially predicted each colour expected on the next trial (Fig. 1a). Participants performed two blocks of the task, each with its own generative structure that enabled them to predict upcoming colours with some degree of reliability (Fig. 1b). In the changepoint condition, colours were generated from a distribution with a mean that changed occasionally (changepoint hazard rate, 0.15; Fig. 1b, top; see also Extended Data Fig. 1a). In the oddball condition, the mean of the colour distribution drifted slightly on each trial, and colours were occasionally sampled uniformly from the entire colour wheel rather than from the current colour distribution (oddball hazard rate, 0.15; Fig. 1b, bottom; see also Extended Data Fig. 1b). Participants completed one task block of 120 trials for each generative condition, with block order counterbalanced across participants and block environmental structure made explicit through instructions. The two stimuli presented on each trial were sampled from independent generative processes doubling the number of latent state transitions experienced within each block. Pupil area and EEG data were recorded continuously as participants performed the task, allowing us to measure fluctuations in arousal, which served as proxies for underlying LC/NE activation. A key advantage of this task was that it allowed us to measure behavioural constructs of interest on each trial (learning and perceptual bias), including those with latent state transitions (changepoints/oddballs; Fig. 1c), which allowed us to test whether and how behaviour changes with transient signatures of arousal (measured through pupil area and EEG).

Participants were generally accurate in their colour reproductions, but individuals varied in the accuracy of their predictions. The mean absolute colour reproduction error across the two prediction blocks was 19.9°, whereas the mean absolute PE across both blocks was 39.2°. Predictions were slightly more accurate in the changepoint block (mean PE, 36.8°) than in the oddball block (mean PE, 41.7°; changepoint–oddball mean difference, 4.9°; *t*_62_ = 3.73; two-sided *P* = 0.0004; Cohen’s *d* = 0.42; 95% confidence interval (CI), 2.2 to 7.3). Prediction accuracy differed considerably across individuals for both the changepoint (standard deviation, 9.8°) and oddball (standard deviation, 11.6°) conditions, such that predictions were more reliable indicators of the upcoming colour in some individuals than in others.

### Participants combined priors with sensory information to improve reproductions

**Extended Data Fig. 2:**
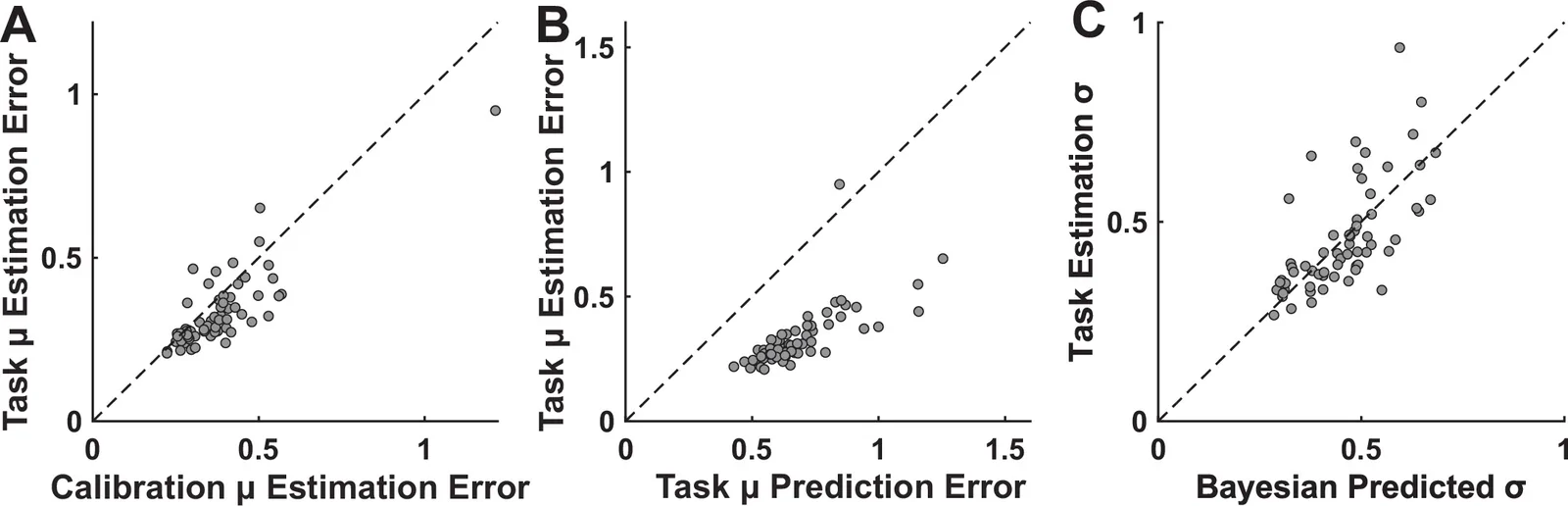
Participants in structured blocks are able to use perceptual bias to improve perception. **(a)** The mean estimation error in the practice phase is significantly higher than in the prediction phase (t(62) = −4.74; two-sided p = 6.45*10^−6^; Cohen’s d =-0.602). **(b)** Mean prediction error is much larger than estimation error standard deviation (t(62) = −24.3; two-sided p = 2.26*10^−33^; Cohen’s d = −3.09) **(c)** Participant estimation error distribution in the prediction phase is not statistically different from the prediction error distribution multiplied by the estimation error distribution in the practice phase (t(62) = −0.95; two-sided p = 0.348; Cohen’s d = −0.120).

In theory, combining prior expectations with internal representations of sensory information can allow for more accurate perceptions, particularly in cases where such priors are reliable predictors of future stimuli^51,52^. In particular, Bayesian inference would stipulate that perceptions reflect a weighted average of prior expectations and internal representations of sensory information:

$${\mathrm{Perception}}=w\times P+\left(1-w\right)\times S$$

where *P* is the prior expectation, *S* is the sensory information and the weights (*w* and 1 − *w*) are proportional to the relative precision associated with each source of information. Note that this implies that perceptions should be biased towards expectations to the degree, *w*, to which those expectations are informative about the observed stimulus. To assess whether participants were able to capitalize on prior expectations to improve their task performance, we first compared reproduction errors observed in our primary task to those from an unstructured colour reproduction control block, in which colours were uniformly sampled and thus prior information was uninformative. We found that reproduction errors were lower in our task than in the unstructured control block (Extended Data Fig. 2a; *t*_62_ = −4.74; two-sided *P* = 6.45 × 10^−6^; Cohen’s *d* = −0.602; 95% CI, 1.60° to 3.84°), suggesting that participants were able to capitalize on predictability in the task to improve the accuracy of their reproductions. Reproduction errors were also much lower than the PEs in the structured blocks (Extended Data Fig. 2b; *t*_62_ = −24.3; two-sided *P* = 2.26 × 10^−33^; Cohen’s *d* = −3.09; 95% CI, 18° to 22°), suggesting that participants used sensory information to at least some degree. On average across trials, reproduction errors during the structured task blocks were similar to those expected from a Bayesian weighted combination of prior information (with reliability determined according to PEs) and sensory likelihood information (with reliability determined according to reproduction errors in the unstructured control block), supporting the idea that participants combined prior and sensory information in a near-optimal manner (Extended Data Fig. 2c; *t*_62_ = −0.95; two-sided *P* = 0.348; Cohen’s *d* = −0.121; 95% CI, −2.06° to 1.20°). Thus, on average across trials, participants combined prior and sensory information to achieve improved reproductions, allowing us to examine how the dynamics of task structure and arousal systems affect this process.

### Pupil and EEG signals reflected state transition probability

While prior expectations could be used to reliably predict upcoming colours most of the time, this was not true when the colour sequence underwent a changepoint or oddball event, which we collectively refer to as state transitions. Our motivating theory posits that arousal systems must be engaged at such state transitions to enable updating of internal latent state representations in order to prevent interference. Stemming from this theory, our first preregistered prediction was that transient markers of arousal, including pupil area and the P300, would be increased in response to surprising stimuli that reflect likely state transitions in both task conditions^26^.

**Fig. 2:**
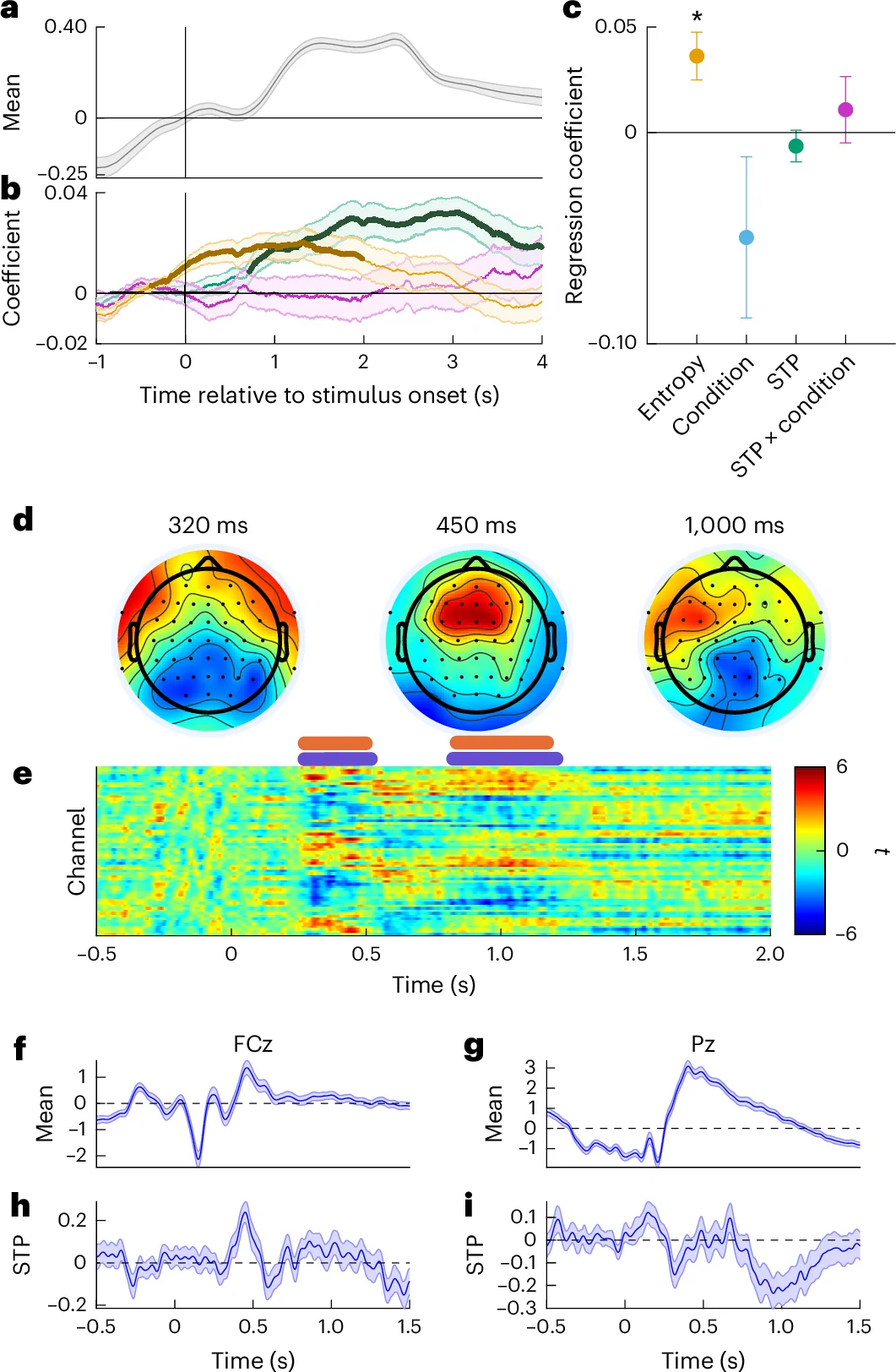
Pupil and EEG correlates of STP. **a**, Mean/s.e.m. baseline regressed pupil area *z* scores plotted as a function of time after stimulus onset. **b**, The lines/shading indicate mean/s.e.m. pupil regression coefficient *z* scores reflecting the relationship between task variables and evoked pupil response plotted as a function of time after stimulus onset for STP (green), entropy (yellow) and STP × condition (purple). The dark points reflect significant temporal clusters after permutation tests controlling for multiple comparisons (STP: cluster mean *t*_59_ = 3.86; two-sided *P* = 0.0004; cluster mass, 12,673; Cohen’s *d* = 0.503; entropy: cluster mean *t*_59_ = 3.38; two-sided *P* = 0.005; cluster mass, 7,982; Cohen’s *d* = 0.439). **c**, The points/lines indicate mean/s.e.m. pupil regression coefficient *z* scores reflecting the relationship between task variables and baseline pupil area. The asterisk reflects a significant *t*-test (entropy *n* = 60; *t*_59_ = 3.0; two-sided *P* = 0.004; Cohen’s *d* = 0.391.) **d**, Scalp topography of *t* statistics summarizing STP coefficients from mass univariate EEG regression is displayed for 320, 450 and 1,000 ms post stimulus presentation. **e**, Heat map displaying STP *t* statistics from the same EEG regression, with channels reflected on the ordinate and time relative to stimulus onset reflected on the abscissa. The horizontal lines indicate cluster time points surviving permutation testing for multiple comparison correction. Orange lines indicate positive clusters; blue lines indicate negative clusters (cluster mean *t*_56_ = 3.34, 2.96, 3.09, 3.06; two-sided *P* = 0.016, 0.010, 0.015, 0.005; cluster masses, 5,349, 5,979, 5,449, 7,647; Cohen’s *d* = 0.447, 0.395. 0.413, 0.409). **f**–**i**, The lines/shading reflect mean/s.e.m. coefficients in microvolts for the baseline-regressed mean (intercept) term (**f**,**g**) and microvolts per standardized unit of STP for the STP term (**h**,**i**) of the EEG regression for channels FCz (**f**,**h**) and Pz (**g**,**i**).

**Extended Data Fig. 3:**
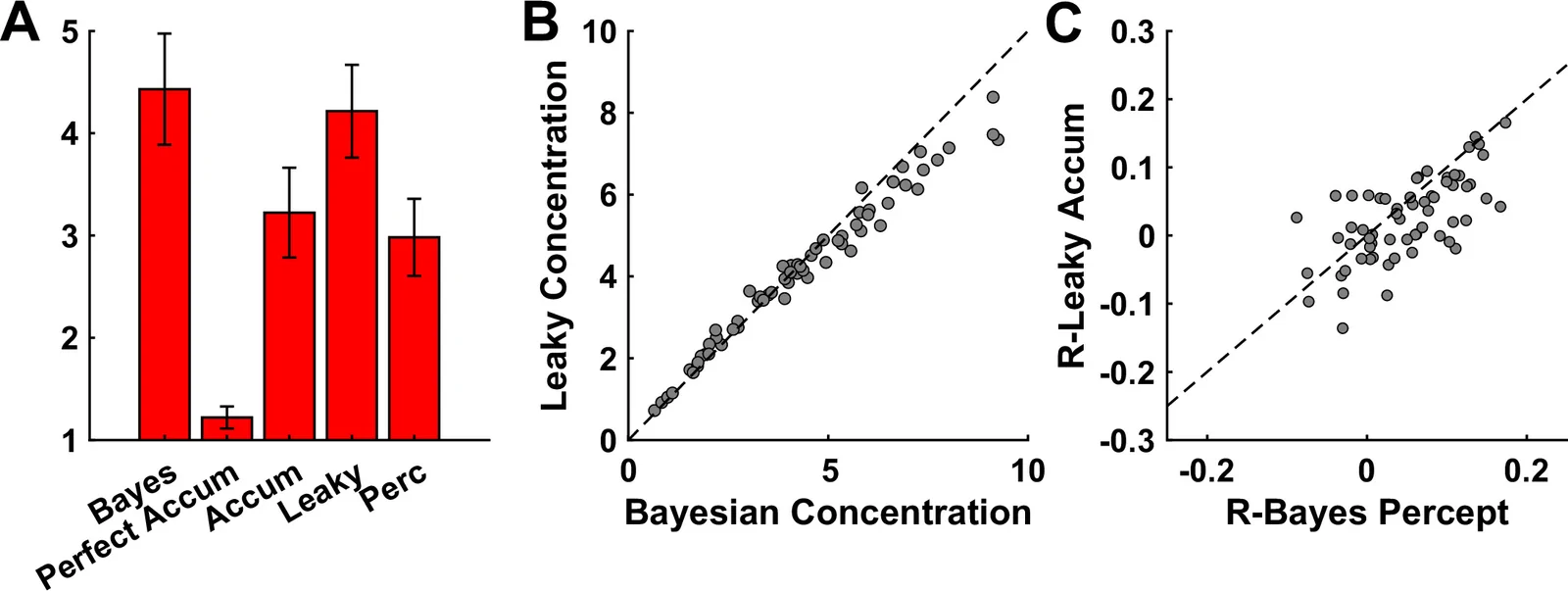
Bayesian model provided a better fit to participant data than simpler heuristic models. **(a)** Model fits (as defined by the concentration of residual errors) for the Bayesian model and simpler heuristic models. We simulated predictions from four heuristic models and compared them to our Bayesian model in terms of their ability to match predictions made by human participants. First, we considered a perfect accumulator, that makes a prediction that reflects the circular mean of all colors observed to this point in the block (Perfect Accum). We also considered a model that resets the accumulation process at state transitions (Accum), such that it predicts the upcoming color according to the mean of all color perceptions since the last changepoint/oddball. We also considered a leaky accumulation model (Leaky), which predicts the upcoming color as a normalized geometrically weighted mean of color perceptions from previous trials. We fit the leak parameter at the group level and found the best performance when past observations were discounted by a decay rate of 0.3. Finally, we also considered a very simple model that makes predictions about the upcoming color completely based on its percept of the last observed color (Perc). For all of our models, percepts from previous trials were set to be the actual color of the presented stimulus plus mean zero Gaussian perceptual noise with standard deviation equal to the mean standard deviation of reproduction errors, across participants, in the unstructured block (31 degrees). We evaluated the degree to which each model could explain participant predictions by running five separate linear regressions that explained the participant predictions in terms of those made by each heuristic model. Since data are circular, this regression included a circular (Von Mises) error distribution, with the concentration (1/variance) of the residual error distribution providing a measure of goodness of fit. While the Bayesian and leaky accumulator models were similar in their ability to fit participant prediction behavior, the Bayesian model performs significantly better than the leaky accumulation model in a statistical comparison (Bayes-Leaky; t(62) = 3.50; two-sided p = 0.001; Cohen’s d = 0.44). **(b)** Bayesian model fits plotted against leaky accumulation model fits for individual subjects. Note that the difference in concentrations is more pronounced in subjects with higher model concentrations, which tend to be those with more accurate predictions. **(c)** We also compared our model’s ability to explain bias in perceptual reports to that of the best heuristic model (leaky accumulator). In this case, we asked to what degree the errors predicted by each model correlated with the reproduction errors produced by participants. On average, the Bayesian perception provided a better correlation with the subject error (Mean r = 0.049) than both the leaky accumulator (Mean r = 0.026; R Bayes Percept-R Leaky; t(62) = 3.60, two-sided p = 0.0003).

We found evidence for such a relationship relating pupil dilations to likely state transitions. For each stimulus, we computed the probability that the stimulus reflected a state transition (state transition probability (STP)) using a Bayesian ideal observer model calibrated to the appropriate task condition (oddball or changepoint), which we found to be a better representation of participant behaviour than simpler heuristic models (Extended Data Fig. 3). We then performed a running linear regression on participant pupil data, using the average STP of the two stimuli as a key explanatory variable. We found that pupils dilated substantially after stimulus presentation (Fig. 2a) and that this dilation was larger for trials with higher STP. This enhanced pupil dilation occurred during a window 1.5–4 seconds after stimulus onset (Fig. 2b, green). Consistent with previous work^2,23^, we also found that baseline pupil size correlated with trial-to-trial entropy, which quantified uncertainty on the belief distribution over possible generative means prior to observing the stimuli (Fig. 2c). Beyond this, we found that entropy had a small effect on the magnitude of pupil dilations immediately after stimulus onset (Fig. 2b, yellow). Pupillometry data thus supported the overarching prediction that arousal measures are elevated at state transitions and the uncertainty resulting from them.

**Extended Data Fig. 4:**
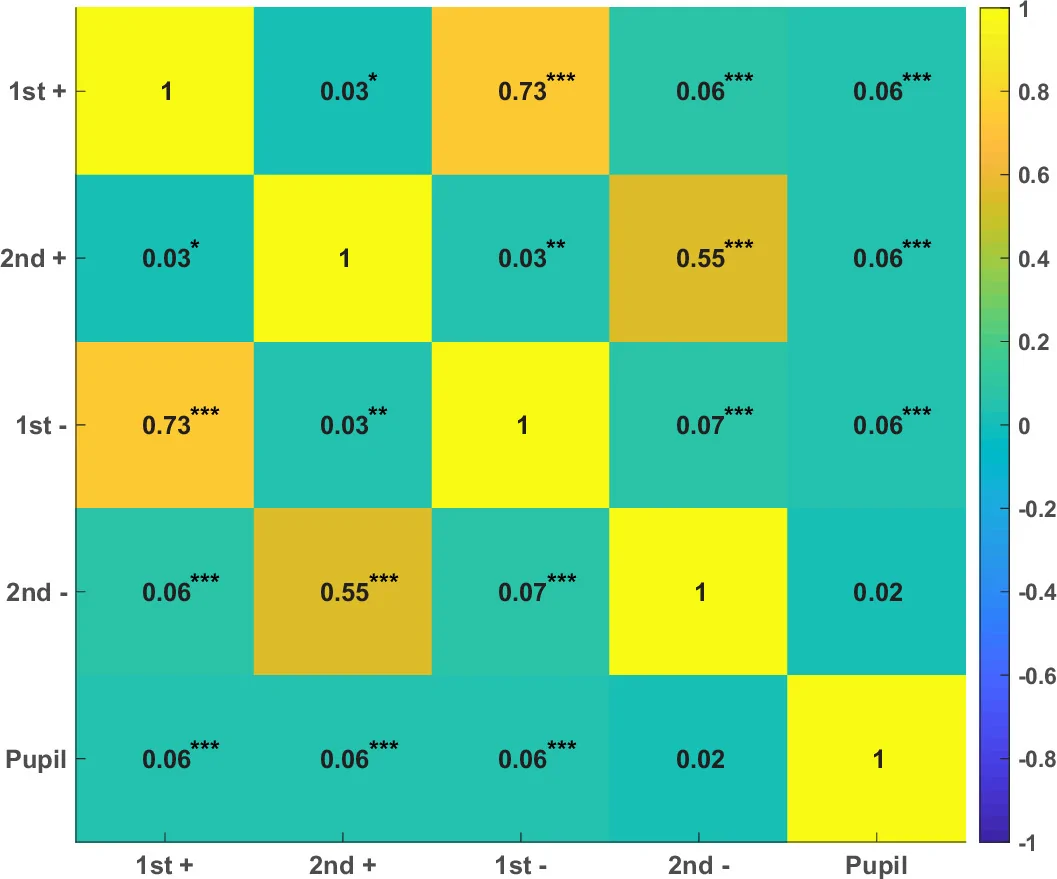
Correlation between individual EEG and pupil signals. Average correlation coefficients across participants between different components of the state transition signal (1^st^ positive P3a-like signal, second frontal positive, 1^st^ parietal negative, second parietal negative, pupil dilation), values marked * indicate p < 0.05, ** indicate p < 0.01, *** indicate p < 0.001.

We next examined whether event-locked P300 responses, which are also thought to reflect a transient arousal and activation of the LC/NE system, were enhanced in response to surprising colours indicative of state transitions. To do so, we performed a running linear regression on the EEG data that included STP as an explanatory variable. We found that, on average, stimulus onset induced P300-like responses at both frontal and parietal electrodes (Fig. 2f,g). To test whether the magnitude of these responses increased at state transitions, we examined the STP coefficients from the regression model and found four spatiotemporally clustered event-related potentials (ERPs) related to STP that survived multiple comparisons correction: a positive frontally localized signal resembling a P3a about 450 ms after stimulus onset, a negative parietal signal around the same time, a second separate negative parietal signal peaking around 900 ms after stimulus onset and a second separate positive frontal signal peaking around 900 ms after stimulus onset (Fig. 2d,e). The two early ERPs and the two late ERPs were both highly correlated with one another (*r*-early = 0.73, *r*-late = 0.55), and all clusters except the late negative cluster were significantly correlated with the pupil dilation associated with STP (Extended Data Fig. 4). Our EEG data thus supported the overarching preregistered prediction that the P300 would reflect STP, but at a finer grain they demonstrated that this was true for only the early frontal P3a (as opposed to the parietal P3b, the later subcomponent of the P300) and also highlighted three additional event-locked signals that reflect STP.

### Perceptual bias was reduced with arousal responses to latent state transitions

#### Participants reduced bias when confronted with surprising stimuli

In stationary environments, people bias perception towards prior expectations, allowing them to achieve more accurate perceptions through Bayesian inference^51,52^. As described above, participants in our study appeared to combine prior knowledge and observed stimuli to improve performance on aggregate across all trials. However, priors become less useful at state transitions, when observed stimuli diverge from expectations. Optimal inference in complex environments requires decreasing bias in response to surprising stimuli if those stimuli are indicative of a change in the underlying state of the environment^23^. A signal that reflects subjective perceptions of state transitions should thus reduce reliance on priors, which could be measured as a reduction of bias towards them.

**Fig. 3:**
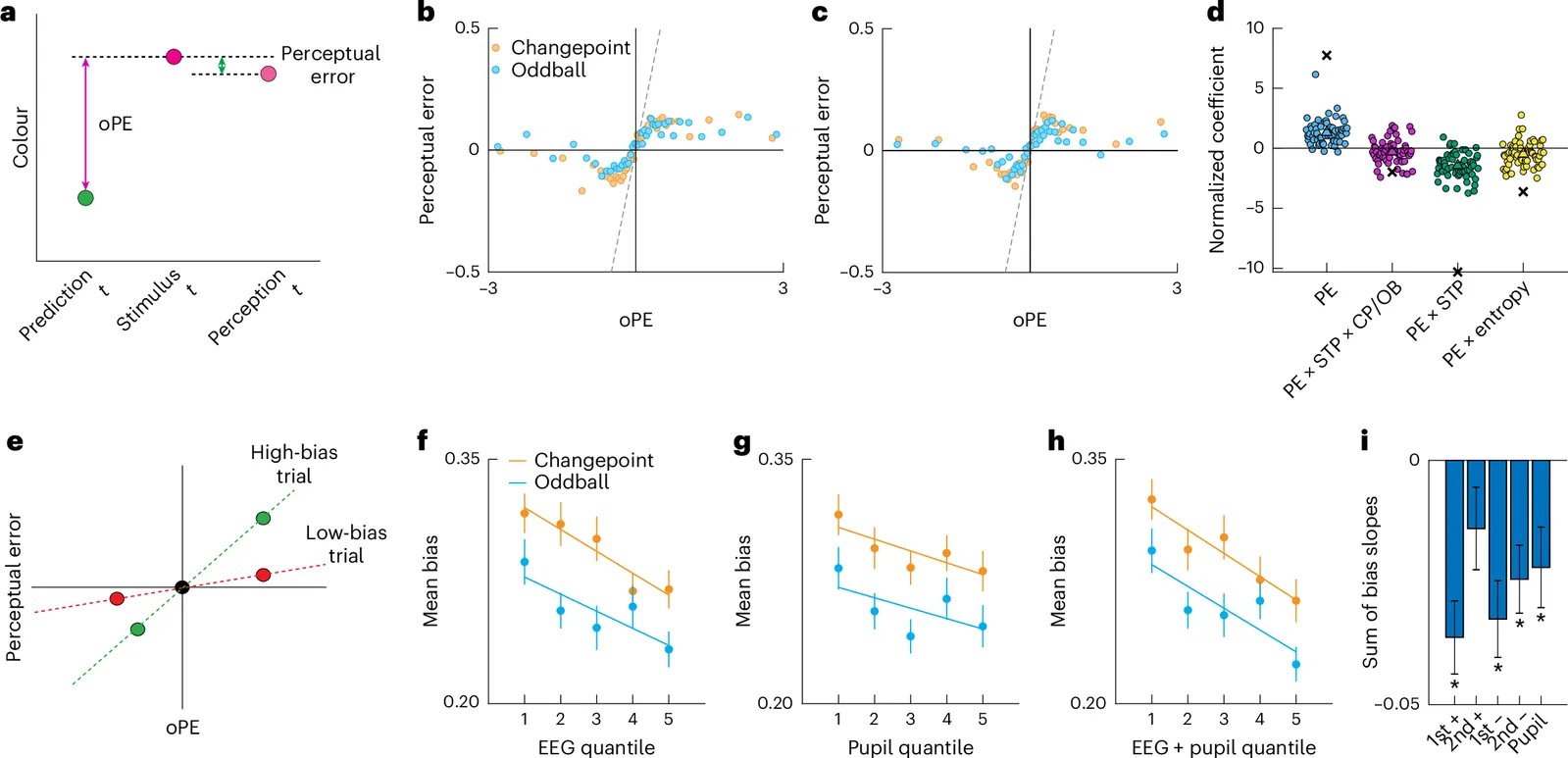
EEG and pupil signals that reflect state transitions correspond to reductions in perceptual bias. **a**, Bias was calculated by dividing perceptual error by oPE. **b**,**c**, Mean perceptual error (ordinate) is plotted for bins of oPE (abscissa) separately for changepoint (orange) and oddball (blue) conditions across all participants (**b**) and in just those participants achieving lower-than-median perceptual errors (**c**). Note that the positive relationship between PEs and perceptual errors was reduced for extreme errors, an effect that is even more pronounced in the highest-performing participants. **d**, Participant coefficients from a circular regression of trial-to-trial perceptual errors (abscissa) are plotted separately for terms in the regression modelling capturing a fixed influence of PEs on perceptual errors, as well as for interaction terms corresponding to dynamic adjustments of bias according to STP (green), STP × condition interaction (purple) or entropy (yellow). The triangle markers and error bars indicate means and 95% CIs. PE coefficients were positive across participants, reflecting a perceptual bias towards predicted colours (*t*_62_ = 10.4; two-sided *P* = 2.95 × 10^−15^; Cohen’s *d* = 1.39; *n* = 63), whereas PE × STP coefficients were negative, reflecting a reduction in perceptual bias for stimuli that reflected likely state transitions (*t*_62_ = −11.5; two-sided *P* = 5.62 × 10^−17^; Cohen’s *d* = −1.46; *n* = 63). CP, changepoint; OB, oddball. Crosses/x’s represent coefficients from the same circular regression using behaviours generated by the ideal observer model. **e**, Schematic depicting how a single-trial measure of bias was calculated for two stimuli presented on a given trial. A line was fit to the origin and both trials’ points on an oPE/perceptual error plane, and its slope provided a single-trial measure of bias. **f**–**h**, Increased strength of STP signals extracted from EEG (changepoint: *t*_56_ = −3.90; two-sided *P* = 2.60 × 10^−4^; Cohen’s *d* = −0.521; *n* = 57; oddball: *t*_56_ = −3.71; two-sided *P* = 4.67 × 10^−4^; Cohen’s *d* = −0.496; *n* = 57) (**f**), pupil (changepoint: *t*_59_ = −2.19; two-sided *P* = 0.033; Cohen’s *d* = −0.285; *n* = 60; oddball: *t*_59_ = −2.06; two-sided *P* = 0.043; Cohen’s *d* = −0.268; *n* = 60) (**g**) and both (changepoint: *t*_55_ = −3.26; two-sided *P* = 0.002; Cohen’s *d* = −0.440; *n* = 56; oddball: *t*_55_ = −4.06; two-sided *P* = 1.56 × 10^−4^; Cohen’s *d* = −0.547; *n* = 56) (**h**) corresponded to decreased bias. The points and error bars indicate the mean and s.e.m. of bias within each quantile across participants. **i**, EEG clusters and pupil signal are differentially related to bias, with the first positive ERP having the strongest relationship. The error bars indicate 95% CIs, and the asterisks indicate *P* < 0.05 (*t*_56, 56, 56, 56, 59_ = −4.84, −1.66, −4.14, −3.51, −2.63; two-sided *P* = 1.10 × 10^−5^, 0.103, 1.20 × 10^−4^, 8.95 × 10^−4^, 0.011; Cohen’s *d* = −0.647, −0.222, −0.553, −0.469, −0.342; *n* = 57, 57, 57, 57, 60).

To test this idea, we first verified that participants in our study reduced their bias appropriately in response to surprising stimuli. Bias in our task can be thought of as the degree to which errors in reproduction match the errors made in prediction (objective PE (oPE); Fig. 3a). Consistent with Bayesian inference in dynamic environments, reproduction errors increased linearly with oPE over a wide range of moderate PEs, but the slope of this relationship decreased dramatically for extreme PEs, indicative of a reduction in bias for the most surprising outcomes (Fig. 3b). This effect was present and comparable in both blocks and was even more pronounced for participants who made the smallest reproduction errors (Fig. 3c). To quantify these effects across participants, we performed a linear regression that predicted reproduction error on the basis of the oPE made by the participant as well as its interaction with STP. PE had a positive effect on reproduction error, suggesting that participants were biased overall, but oPE × STP had a negative effect on reproduction error (Fig. 3d; *t*_62_ = −11.5; two-sided *P* = 5.62 × 10^−17^; Cohen’s *d* = 1.46; 95% CI, −0.046 to −0.032), suggesting that this bias was attenuated at state transitions. Thus, participants biased their reproductions towards their predictions and reduced this bias in response to surprising stimuli reflecting likely state transitions.

#### EEG and pupil signals reflecting state transitions were accompanied by reduced perceptual bias

Our ability to measure trial-to-trial fluctuations in perceptual bias allowed us to test a second preregistered prediction of our latent states theory of arousal: that arousal measures, if they signal the need to update latent states, should also be accompanied by reduced levels of perceptual bias resulting from that latent state update^26^. To test whether EEG- and pupil-derived state transition signals related to perceptual bias, we looked at how state transition signals on individual trials related to average bias on those trials. As stated above, we found that EEG and pupil signals related to STP (Fig. 2b,d,e). To obtain single-trial measures of these signals, we computed the dot product of the signal (EEG/pupil) recorded on a given trial with a template state transition signal (in time for pupil signal, in channels and time for EEG signal) derived from the thresholded *t*-map resulting from the STP regression (Methods). Higher/lower values of this measure on a given trial thus reflected more/less STP-like arousal signalling. Since each trial had one EEG/pupil signal and two measures of bias, we combined the bias measurements into a single value by taking the slope of a line fit to a point on the origin and the two stimuli plotted on a coordinate system containing oPE as the abscissa and the reproduction error as the ordinate (Fig. 3e).

Consistent with our hypothesis that P300 magnitude would correspond to reduced perceptual bias, we found that trials more closely resembling the EEG template for state transitions were accompanied by decreased bias in both the changepoint and oddball blocks (Fig. 3f; changepoint: *t*_56_ = −3.90; two-sided *P* = 2.60 × 10^−4^; Cohen’s *d* = −0.521; 95% CI, −0.012 to −0.004; oddball: *t*_56_ = −3.71; two-sided *P* = 4.67 × 10^−4^; Cohen’s *d* = −0.496; 95% CI, −0.012 to −0.004). However, this signal’s relationship to decreased bias was not solely driven by the frontal P3a signal as predicted but also related to both of the negative parietal EEG-derived STP clusters (Fig. 3i).

**Extended Data Fig. 5:**
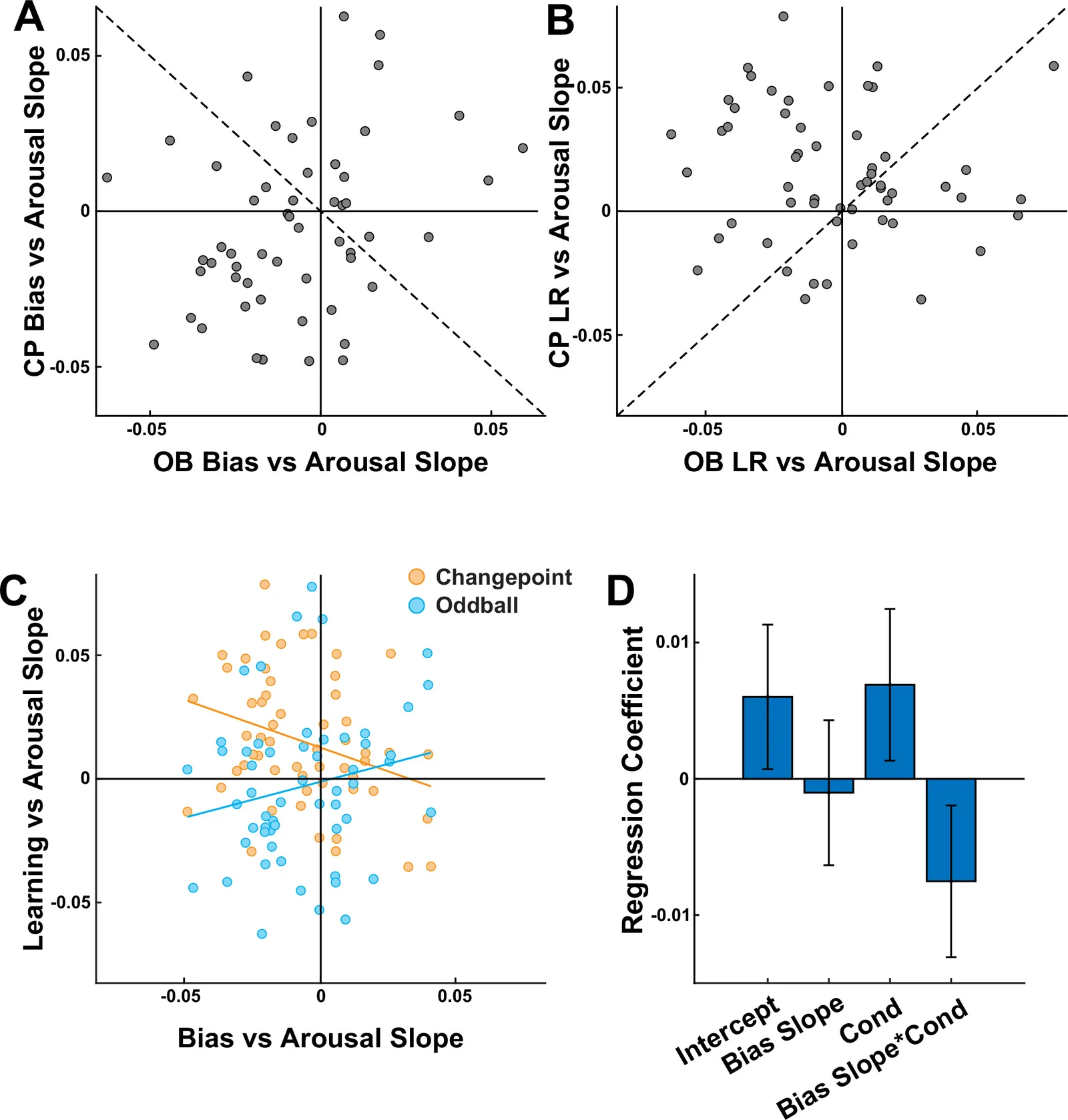
Behavioral relationships to state transition signals are partially maintained even when STP is removed from learning rate, bias, and arousal signals. **(a)** After regressing out STP, changepoint bias/arousal slope is not significant (t(55) = −1.41; two-sided p = 0.163; Cohen’s d = −0.190), OB bias/arousal slope is negative on average (t(55) = −2.49; two-sided p = 0.016; Cohen’s d = −0.336), and the sum of CP and OB bias/arousal slope is negative (t(55) = −2.38; two-sided p = 0.021; Cohen’s d = −0.321). **(b)** After regressing out STP, changepoint learning/arousal slope is positive on average (t(55) = 4.23; two-sided p = 8.85*10^−5^; Cohen’s d = 0.570), OB learning/arousal slope is not different from zero (t(55) = −0.72; two-sided p = 0.475; Cohen’s d = −0.097), and the difference between CP and OB learning/arousal slopes is positive (t(55) = 3.11; two-sided p = 0.003; Cohen’s d = 0.419). **(c)** Individual differences plot on STP-regressed residuals. blue points indicate participants’ slopes in the oddball lock, orange points indicate slopes in the changepoint block. While learning vs arousal slopes were individually calculated within each block, bias vs arousal slopes were calculated across both blocks. **(d)** Regression results against learning slopes. The negative effect of bias slope * condition suggests the relationship between learning/arousal slope and bias/arousal slope is partially maintained even when STP is removed (Bias slope * condition t(108) = −2.67; two-sided p = 0.009; Cohen’s d = −0.257). Error bars indicate 95% confidence interval.

Pupil dilations were also related to reductions in bias. We regressed the trial-by-trial pupil-derived state transition signal against trial average bias and found that the physiological state transition signal was related to decreased average bias across both conditions (Fig. 3g; changepoint: *t*_59_ = −2.19; two-sided *P* = 0.033; Cohen’s *d* = −0.285; 95% CI, −0.021 to −0.001; oddball: *t*_59_ = −2.06; two-sided *P* = 0.043; Cohen’s *d* = −0.268; 95% CI, −0.022 to −0.0004). Thus, in both of our proxies for LC activity, we found that there was a relationship between state-transition-mediated arousal and decreased bias. Combining across pupil and EEG measures revealed even more impressive relationships (Fig. 3h; changepoint: *t*_55_ = −3.26; two-sided *P* = 0.002; Cohen’s *d* = −0.440; 95% CI, −0.020 to −0.005; oddball: *t*_55_ = −4.06; two-sided *P* = 1.56 × 10^−4^; Cohen’s *d* = −0.547; 95% CI, −0.021 to −0.007) that held up even after we controlled for model-based measures of STP (Extended Data Fig. 5a; *t*_55_ = −2.38; two-sided *P* = 0.021; Cohen’s *d* = −0.321; 95% CI, −0.025 to −0.002). The arousal measures thus appeared to provide subjective measures of surprise linked to state transitions and their behavioural consequences in terms of reduced bias.

### Participants bidirectionally modulated learning according to arousal and temporal structure

#### Participants adjusted learning in a condition-appropriate manner

**Fig. 4:**
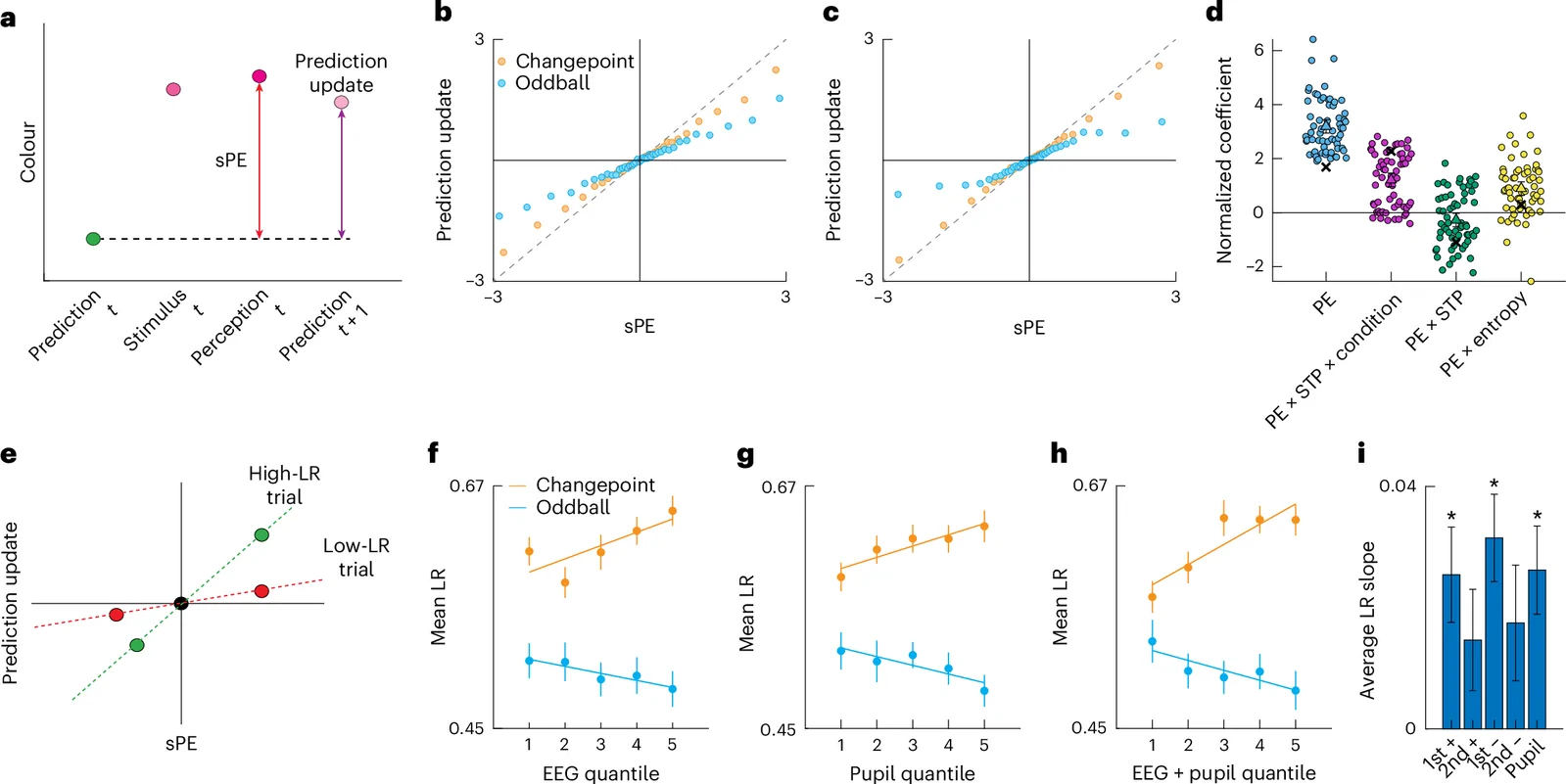
EEG and pupil signals that reflect state transitions are related to bidirectional adjustments in learning rate. **a**, Learning rate was calculated as the degree of prediction update for a given sPE. **b**,**c**, Mean prediction update (ordinate) is plotted for bins of sPE (abscissa) separately for changepoint (orange) and oddball (blue) conditions across all participants (**b**) and for just those participants who achieved lower-than-median average PEs (**c**). Note that updates for extreme PEs diverged for the two conditions, with large PEs promoting increased learning in the changepoint condition and decreased learning in the oddball condition, an effect that is most obvious when looking at the participants who made the most accurate predictions. **d**, Participant coefficients from a circular regression of trial-to-trial prediction updates (abscissa) are plotted separately for terms in the regression model capturing a fixed influence of PEs on updates, as well as for interaction terms corresponding to dynamic adjustments of learning according to STP (green), STP × condition interaction (purple) or entropy (yellow). The triangle markers and error bars indicate means and 95% CIs. PE coefficients were positive across participants (*t*_62_ = 25.2; two-sided *P* = 2.62 × 10^−34^; Cohen’s *d* = 3.20; *n* = 63), reflecting an overall tendency to update colour predictions towards recently observed colours, as were PE × STP × condition coefficients, reflecting the tendency of participants to increase learning in the face of changepoints but decrease it in the face of oddballs (*t*_62_ = 9.57; two-sided *P* = 7.91 × 10^−14^; Cohen’s *d* = 1.22; *n* = 63). Crosses/x’s represent coefficients from the same circular regression using behaviours generated from the ideal observer model. **e**, Example calculation of trial mean learning rate (LR). A line was fit to points corresponding to the prediction update (ordinate) and PE (abscissa) for each of the two stimuli, as well as a third point corresponding to the origin. The slope of this line was taken to reflect the average learning rate for the trial. **f**–**h**, Increased strength of state transition signals from EEG (changepoint: *t*_56_ = 3.08; two-sided *P* = 0.004; Cohen’s *d* = 0.412; *n* = 57; oddball: *t*_56_ = −2.41; two-sided *P* = 0.019; Cohen’s *d* = 0.322; *n* = 57) (**f**), pupil (changepoint: *t*_59_ = 3.06; two-sided *P* = 0.003; Cohen’s *d* = 0.398; *n* = 60; oddball: *t*_59_ = −2.42; two-sided *P* = 0.019; Cohen’s *d* = 0.315; *n* = 60) (**g**) or both measures combined (changepoint: *t*_55_ = 4.89; two-sided *P* = 9.24 × 10^−6^; Cohen’s *d* = 0.659; *n* = 56; oddball: *t*_55_ = −2.19; two-sided *P* = 0.033; Cohen’s *d* = 0.295; *n* = 56) (**h**) was conditionally related to single-trial learning. Increased state transition signalling reflected more learning in the changepoint condition and less learning in the oddball condition. The points and error bars indicate the mean and s.e.m. of learning rate within each quantile across participants. **i**, EEG clusters and pupil dilation were differentially related to learning, with the first negative ERP having the strongest relationship. The error bars indicate 95% CIs (*t*_56, 56, 56, 56, 59_ = 3.25, 1.76, 5.04, 1.83, 3.59; two-sided *P* = 0.002, 0.085, 5.30 × 10^−5^, 0.072, 6.74 × 10^−4^; Cohen’s *d* = 0.434, 0.235, 0.673, 0.245, 0.467; *n* = 57, 57, 57, 57, 60).

Latent state transitions also require separating information in the service of learning in order to minimize the negative impacts of interference. A key advantage of our task is that it allows us to compare predictions made before and after observing a stimulus to derive a single-trial behavioural index of learning. In particular, learning in this task could be thought of as the degree to which predictions were updated (prediction update; the difference between the prediction on trial *t* − 1 and the prediction on trial *t*) for a given subjective PE (sPE) (the difference between the colour predicted on trial *t* − 1 and the colour perceived on trial *t*). The slope of the relationship between prediction updates and PEs thus provided a single-trial measure of learning rate^2^ (Fig. 4a).

Consistent with normative prescriptions for learning^31^, participants updated predictions in response to sPE and modulated their learning rates for large PEs differently across the two environmental conditions. For moderate PEs, participants increased their updates roughly in proportion to sPE in both the changepoint and oddball conditions (Fig. 4b). However, for extreme PEs, participants updated their predictions more in the changepoint than in the oddball block (Fig. 4b). This effect was even more pronounced in the participants that made the most accurate predictions (Fig. 4c). To quantify this effect for individual participants, we performed a linear regression that explained prediction update in terms of sPE and several interaction terms that modulated the effect of sPE (Methods). Consistent with the binned data (Fig. 4b,c) we found that sPE coefficients were positive, capturing the overall positive relationship between sPE and updating (Fig. 4d, blue). In addition, we found that the sPE × STP × condition coefficient was positive, suggesting that the slope of the relationship between updating and sPE was increased on changepoint trials and decreased on oddball trials (Fig. 4d, purple; *t*_62_ = 9.57; two-sided *P* = 7.91 × 10^−14^; Cohen’s *d* = 1.22; 95% CI, 0.085 to 0.130). There was also a small but statistically significant negative effect of sPE × STP on prediction update, suggesting that participants reduced their learning more on oddball trials than they increased it on changepoint trials (Fig. 4d, green), as well as a positive sPE × entropy effect, suggesting that participants learned more during periods of uncertainty (Fig. 4d, yellow). The Bayesian ideal observer model showed the same pattern of coefficients (Fig. 4d, x markers), supporting the idea that participants adjusted their learning in a normative manner.

#### Transient arousal signatures reflected bidirectional adjustments of learning

Our motivating theory proposes that arousal systems are transiently activated to signal the need for latent state transitions, and that such latent state transitions in turn produce contextual effects on learning rate—namely, increased learning from changepoints and decreased learning from oddballs^27^. This leads to our third preregistered prediction: that arousal measures should relate contextually to learning behaviour, corresponding to increased learning in response to changepoints, but decreased learning in response to oddballs^26^. To test this prediction, we extracted single-trial measures of our arousal signatures as described above. We extracted a single-trial measure of learning rate by combining learning rates corresponding to the two stimuli shown on a given trial into a single value corresponding to the slope of prediction updates relative to the sPE (Fig. 4e). We then regressed our arousal measures (EEG/pupil) onto trial average learning rates.

**Extended Data Fig. 6:**
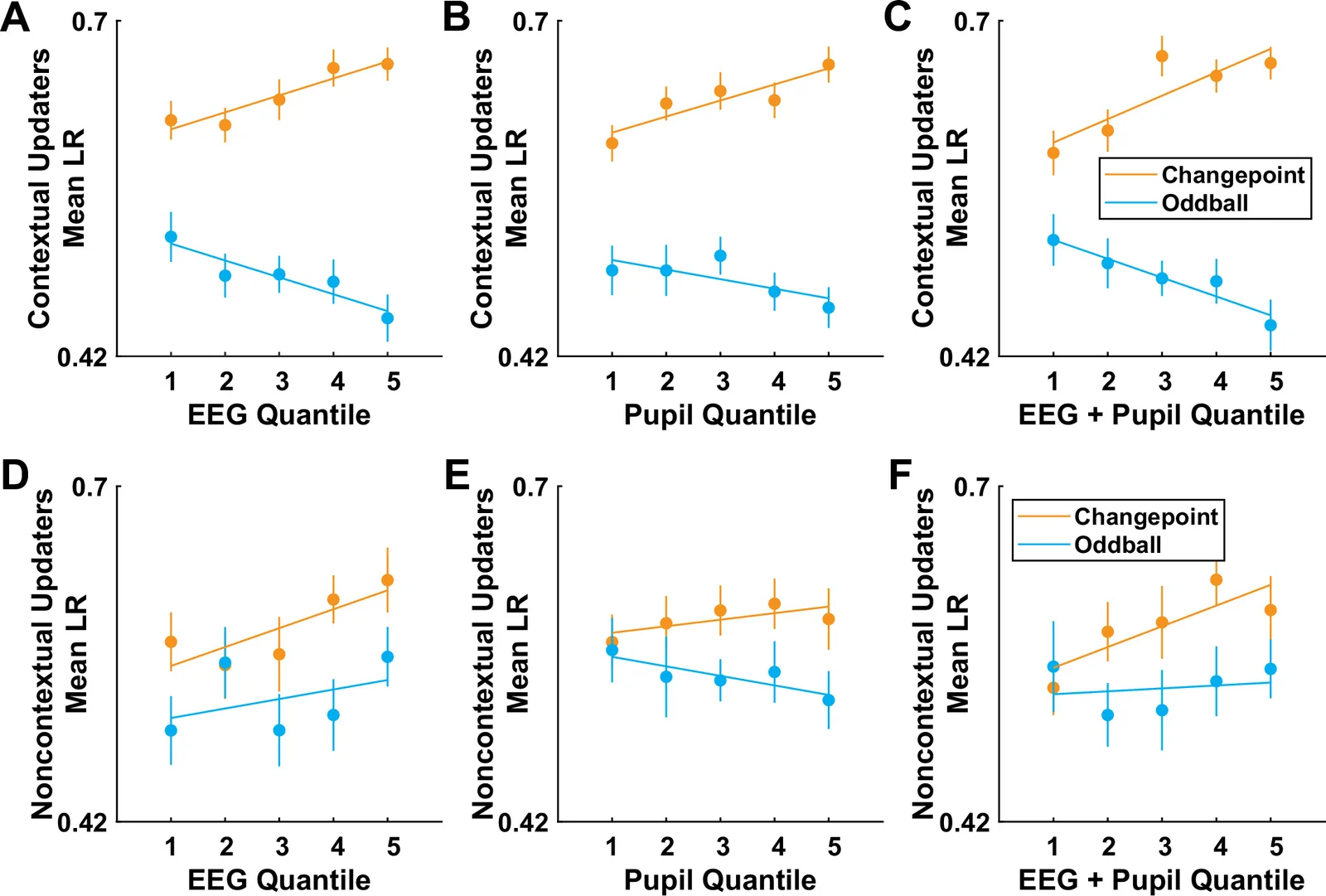
Participants who modulated behavior according to condition had stronger conditional arousal/learning relationships. **(a-c)** For those participants with larger PE*STP*Cond terms as determined by density-based clustering using the MATLAB function dbscan in the behavioral learning regression, mean learning rate (ordinate) is plotted as a function of EEG (left), pupil (middle) and combined (right) quantile (abscissa) separately for changepoint (orange) and oddball (blue) conditions. Note that these participants have strongly diverging learning/arousal slopes across the changepoint and oddball blocks in **(a)** EEG (t(34) = 4.32, two-sided p = 0.0001, Cohen’s d = 0.73), **(b)** pupil (t(35) = 3.37, two-sided p = 0.002, Cohen’s d = 0.56), and **(c)** combined pupil and EEG data (t(34) = 3.95, two-sided p = 0.0002, Cohen’s d = 0.67). **(d-f)** Participants with lower PE*STP*Cond terms in the behavioral regression tend to have more similar learning/arousal slopes across the changepoint and oddball blocks in **(d)** EEG (t(21) = 0.91, two-sided p = 0.371, Cohen’s d = 0.19), **(e)** pupil (t(23) = 1.50, two-sided p = 0.147, Cohen’s d = 0.31), and **(f)** combined pupil and EEG data (t(20) = 1.81, two-sided p = 0.076, Cohen’s d = 0.39).

We found that both physiological markers for state transitions related contextually to learning behaviour in the manner we predicted. Specifically, single-trial ERPs that best matched the state transition template (including larger P300 responses) co-occurred with larger learning rates in the changepoint condition, but lower learning rates in the oddball condition (Fig. 4f; changepoint: *t*_56_ = 3.08; two-sided *P* = 0.004; Cohen’s *d* = 0.412; 95% CI, 0.003 to 0.013; oddball: *t*_56_ = −2.41; two-sided *P* = 0.019; Cohen’s *d* = 0.322; 95% CI, −0.009 to −0.0004). Similarly, the state-transition-related pupil dilation was positively related to learning in the changepoint block and negatively related to learning in the oddball block (Fig. 4g; changepoint: *t*_59_ = 3.06; two-sided *P* = 0.003; Cohen’s *d* = 0.398; 95% CI, 0.005 to 0.025; oddball: *t*_59_ = −2.42; two-sided *P* = 0.019; Cohen’s *d* = 0.315; 95% CI, −0.020 to −0.002). Combining the pupil- and EEG-derived state transition signals together yielded even stronger relationships to learning behaviour (Fig. 4h; changepoint: *t*_55_ = 4.89; two-sided *P* = 9.24 × 10^−6^; Cohen’s *d* = 0.659; 95% CI, 0.011 to 0.026; oddball: *t*_55_ = −2.19; two-sided *P* = 0.033; Cohen’s *d* = 0.295; 95% CI, −0.019 to −0.001) that capitalized on similar relationships across all physiological STP signals (Fig. 4i). These relationships were primarily driven by the participants who modulated learning behaviour most dramatically across the conditions as measured by sPE × STP × condition coefficients in the behavioural regression model (Extended Data Fig. 6). The combined physiological signal was bidirectionally and contextually related to learning even after we statistically controlled for objective STP, suggesting that it provided a subjective measure of STP that was closely linked to adjustments of learning behaviour (Extended Data Fig. 5b; *t*_55_ = 3.11; two-sided *P* = 0.003; Cohen’s *d* = 0.419; 95% CI, 0.006 to 0.030).

### Pupil dilations reflected an internally generated latent state transition

**Fig. 5:**
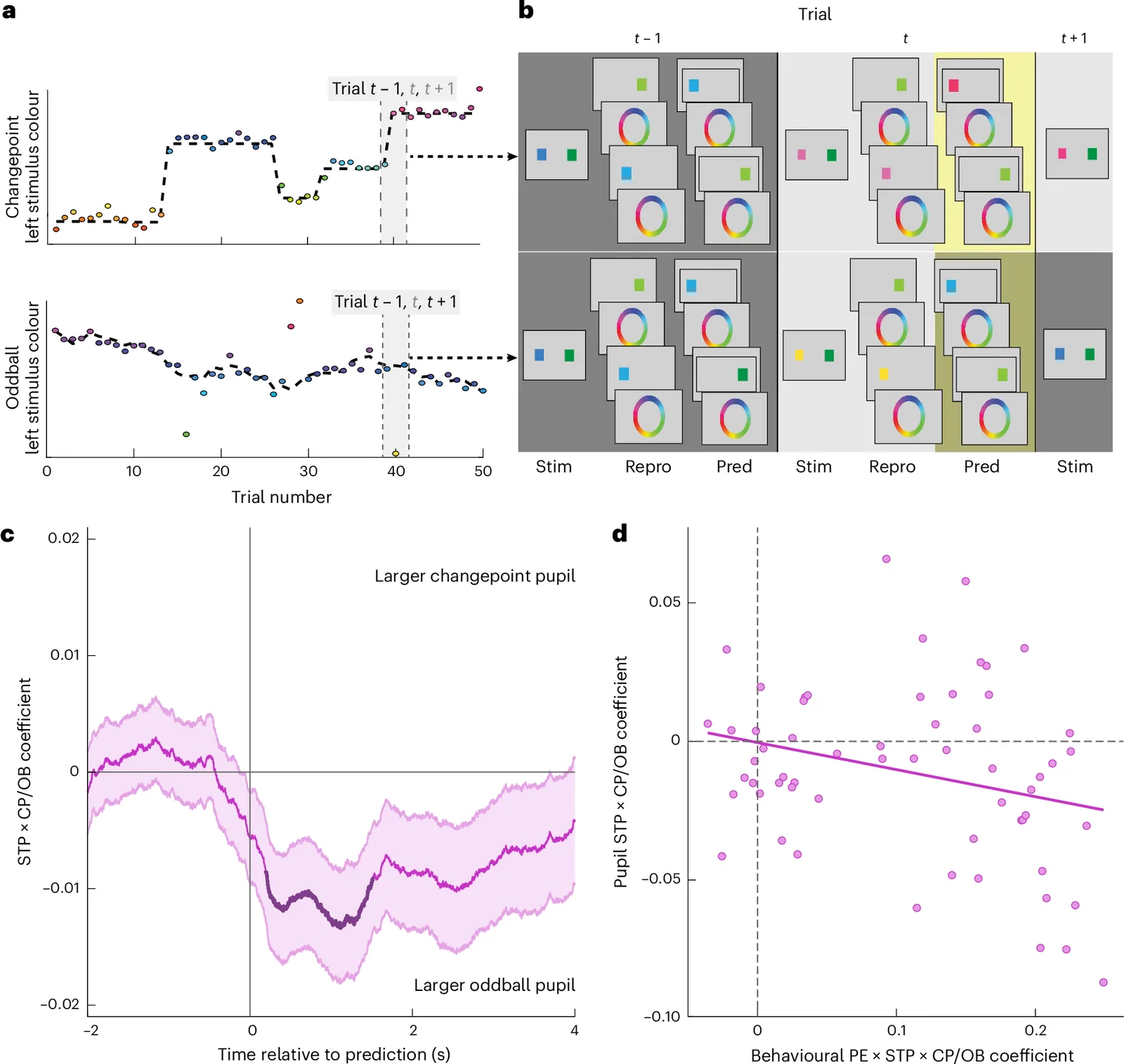
Delayed pupil dilations on oddball trials are consistent with reflecting an internally generated latent state transition. **a**, Trial sequence containing a state transition in both the changepoint (top) and oddball (bottom) conditions. **b**, Task sequence with appropriate reproductions and predictions for the trials before, during and after a state transition trial, showcasing that changepoints require one state transition while oddballs require two, with the second transition reflecting a return to the ‘normal’ state (the background colour reflects the state). The yellow highlighting reflects the time period investigated in **c** and **d**. **c**, Mean/s.e.m. STP × condition coefficient *z* scores from a running linear regression of pupil size locked to the subsequent prediction phase of each trial. The dark points reflect significant clusters after permutation testing for multiple comparisons correction (STP × CP/OB cluster forming threshold, 0.025; cluster mean *t*_59_ = −2.48; two-sided *P* = 0.039; cluster mass, 3,600; Cohen’s *d* = −0.323). Decreased coefficients at the time of the subsequent prediction suggest a second pupil dilation that occurred during the prediction phase after an oddball event. This supports the idea that pupil dilation is related to both internally generated and externally presented state transitions. **d**, Correlation between participants’ behavioural PE × STP × CP/OB coefficient (*z*-scored) from behavioural regression and participants’ STP × CP/OB pupil regression coefficient during the time window of significance (*z*-scored). Those with stronger oddball effects on pupil dilation also tended to have greater learning differences between changepoint and oddball trials (Pearson’s *t*-test: r_59_ = −0.29; two-sided *P* = 0.027).

Our latent states theory of arousal makes different learning predictions for changepoint and oddball conditions because these conditions differ in their transition structure; namely, a changepoint reflects a deviation from expectations that is likely to persist for many trials, whereas an oddball reflects a deviation that is unlikely to persist. Or, through the lens of latent states, the key difference between oddballs and changepoints is that an additional latent state transition is required after an oddball event but prior to making a prediction for the next trial (Fig. 5a,b). This transition is adaptive for learning in that it allows the oddball to be treated differently from both the outcomes that preceded it and the outcomes that follow it. Since this transition is not locked to an external event, we anticipated that its timing would vary across trials and individuals, making it difficult to quantify with an event-locked ERP^26^. However, pupil dilations, which provide a lagged and temporally smeared reflection of transient arousal responses, provide an ideal way to test this idea. In particular, they allow us to test our fourth preregistered prediction, that pupil dilations between perceptual reports and the subsequent prediction would be greater for oddballs than for changepoints^26^.

We tested whether pupil dilations were greater for oddballs than for changepoints when aligned to the subsequent prediction. Consistent with our hypothesis, there was a negative effect of STP × condition on pupil size, indicating that pupil dilations were larger following state transitions in the oddball condition (Fig. 5c; cluster mean *t*_59_ = −2.48; two-sided *P* = 0.039; cluster forming threshold, 0.025; cluster mass, 3600; Cohen’s *d* = −0.323). This effect was strongest for the participants who showed the greatest modulation of learning behaviour across the two conditions. In particular, individuals with higher PE × STP × condition coefficients from the behavioural regression, which measured the degree of learning adjustments across the two conditions, also tended to have larger pupil dilations after oddball than after changepoint events (Fig. 5d; Pearson’s *t*-test: r_59_ = −0.29; two-sided *P* = 0.027; 95% CI, −0.50 to −0.03). Taken together, these results confirm our prediction that individuals who understand the temporal structure of the two conditions undergo a second internal latent state transition corresponding to the anticipation of a return to the previous state after an oddball event.

**Extended Data Fig. 7:**
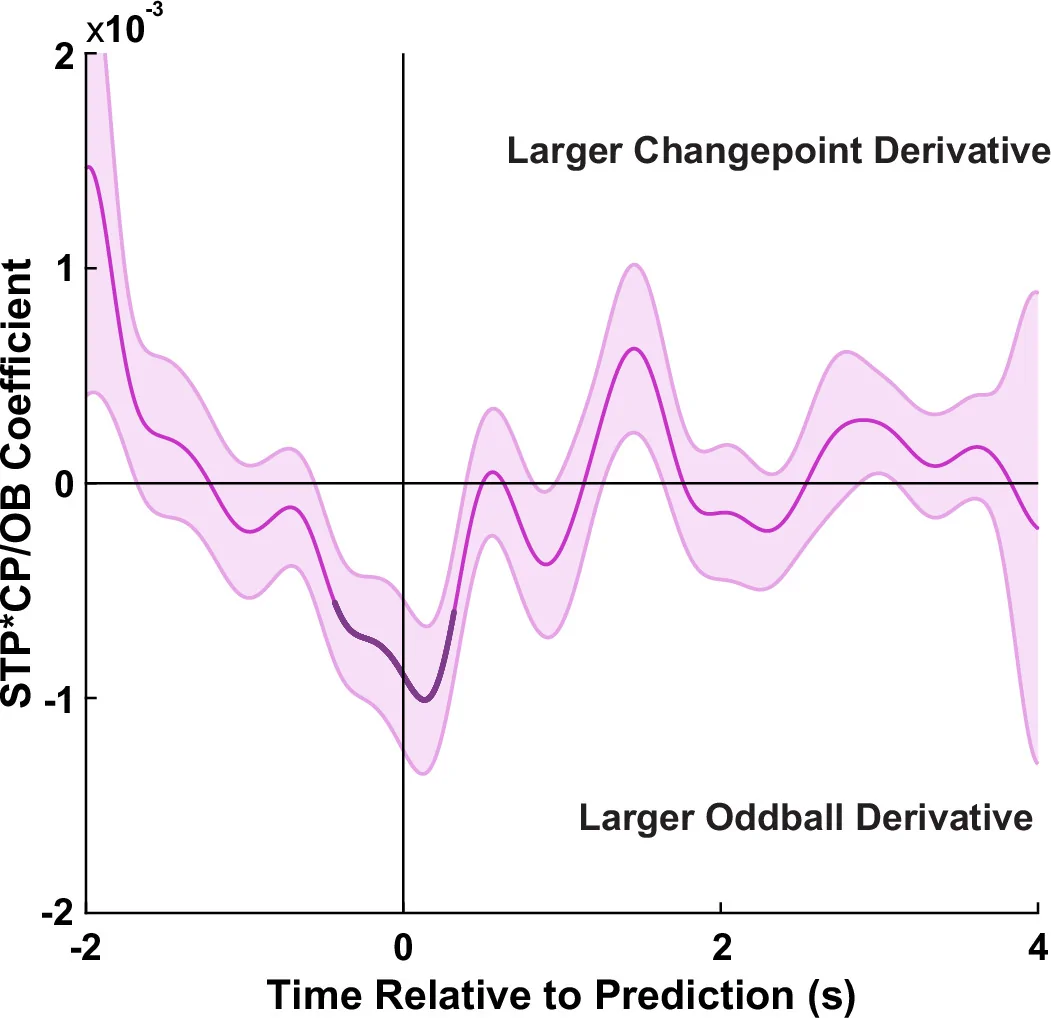
Derivative analysis of pupil dilation at the time of prediction shows dilation starts before predictions are selected. Mean/SEM STP*condition coefficients from a running linear regression of pupil size derivative locked to the prediction phase of the trial. Dark points reflect significant clusters after permutation testing for multiple comparisons correction (STP*CP/OB cluster forming threshold = 0.025, Cluster mean t(59) = 2.69, two-sided p = 0.010, Cluster mass = 1940, Cohen’s d = 0.350). The significant coefficient before the time of prediction indicated that a state transition signal from LC would have to arise before the prediction as well.

It is noteworthy that the timing of this effect occurred 200–1,500 ms after the prediction, raising questions about whether it could reflect an underlying neural process sufficiently early to influence that prediction (Fig. 5c). Given that LC spiking precedes peak pupil dilation by 600–800 ms depending on experimental conditions in monkeys^17^, this pupil dilation would correspond to a difference in LC activity across our conditions emerging 400 to 600 ms before participants indicated their updated prediction, suggesting that, at least in theory, the effect we observe in the pupil could reflect firing in the LC early enough to influence the prediction. Consistent with this idea, reanalysing the same pupil data in terms of their derivative, which follows LC activation with a shorter lag, yielded effects that preceded rather than followed the prediction (Extended Data Fig. 7). The oddball > changepoint effect was more tightly locked to the prediction than to the reproduction that preceded it, as the same analysis aligned to the preceding reproduction yielded no reliable differences. Taken together, these results support a nuanced prediction of our overarching theory that arousal systems optimize behaviour by signalling latent state transitions, in particular that oddball events elicited an additional delayed pupil response consistent with a second internally generated state transition.

### Individual differences supported a single mechanism linking arousal to bias and learning

**Fig. 6:**
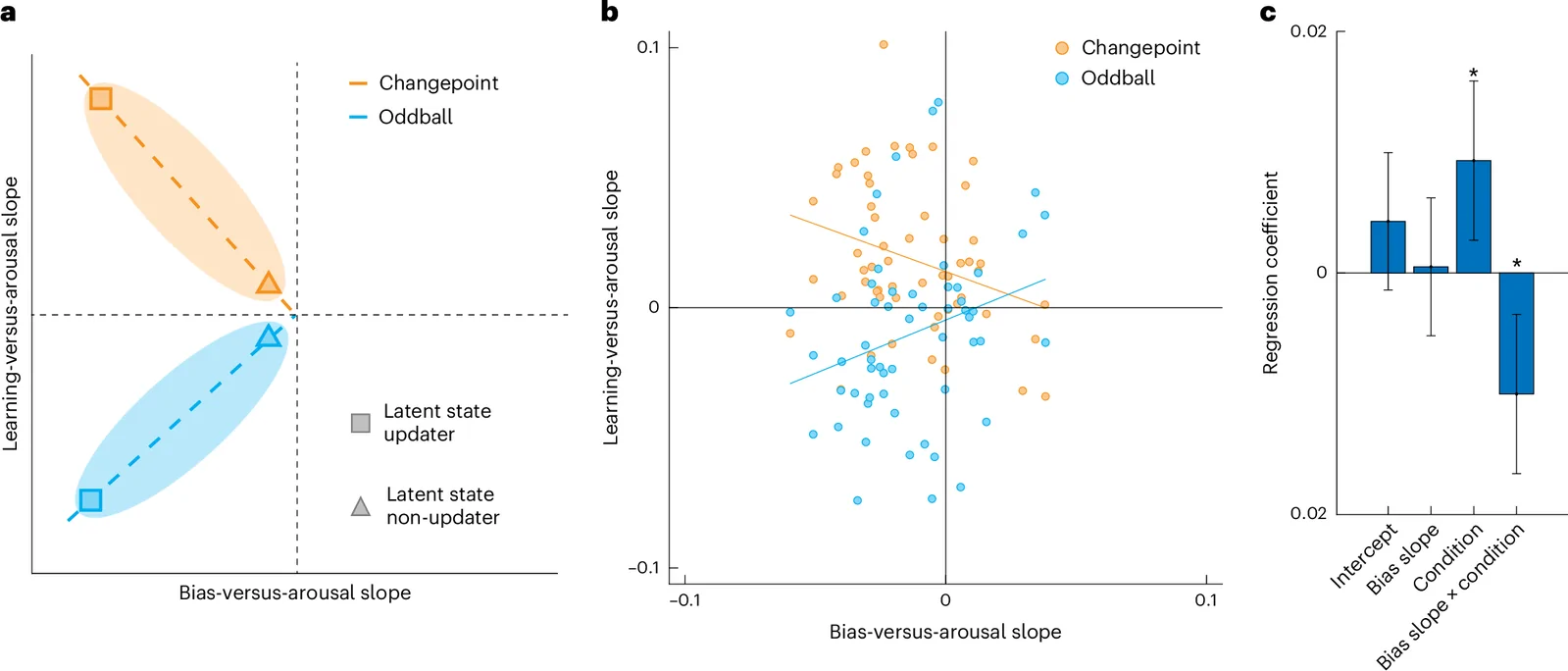
Individual differences between participants showed that those with strong relationships between learning and arousal tended to have strong relationships between bias and arousal. If the arousal markers are related to both perceptual bias and learning because they reflect state transitions that influenced both, then the degree to which a participant showed one relationship should predict the degree to which they showed the other. **a**, Hypothesized relationships between learning-versus-arousal slope (ordinate) and bias-versus-arousal slope (abscissa) for changepoint (orange) and oddball (blue) blocks. **b**, Participant data. The blue points indicate participants’ slopes in the oddball block; the orange points indicate slopes in the changepoint block. While learning-versus-arousal slopes were individually calculated within each block, bias-versus-arousal slopes were calculated across both blocks. **c**, Regression results against learning slopes. The significantly negative effect of bias slope × condition suggests the learning and bias signals shared a similar origin, as participants with stronger learning/arousal signals also had strong bias/arousal signals (condition regression coefficient: *t*_108_ = 2.79; two-sided *P* = 0.006; Cohen’s *d* = 0.268; *n* = 56; bias slope × condition regression coefficient: *t*_108_ = −3.01; two-sided *P* = 0.003; Cohen’s *d* = −0.290; *n* = 56). The error bars indicate 95% CIs.

If arousal influences behaviour by acting to signal or promote latent state transitions, then it should affect both bias and learning, and thus the degree to which it is engaged to modulate bias should be predictive of the degree to which it is engaged to modulate learning. Since participants vary in the strengths of their relationships between arousal and learning/bias, we could use these individual differences to test our theory that both arousal–behaviour effects emerge as a result of a single unitary phenomenon. If learning and bias are both driven by the same arousal-mediated computation, then the degree to which arousal signals reflect learning in an individual should be related to the degree to which the same signals reflect perceptual bias in that individual. Taking the bidirectional learning relationships into account, this leads to our fifth and final preregistered prediction: there should be a stronger difference in the learning-versus-arousal slope in the changepoint and oddball blocks for individuals with a stronger relationship between bias and arousal^26^ (Fig. 6a).

Examining individual differences in arousal’s relationship to learning and bias provided support for these basic predictions. There was a negative relationship between the learning/arousal relationship and the bias/arousal relationship in the changepoint block, implying that in the changepoint block participants with a stronger negative relationship between bias and arousal also had a stronger positive relationship between learning and arousal (Fig. 6b). To quantify the effect of the bias/arousal slope on the learning/arousal slope, we performed a regression on the learning/arousal slope. We found that there was a positive effect of condition on the learning/arousal slope, implying that, as predicted, the learning/arousal slope was higher for the changepoint than for the oddball condition. Furthermore, the relationship between arousal and bias was differentially related to that between arousal and learning depending on condition, as the relationships in the changepoint condition were more positive and those in the oddball were more negative (Fig. 6c; bias slope × condition *t*_108_ = −3.01; two-sided *P* = 0.003; Cohen’s *d* = −0.290; 95% CI, −0.017 to −0.003). This relationship was maintained even when STP was regressed out of the joint pupil–EEG arousal signal, trial average bias and trial average learning rate, which suggests that these relationships were not solely explained by the inherent relationships between STP, learning rate and bias (Extended Data Fig. 5c,d; bias slope × condition *t*_108_ = −2.67; two-sided *P* = 0.009; Cohen’s *d* = −0.257; 95% CI, −0.013 to −0.002). Taken together, these results provide evidence for a common source of arousal’s effects on learning and bias, a key prediction of our latent states theory of arousal.

## Discussion

Our results provide support for the theory that arousal systems in the brain signal the need for updating mental contexts that serve as internal latent states, leading to the optimization of learning and perceptual bias dynamics. Pupil dilations and EEG signals including the P3a, a subcomponent of the P300, were elevated in response to surprising outcomes and reflected STP across different environmental contexts. We found that pupil- and EEG-derived STP signals were related to ongoing perceptual processing, with increased arousal signatures linked to lower bias in colour reproductions. Furthermore, we found that the same transient arousal signatures were bidirectionally linked to learning, predicting heightened learning in an environment with changepoints and reduced learning in an environment with oddballs. We also found that pupil dilations preceding predictions in the task were increased for oddballs relative to changepoints, consistent with reflecting the internally generated latent state transition necessary to return to the latent state associated with typical trials before making a prediction. The size of this dilation event was also found to be related to a participant’s learning rate differences between oddballs and changepoints. Finally, we showed that individual differences in the relationship between arousal and perception bidirectionally and contextually predict those between arousal and learning, consistent with both relationships being mediated by a single common cause, the degree to which participants relied on arousal systems to reset latent states at discontinuities in the data stream. Taken together, these results provide strong support for our latent states theory of arousal: that transient signatures of arousal signal the need to update internal latent state transitions, which in turn leads to the optimization of learning and perceptual bias dynamics to minimize the negative effects of interference on behaviour.

Our theory and results provide a unifying framework for two prominent lines of inquiry related to arousal and its effects on behaviour. First, they account for relationships between arousal and learning, in particular findings that pupil dilations and P300 signals predict the degree to which people use new experiences to update their beliefs about the world^2,29,30,53,54^. However, our results demonstrate the need for reinterpretation of these original results since they reveal that the relationship between arousal signals and learning are both bidirectional and contextual. The signals co-occur with increased learning when latent state transitions tend to persist (changepoints) but with decreased learning when such transitions are fleeting (oddballs). Second, our work relates to a line of research showing that arousal signals correspond to reductions in perceptual and decision biases^20,23,24^. Our empirical results expand on these lines of work by demonstrating parallels across EEG and pupil area in their relationships to both learning and bias, and our latent states theory not only provides an explanation for these relationships but makes unique predictions about contextual signalling differences and individual differences that were confirmed in our dataset. A primary contribution of our study was thus to unify two lines of behavioural research into a single set of behaviours that correspond to latent state transitions and in turn relate them to transient markers of arousal.

Our results also highlight strong relationships between task behaviour and the strength of the arousal relationships. Although optimal task performance required adjusting learning across changepoint and oddball blocks, many participants behaved similarly across these two conditions (note the variability in PE × STP × condition coefficients in Fig. 4d), failing to dynamically adjust learning behaviour appropriately. Arousal signatures tracked this behavioural heterogeneity, relating bidirectionally to learning only in those individuals who adjusted learning behaviour appropriately across conditions (Extended Data Fig. 6). These same individual differences in behaviour contributed to the variability that allowed us to demonstrate that the strength of bias–arousal relationships was related to learning–arousal relationships, supporting a common latent states transition mechanism (Fig. 6). Thus, while behavioural data highlight that not all participants took full advantage of the distinct temporal structures we employed, our pupil and eye-tracking data demonstrate that when they did, arousal could be used to reliably anticipate their degree of bias and learning.

These results, coupled with our motivating theory, provide a potential explanation for some of the heterogeneity in the broader literature linking arousal to measures of learning^2,29,55^. In particular, the prediction of our model, which is consistent with the individual differences in relationships observed in our study, is that two tasks or individuals that differ in their structural assumptions about the environment should have very different relationships between their arousal level and subsequent learning. This prediction could be tested explicitly in future studies that probe structural understanding directly, that measure arousal relationships as structure is learned de novo or that rely on neuroimaging to identify proxies of current and anticipated future latent states^56–58^.

We interpret our results in terms of a mechanistic role for arousal systems in updating outdated latent state representations; however, this idea is not inconsistent with broader conceptual theories about what is encoded by arousal systems. For example, many of our findings are consistent with the general notion that signatures of arousal are heightened in response to surprising stimuli^1,3,25,59^. Existing theories related to surprise (except our own^8^) do not make concrete predictions about how such signals should relate to future learning and bias in our task, but they are certainly not inconsistent with our findings, since surprise was the primary cue enabling the detection of latent state transitions in our task. One factor differentiating our latent state hypothesis from surprise-based theories is that we predicted that an internally generated latent state transition would occur subsequent to an oddball trial, reflecting a return to the typical state. We did indeed observe a pupil dilation that was unique to oddball trials and consistent in timing with the proposed internally generated transition (Fig. 5).

Another account of arousal that relates to our results is that of effort^60–62^. One could argue that more effort might be required for surprising trials that reflect latent state transitions than for more typical ones. This explanation could also extend to the pupil dilations observed during the second latent state transition predicted to occur on oddball trials since these trials have an additional memory component absent on changepoint trials. More generally, if transitioning internal representations of latent state is an effortful process, then signatures of effort should always accompany them, making these two constructs difficult to tease apart with correlative methods. Here we show that the impacts of arousal on behaviour can be well predicted by a latent state account both within and across participants, but future work manipulating arousal directly could be useful in untangling the causal structure of the relationships between arousal, latent state transitions and effort.

While our study relied on peripheral markers of arousal—specifically EEG and pupillary responses to latent state transitions—our results have implications for the neural systems that underlie them. In particular, our theoretical framework is grounded in well-established physiology, pharmacology and anatomy of the LC/NE system. LC neurons respond robustly to surprising stimuli irrespective of modality and project broadly to cortical and subcortical regions, where they release NE^12,63^. In these target regions, NE has been shown to amplify feedforward processing^64,65^ and suppress local recurrent activity^66,67^, thereby providing a mechanism by which active latent state representations might be ‘reset’ in response to new inputs^8^. Moreover, the activation or inactivation of the LC/NE system facilitates or impairs behavioural flexibility^29,68^, suggesting its involvement in adaptive state updating. Although we did not directly measure LC activity, we used pupil area and the P300 component of the EEG signal as indirect markers—both of which have been proposed as proxies for LC/NE system activation^9,11,17,69,70^.

However, these proxies are inherently non-specific. For example, pupil dilation in our task also probably corresponds to the activation of multiple neuromodulatory systems—including acetylcholine, serotonin and orexin—which each play distinct roles in modulating brain state and behaviour^69,71–75^. Thus, while our study was motivated to test a theory of arousal informed by LC/NE function^8,26^, our results may reflect dynamics of any or all of these neuromodulatory systems. Acetylcholine and serotonin have also been linked to adjustments of learning rate^25,76,77^, perhaps elevating their consideration as the possible neural basis for the latent-state-transition-like signals that we measured. It is worth noting, however, that relationships between acetylcholine and pupil diameter typically occur over a longer timescale than the transient dilations that serve as the basis of our conclusions here^75^, and that rodent optogenetic studies suggest that contributions of serotonin to pupil fluctuations are smaller than those of LC/NE^74^. Thus, while it is possible that any of these systems could underlie our observed effects, their timing and magnitude, coupled with their agreement with an existing theory of the LC, make NE a candidate neuromodulatory system of particular interest.

The non-specificity of pupil dilation also underscores the importance of converging evidence from multiple physiological signals. Here we also included simultaneous EEG recordings to examine the P300, another proxy for LC/NE activity^9,11^. Prior work has associated latent state transitions with both the early frontal (P3a) and later parietal (P3b) components of the P300, with only the P3a predicting learning outcomes after STP is accounted for^31^. Here we show that the P3a and pupil diameter both match the predictions for a latent state transition signal, including their relationships to task stimuli, learning and perceptual bias, whereas the P3b did not show any of these relationships. Our behavioural analyses offer a highly specific computational characterization of these signals, and the observed relationships between physiological responses and learning outcomes lend support to the hypothesis that the LC/NE system promotes latent state transitions. We suspect that the computation underlying these effects is carried out by the LC/NE system, and we hope that future work involving direct measurement or causal manipulation will test this hypothesis more directly.

The LSTT, when applied directly to LC/NE function, is quite closely related to two other prominent theories of LC/NE function. First, the notion that neural networks are ‘reset’ in response to LC/NE signalling to facilitate rapid adaptation, or the ‘network reset hypothesis’, was proposed by Bouret and Sara in 2005. Second, the idea that the LC/NE might be recruited in response to surprising stimuli that indicate a likely transition in the environment, or the ‘unexpected uncertainty theory’, was proposed by Yu and Dayan in 2005. The LSTT, which we used to generate our predictions and to which our experimental results lend considerable support, can be thought of as a combination of these two theories, whereby unexpected events are detected and used to reset neural networks that represent the latent state of the world. While the latent states theory is compatible with these other theories, it has advantages in terms of specificity and generality. In particular, we view the latent states theory as complementing the network reset hypothesis by providing more specific predictions about exactly how neural networks should be updated to optimize behaviour^14^, and complementing the unexpected uncertainty theory by generalizing it to a broader range of generative environments beyond those for which it was originally mathematically derived^25^.

While our results largely fall in line with the latent states theory that motivated our predictions, there were several findings that were unanticipated. First, the spatiotemporal profile of the state transition signal we observed in the EEG data differed from that observed in another task that included both changepoint and oddball conditions^31^. Here we observed only one spatiotemporal cluster that resembled the P300. While previous work identified a state transition signal that included both early frontal (P3a) and later parietal (P3b) components, in the current work we found that only the former was related to state transitions. However, although previous work linked both the P3a and P3b to state transition signals, only the early component of the P300 (P3a) was related to learning after we controlled for other factors including STP itself. Taken together with our current findings, this suggests that the P3a component may play a more central role in the neural mechanisms linking inferred state transitions to their behavioural consequences (compared with the P3b). We also note that our task was fundamentally different from the predictive inference task previously used to identify relationships between latent state transitions and the P300, since participants needed to use working memory to store colours on each trial, irrespective of the level of surprise or the need for updating latent states. The P3b might reflect working memory updating^68^, which is consistent with the large stimulus-locked P3b responses, irrespective of trial type, observed in our study. Thus, one possibility is that, in the absence of an explicit working memory demand, working memory updating systems can be recruited to participate in the updating of latent states, leading to relationships between the P3b and latent state updating in predictive inference tasks, but not working memory tasks, where these systems are otherwise engaged. Nonetheless, it remains unclear why, in our data, the global parietal signal was reduced on state transition trials.

The P3a has previously been observed in response to novel and surprising stimuli^78–81^ as well as related to internally mediated shifts in attention^82–84^. These situations roughly map onto the ones requiring externally triggered and internally mediated latent state transitions in our task, respectively. Indeed, through this lens, the problem of latent state updating could be thought of as an attentional control problem, where latent state representations are selected through an attentional spotlight that is controlled according to internal and external factors. Our theory and data thus extend this line of work by showing how attentional control should be, and is, applied to dynamically adjust learning and perceptual bias in temporally structured environments. This theory provides mechanistic insights that differ qualitatively from common notions of relevance within the attentional field, since our theory predicts that even though oddballs could be ignored for purposes of learning behaviour, they necessitate a latent state transition, which could be conceptualized as a shift in attention to an alternate ‘oddball’ latent state. The P3a data from our task support this mechanistic view, since oddballs elicit larger P3a responses that in turn relate to reduced learning from oddball events.

One additional discrepancy between our results and those we predicted is related to the timing of the pupil dilation that we predicted to occur in response to internal state transitions on oddball trials. We hypothesized that this second oddball signal would occur between the perceptual report and the prediction, as our theory stipulates that participants would need to switch latent states during this period to make accurate perceptual reports and subsequent predictions. Instead, we found that the pupil signal occurred slightly after participants made their predictions, which we interpreted as occurring due to the delay between the LC signalling of the state transition and the subsequent pupil dilation event, which in monkeys has been estimated at around 800 milliseconds^69^. Nonetheless, the timing of this event was later than anticipated and certainly raises the question of whether the arousal systems are activated before or after internal responses to latent state transitions, which would be best investigated using more direct and time-resolved measures of LC/NE function.

In summary, our results support the overarching idea that the brain optimizes learning and perception through dynamic transitions of internal latent state representations. Transient markers for arousal, including pupil area and the P3a, reflect these transitions and relate to learning and bias behaviours accordingly. These relationships appear functional, since they persist after we control for objective measures of STP, and are stronger in participants who modulated behaviour more. Taken together, our results support a role for arousal systems in coordinating the dynamics of ongoing context representations that are used to fine-tune perception and learning.

## Methods

### Ethics information

Informed consent was obtained from each participant in the study, and all procedures were performed in accordance with the Declaration of Helsinki. Each participant was compensated with US$15 per hour plus a bonus based on task performance. All protocols were approved by the Brown University Institutional Review Board (Brown University Federal Wide Assurance No. 00004460).

### Task design

To test our theory that arousal systems signal latent state transitions and their downstream behavioural consequences, we designed a colour perception and prediction task that operates in two distinct statistical environments. On each trial, participants were presented with two coloured-target stimuli for 200 ms and subsequently prompted to reproduce each colour after a delay period. The advantage of presenting two simultaneous stimuli was twofold: first, it allowed us to increase the number of surprising events that would be included in an experimental session, and second, it led to reproductions that, while related to the presented colour, were somewhat imprecise, meaning that they could be improved through the combination of sensory information with prior expectations. Stimuli were prompted in a randomized order.

The task was divided into four blocks. The first block was purely for practice as participants reproduced the displayed stimuli and received feedback on the accuracy of their perceptual reports. The colours in this block were randomly generated. The second block was a longer iteration of the basic structure of the first block; however, participants did not receive accuracy reports on their individual colour estimations (although they did receive feedback at the end of the block). Given that the colours displayed in the second block were chosen randomly from a uniform distribution, behavioural data from this block were used as a control condition to better understand whether participants were able to exploit the structure in later blocks to improve their colour reproductions.

In the third and fourth blocks, participants performed the same task but modified in two important ways. First, the participants were asked to predict the colour of the stimulus that would be presented at each location on the following trial. Predictions were possible because the colours at each location were generated through an independent sequential generative process following either the changepoint or oddball state transition structure. The generative structure was instructed and explained to the participant. The third and fourth blocks were counterbalanced for order, so if a participant experienced the changepoint condition in block 3 they would experience the oddball condition in block 4, or vice versa. Predictions and estimations were considered correct and participants were awarded 1 point if the error fell below 30°. If the error was greater than 30°, participants were penalized with a deduction of 1 point. At the end of the study, participants received one bonus dollar for every 30 points that they scored up to 20 dollars (600 points). Both changepoint and oddball blocks contained 120 trials, and the task lasted 1.5 to 2 hours in total. The hazard rates for changepoint and oddball were both set to 0.15, and given that each trial had two stimuli that underwent independent generative processes, participants experienced an average of 36 changepoints or oddballs per block (s.d. 6).

The colours in our task were chosen from a circle in CIELAB colour space (radius in A*; B* = 50; white point, [110, 100, 90]) along a plane with fixed luminance (L* = 60). Standardized colours were shown by characterizing the experimental display using methods standard in colour perception research^85,86^. The CIELAB colour space was chosen for its approximate perceptual uniformity, which allows us to interpret angular errors in terms of fixed units of perceptual difference. The luminance of our colour circle was fixed to minimize stimulus-related variability in the pupillary light reflex. All stimuli were displayed on a background with chromaticity equal to the white point with luminance similar to the luminance of the stimuli (L* = 50). Colours are referred to by angular position in the task for convenience. All human participant procedures were approved by the Brown University Institutional Review Board.

### Task generative model

Stimulus colours for each location were determined according to one of two generative models reflecting either the changepoint or oddball condition (Extended Data Fig. 1). In both cases, a generative mean (*M*) with a value between 0 and 360 was sampled to reflect the underlying mean of the colour distribution (*M*_*t*_ ∼ U(0,360)). *M* served as the mean of a von Mises distribution across a colour spectrum from which a colour value (*X*) was drawn (${X}_{t}\sim {\mathrm{N}}\left({M}_{t},{\sigma }_{X}^{2}\right)$), where ${\sigma }_{X}^{2}=10$. Beyond the observable aspects of the task, we assumed that subjective internal representations of the stimulus value were corrupted by noise and thus drew an internal representation (*Y*) from a normal distribution centred on *X*, which we refer to as sensory noise (${Y}_{t}\sim {\mathrm{N}}\left({X}_{t},{\sigma }_{Y}^{2}\right)$). Thus, while *X* reflected the colour shown in our task, *Y* reflected the subjective internal representation of that colour accessible by the participant. To constrain the degree to which internal representations were corrupted, we set ${\sigma }_{Y}^{2}$ according to the measured variability in participant reports during the unstructured control task block. We found that the root mean squared estimation error was ~31° in the unstructured control block, and thus for our simulations we set ${\sigma }_{Y}$ equal to 31°.

The value of *M* transitioned across trials in one of two possible ways depending on the condition in each block. In the changepoint condition, a binary variable *S* was sampled according to a Bernoulli distribution with probability *H*, 0.15, for hazard rate. If *S* was equal to 1, we drew the next generative mean from a uniform distribution. Otherwise, we drew the next generative mean from a normal distribution whose mean was the generative mean of the previous trial, leading to the conditional sampling statement below:

$$\begin{array}{cccc}{\mathrm{If}}\;S=1\\{M}_{t}\sim {\mathrm{U}}\left(0,360\right)\\{\mathrm{otherwise}}\!\!:\\{M}_{t}={M}_{t-1}\end{array}$$

In either case, the true stimulus value was drawn from the current generative mean:

$${X}_{t}\sim {\mathrm{N}}\left({M}_{t},{\sigma }_{X}^{2}\right)$$

The generative model for the oddball condition differed from that for the changepoint condition in two important ways. First, *M* transitioned according to a Gaussian random walk across all trials (${M}_{t}\sim {\mathrm{N}}({M}_{t-1},{\sigma }_{{\rm{\mu }}}^{2})$), rather than changing at discrete changepoints. Second, the colour on each trial was chosen conditionally according to *S*, such that if *S* was equal to 1, then the colour (true outcome: *X*_*t*_) was chosen uniformly across the entire colour wheel; otherwise, the colour continued to be drawn from the generative mean of the trial (*M*_*t*_). In the oddball block, if *S* was equal to 1, *M*_*t*_ only changed according to the Gaussian random walk. This gave rise to the following conditional sampling statements:

$$\begin{array}{ccccc}{\mathrm{If}}\;S=1\\{X}_{t}\sim {\mathrm{U}}\left(0,360\right)\\{\mathrm{otherwise}}\!\!:\\{M}_{t}\sim {\mathrm{N}}\left({M}_{t-1},{\sigma }_{{\rm{\mu }}}^{2}\right)\\{X}_{t}\sim {\mathrm{N}}\left({M}_{t},{\sigma }_{X}^{2}\right)\end{array}$$

### Bayesian inference model

Our inference model assumed that participants understood the generative structure of the task but had access only to the observable parts of the generative model (*Y*_1_, *Y*_2_, *Y*_3_…*Y*_*t*_). We assumed that participants used this sequence of corrupted internal representations to infer the mean of the colour distribution, *M*_*t*_, which could be used as a prediction on each trial, and estimated the colour of the observed stimulus, *X*_*t*_, which could serve as a perceptual report on each trial.

The inference model was derived by inverting the generative model (Extended Data Fig. 1) to infer *M* and *X* on the basis of previous observations:

1 $$p({M}_{t},\,{X}_{t}|{Y}_{1:t})\propto \mathop{\sum }\limits_{{M}_{t-1}}\mathop{\sum}\limits_{{X}_{t-1}}\mathop{\sum}\limits_{{S}_{t}}\,p({Y}_{t},\,{M}_{t},\,{X}_{t},\,{M}_{t-1},{X}_{t-1},\,{Y}_{1:t-1},\,{S}_{t})$$

Note that this expression included summations over the mean on previous trial (*M*_*t*−1_), the colour on the previous trial (*X*_*t*−1_) and the possible state transitions on this trial (*S* = 1 corresponds to changepoint or oddball depending on the condition; *S* = 0 corresponds to a standard transition). In the changepoint condition, conditional independencies from the generative graph were exploited to yield the following factorization:

2 $$\begin{array}{l}p\left({M}_{t},\,{X}_{t}|{Y}_{1:t}\right)\propto \\ \mathop{\sum}\limits _{{M}_{t-1}}\mathop{\sum}\limits _{{X}_{t-1}}\mathop{\sum}\limits_{{S}_{t}} p\left({Y}_{t}{{|}}{X}_{t}\right)\ p\left({X}_{t}|{M}_{t}\right)\ {p}\left({M}_{t}|{M}_{t-1,}{S}_{t}\right)\ p\left({M}_{t-1},\,{X}_{t-1}{|}\,{Y}_{1:t-1}\right)\, p({S}_{t})\end{array}$$

where *S*_*t*_ reflected the prior probability that any given trial will be a changepoint, referred to in the text as the hazard rate. Conditional independencies in the oddball condition yielded a slightly different factorization:

3 $$\begin{array}{l}p\left({M}_{t},\,{X}_{t}|{Y}_{1:t}\right)\propto \\ \mathop{\sum}\limits _{{M}_{t-1}}\mathop{\sum}\limits _{{X}_{t-1}}\mathop{\sum}\limits_{{S}_{t}}p({Y}_{t}|{X}_{t})\,p({X}_{t}|{M}_{t,}{S}_{t})\,\ p({M}_{t}|{M}_{t-1})\ p({M}_{t-1},\,\,{X}_{t-1}|\,{Y}_{1:t-1})\ p({S}_{t})\end{array}$$

In both cases, the model inferred *M*_*t*_ and *X*_*t*_ on the basis of the observed history of *Y*, and this inference serves as a prior for a Bayesian update on the subsequent trial. The model produced an estimate of the stimulus shown by reporting the maximum posterior probability estimate of the stimulus (*X*_*t*_). Note that since inference included not only the pure sensory observation *p*(*Y*_*t*_|*X*_*t*_) but also prior information based on learning of the underlying mean (terms 2–4 in equation (3)), the Bayesian inference model made estimates about the stimulus that were biased towards its prior expectations, but to a degree that respected the generative structure of the task that included occasional abnormal events (*S*). The inference model obtained its prediction of the next stimulus from the maximum posterior probability estimate of the generative mean (*M*_*t*_).

STP estimates were computed as the integrated probability that a given observation reflected a latent state transition (*S*_*t*_), which reflected the model’s estimate of changepoint probability or oddball probability depending on the condition. This measure was monotonically related to standard measures of surprise, such as Shannon information, but differed in that it saturated at the point where a state transition is certain.

Trial-to-trial measures for STP and learning extracted from the model were dissociable across conditions: positively correlated in the changepoint condition and negatively correlated in the oddball condition. This feature allowed us to leverage complementary conditions that disentangle normative influences on learning and perceptual bias.

Previous studies have shown that uncertainty affects learning such that learning rate experiences slow decay after state transitions. Here we measured uncertainty as the entropy on the model’s inference about the underlying mean, which is highest on trials after state transitions.

### Data collection and preprocessing

We collected behavioural, EEG and pupillometry data from 63 healthy adult participants (33 female; mean age, 24.43 years; s.d. = 5.58; range, 18–40 years) while they performed the task. EEG data were recorded using a Brain Vision actiCHamp Plus 64 system at 1,000 Hz, and pupil data were recorded at 1,000 Hz using an SR Research Eyelink 1000 Plus eye tracker. Participants were recruited through the Brown University SONA system, university mailing lists and newsletter advertisements. Of the total sample, behavioural data were available for all 63 participants, 60 had usable pupil data, 57 had usable EEG data and 56 had usable data for both modalities (see the criteria below for data inclusion). Analyses were not disaggregated by sex, as the study did not address sex-specific hypotheses related to the physiological markers.

EEG preprocessing was performed in EEGLAB (v.2024.2.1) with the EYE-EEG extension for eye-data-guided independent components analysis^87^. We used a high pass filter of 0.1 Hz to remove low-frequency noise and then segmented the data into 300 epochs, each containing the two seconds before and after a trial. Each individual trial and channel data were then visually inspected, and channels with persisting noise or artefacts were removed and interpolated. Two participants were unable to complete the task while EEG data were recorded, and one EEG dataset was not recorded due to researcher error. Participants with >50% rejected trials or >25% rejected channels were excluded from further analysis (*n* = 3). Next, we re-referenced each channel’s data according to the mean signal. Independent component analysis was then performed on the data. We used the EYE-EEG extension to remove components with higher correlation to eye blinks and saccades than some threshold. We then low pass filtered the EEG data at 30 Hz. The cleaned data were then inspected manually to identify and remove any remaining trials with excessive noise or artefacts.

Eye-tracking data were preprocessed using a custom MATLAB script. First, we removed any data from eyes that were poorly recorded. For participants where one eye’s data was markedly worse than the other’s, only the data from the eye with better recordings was used. Next, we linearly interpolated eye data during blinks and during a window 150 ms before and after each blink. This also filled in any gaps in eye data recording that were created by the eye tracker failing to track either eye. The data were then averaged across both eyes and epoched according to the time window that was being investigated. Trials with >20% missing data were rejected for all pupil-only analyses. Trials with >50% missing data were rejected for joint pupil–EEG analyses. We set this higher threshold for the joint analyses to retain as many trials as possible, since many trials had already been rejected during the EEG preprocessing pipeline. Participants with more than 50% missing data on 50% or more of the trials were rejected from eye data analysis entirely (*n* = 3).

### Behavioural data analysis

To examine how latent variables from our model influence learning and perceptual bias, we generated two linear regressions. Through these regressions, we analysed how STP modulates learning and perceptual bias.

To examine learning, we first computed trial-to-trial prediction update as colour prediction(*t* + 1) − colour prediction(*t*). Learning rate was then measured at the individual participant level as the linear relationship between prediction update and PE in three different complementary ways. One method measured stimulus-specific learning, one quantified overall learning for the entire trial across both stimuli and a final method used a regression that included all trials to determine how learning rate varied with task parameters. The first method, which assessed stimulus-specific learning, was simply to divide prediction update by sPE. For this method, to account for the circular nature of the colour space, trials with updates near 180° were adjusted so the update and error had the same sign, which enabled more accurate estimation of the relationship between STP and learning rate in synthetic data generated by the Bayesian model. Second, when comparing learning rate to the EEG/pupil signal, we combined measurements of learning from both stimuli into a single value by taking the slope of a line fit to a point on the origin and the two stimuli plotted on a coordinate system containing sPE as the abscissa and the prediction update as the ordinate. This method for computing single-trial learning rates was also validated using simulated data: learning rates calculated by applying this method to simulated data from a Bayesian ideal observer model were more strongly correlated with STPs than those computed using other methods, supporting the idea that it could be used to reveal the single-trial learning consequences of latent state transitions. The third method of measuring participant and model learning rates relied on a linear regression model. The regression model explained the entire set of updates (outcome − prediction) from a single session in terms of PEs (capturing average learning rates across the session) as well as in terms of interactions capturing how participants modulated the influence of PEs according to variables of interest. PE, in this case, was defined as the sPE. The sPE was the difference between the trial stimulus colour reproduction (a reflection of what the participant believed they saw) and the colour prediction on the trial before stimulus onset. The full regression model took the following form:

$$\begin{array}{lll}{\rm{Prediction\; update}}={\beta }_{0}+{\beta }_{1}\left({\rm{sPE}}\right)+{\beta }_{2}\left({\rm{sPE}}\right){\times }\left({\rm{STP}}\right){\times }({\rm{condition}})\,\\\qquad\qquad\qquad\quad\qquad+{\beta }_{3}\left({\rm{sPE}}\right){\times }\left({\rm{STP}}\right)+{\beta }_{4}\left({\rm{sPE}}\right){\times }\left({\rm{condition}}\right)\\\qquad\qquad\qquad\qquad\quad+{\beta }_{5}\left({\rm{sPE}}\right){\times }\left({\rm{entropy}}\right)+{\beta }_{6}\left({\rm{sPE}}\right){\times }\left({\rm{model\; mismatch}}\right)\\\qquad\qquad\qquad\qquad\quad+{\beta }_{7}\left({\rm{sPE}}\right){\times }\left({\rm{stimulus\; side}}\right)+\varepsilon \end{array}$$

where condition is a binary dummy variable with changepoint assigned as 1 and oddball assigned as −1. *β*_1_ is a term that captures the average rate of learning. *β*_2_ is an interaction term between STP, sPE and condition that captures the bidirectional learning effect predicted by our model: increased learning for state transitions in the changepoint condition and decreased learning for state transitions in the oddball condition. *β*_3_ measures how STP alone changes the slope of average learning rate. The error term, *ε*, was assumed to be independent and von Mises distributed to account for the nature of the circular colour wheel in our task.

The model also included nuisance variables that capture variance unrelated to our primary hypotheses. *β*_4_ captures how the slope of the average learning rate adjusted depending on task condition. *β*_5_ describes how entropy or trial-to-trial uncertainty affected the slope of average learning rate. Model mismatch captures trials where participant predictions diverged from model predictions. To calculate this term, we fit the differences between model and participant prediction for each trial under the same task structure with a mixture of Gaussian and uniform distributions with mixture proportion and precision as free parameters. Model mismatch is the likelihood for each trial that the participant prediction came from the uniform component of the mixture distribution.

We also calculated bias in three ways similar to our methods of calculating learning rate. First, perceptual bias was measured at the individual stimulus level as the slope of the relationship between reproduction error, taken as the difference between the ground-truth colour and the perceptual report, and PE. PE, in this case, was defined as the oPE, which is the difference between stimulus colour prediction and the ground truth of the same stimulus colour on the same trial. Second, when comparing bias to the EEG/pupil signal, we combined the bias measurements into a single value by taking the slope of a line fit to a point on the origin and the two stimuli plotted on a coordinate system containing oPE as the abscissa and the reproduction error as the ordinate. Applying this method of bias calculation to synthetic data from our Bayesian model showed stronger correlations with latent STP than did our single-stimulus bias measure (absolute average bias), and thus we relied on this measure to test the link between arousal and bias adjustments. Third, to understand how perceptual bias was affected by state transitions, we performed the following regression on both the model and participant behaviour data:

$$\begin{array}{lll}{\rm{Reproduction\; error}}={\beta }_{0}+{\beta }_{1}({\rm{oPE}})+{\beta }_{2}({\rm{oPE}})\times({\rm{STP}})\\\qquad\qquad\qquad\qquad\quad\,\,\,\times\,({\rm{condition}})+{\beta }_{3}({\rm{oPE}})\times\left({\rm{STP}}\right)\\\qquad\quad\quad\quad\qquad\qquad\,\,\,\,+\,{\beta }_{4}\left({\rm{oPE}}\right)\times({\rm{condition}})+{\beta }_{5}({\rm{oPE}})\\\qquad\quad\quad\quad\qquad\qquad\,\,\,\times\,({\rm{entropy}})+{\beta }_{6}({\rm{oPE}})\times({\rm{model\; mismatch}})\\\qquad\quad\quad\quad\qquad\qquad\,\,\,+\,{\beta }_{7}({\rm{oPE}})\times({\rm{gaze\; attention}})+{\beta }_{8}({\rm{oPE}})\\\qquad\quad\quad\quad\qquad\qquad\,\,\,\times\,({\rm{stimulus\; side}})+{{\varepsilon }}\end{array}$$

where *β*_1_ captures the overall degree of perceptual bias, and *β*_3_ captures the degree to which such biases are suppressed at likely state transitions. Note that this regression is similar to the regression for learning, with an additional *β*_7_ term, the dependent variable changed to reproduction error and the PE term changed to oPE. The nuisance variable (*β*_7_) captured the degree to which average horizontal gaze position during stimulus onset (0–200 ms) affected the relative bias in perceptual reports related to the right and left target colours. The gaze attention variable is zero for both targets when centrally fixated, negative if gaze is towards the target and positive if gaze is away from the target. The coefficient (*β*_7_) estimated the contribution of the interaction between the relative gaze attention and PEs on perceptual errors, allowing us to capture variability in bias that might have related to differences in visual attention.

To verify that participants’ perceptions were biased in the prediction blocks and that this bias was comparable to what would be expected given Bayesian combinations of priors (predictions) and likelihoods (colour presentations), we calculated the theoretical variance that would be expected on perceptual reports that reflected Bayesian integration of prior information (predictions) and likelihood information (unbiased perceptual reports from the unstructured block). To do so, we first calculated the variance of participant PEs, as a measure of uncertainty on prior probabilities of an upcoming stimulus. Next, we calculated the variance on reproduction errors made during the unstructured practice block as a measure of the likelihood (unbiased percept). We then calculated the variance expected for a weighted combination of these sources of information using the equation below:

4 $${\sigma }_{{\mathrm{Predicted}}}^{2}=\frac{1}{\frac{1}{{\sigma }_{{\mathrm{EstErr}}}^{2}}+\frac{1}{{\sigma }_{{\mathrm{PredErr}}}^{2}}}$$

To examine whether this theoretically derived measure differed from participant reproduction errors made in the predictable task blocks, we then performed a *t*-test comparing the predicted to actual task reproduction error variances.

### Pupil and EEG data analysis

Both pupil area and EEG signals were regressed onto an explanatory matrix that contained the following:

$$\begin{array}{ll}{\rm{EEG}}\; {\rm{Data}}={\beta }_{0}+{\beta }_{1}({{{\rm{mean}}\; {\rm{STP}}}})+{\beta }_{2}({\mathrm{mean}}\,{\mathrm{STP}})\\\qquad\qquad\quad\,\,\,\times\,({\mathrm{condition}})+{\beta }_{3}({{\rm{mean}}\; {\rm{entropy}}})+{\beta }_{4}({\rm{condition}})\\\qquad\qquad\quad\,\,\,\,+{\beta }_{5}({\rm{baseline}}\; {\rm{EEG}})\end{array}$$

$$\begin{array}{lll}{\mathrm{Pupil}}\,{\mathrm{Data}}={\beta}_{0}+{\beta}_{1}({\mathrm{mean}}\,{\mathrm{STP}})+{\beta}_{2}({\mathrm{mean}}\,{\mathrm{STP}})\\\qquad\qquad\quad\,\,\,\times\,({\mathrm{condition}})+{\beta}_{3}({\mathrm{mean}}\,{\mathrm{entropy}})\\\qquad\qquad\quad\,\,\,+\,{\beta}_{4}({\mathrm{condition}})+{\beta}_{5}\left(\sin ({\mathrm{left}}\,{\mathrm{colour}})\right.\\\qquad\qquad\quad\,\,\,\left.+\,\sin ({\mathrm{right}}\,{\mathrm{colour}})\right)+{\beta }_{6}\left(\cos ({\mathrm{left}}\,{\mathrm{colour}})\right.\\\qquad\qquad\quad\,\,\,\left.+\,\cos ({\mathrm{right}}\,{\mathrm{colour}})\right)+{\beta}_{7}({\mathrm{baseline}}\,{\mathrm{pupil}}\,{\mathrm{area}})\end{array}$$

To further minimize the effects of the presented colour on pupil dilation, we also included the sum of the sines and cosines of stimulus colours as nuisance regressors in the pupil data regression. All regressors and pupil data were mean centred and normalized before regression. EEG voltages were not normalized in order to retain our original units of microvolts. Normalized baseline pupil area and EEG voltage were included in the regressions to account for differences across trials. For the EEG regression, we averaged channel voltage across the 200 ms before stimulus onset to calculate our baseline value. For the pupil regression, we averaged pupil size across the 1,000 ms preceding stimulus onset. Since we did not subtract the baseline, mean coefficients may be different from zero during the baseline windows we used. Regression coefficients were combined across participants to compute *t* statistics at each time point (and channel in the case of EEG). The *t* statistics were thresholded with a cluster-forming threshold corresponding to *P* = 0.01 for EEG data and *P* = 0.025 for pupil data, and temporal or spatiotemporal clusters were submitted to permutation testing to identify clusters with mass (summed *t* statistic within cluster) that exceeded 97.5% of values in the permuted null distribution.

Trial-to-trial pupil and EEG signal strength was quantified within windows of significant association with STP as the dot product of baseline-regressed individual trial responses and the thresholded *t*-statistic map over time (for pupil analysis) or time and channels (for EEG analysis). Trial-to-trial measures of pupil and EEG response were incorporated into two regression models to explain learning rate and bias in the changepoint and oddball blocks. These regressions were run for each participant separately, and coefficients for all participants were compared against zero using a Student’s *t*-test to determine the significance of relationships between learning/bias and arousal. In addition to this statistical testing, we qualitatively tested our latent state predictions. To visualize our results, we sorted pupil dilation and P300 data into five quantiles and computed and plotted the perceptual bias and learning rates as described above separately in each quantile.

We also examined pupil size during the prediction phase of the task in two different time windows to test whether oddballs promote higher levels of arousal on the subsequent trial than changepoints. Pupil size in a time window beginning one second before the end of the colour estimation phase and ending five seconds later was regressed onto an explanatory matrix. This explanatory matrix was as follows:

$$\begin{array}{ll}{\rm{Pupil\; data}}={\beta }_{0}+{\beta }_{1}({\rm{mean\; STP}})+{\beta }_{2}({\rm{mean\; STP}})\times({\rm{condition}})\\\qquad\qquad\,\,\,\,\,\,\,\,\,+{\beta }_{3}({\rm{mean\; entropy}})+{\beta }_{4}({\rm{condition}})\\\qquad\qquad\,\,\,\,\,\,\,\,\,+{\beta }_{5}({\rm{baseline\; pupil\; area}})\end{array}$$

Note that the colour terms were removed from this regression because the time window we were investigating did not include stimulus presentation. Significance was assessed by computing *t* statistics across participants and controlling for multiple comparisons using permutation testing as described above.

We also assessed pupil size during a six-second window from two seconds before each prediction was made during a trial to four seconds after the prediction was made. Since participants’ eye data were of lower quality during this more demanding period of the task and participants were often squinting, we removed any trials where a participant’s pupil size was more than six standard deviations smaller than the participant’s mean pupil size. Pupil size during this time window was regressed onto an explanatory matrix that contained the following:

$$\begin{array}{l}{\rm{Pupil\; data}}={\beta }_{0}+{\beta }_{1}({\rm{STP}})+{\beta }_{2}({\rm{STP}})\times({\rm{condition}})\\\qquad\quad\quad\;\;\,+{\beta }_{3}({\rm{entropy}})+{\beta }_{4}({\rm{condition}})\\\qquad\quad\quad\;\;\,+{\beta }_{5}({\rm{baseline\; pupil\; area}})\end{array}$$

The STP and entropy terms for this regression were not averaged because responses were made individually. Thus, if the left stimulus was prompted, the STP term would be the STP value for the left stimulus only rather than the mean STP across both stimuli. The baseline time window was from 2,000 to 500 ms before the prediction was made. To determine whether coefficients in this regression were statistically significant across time, we performed a permutation test identical to that done on the previous pupil regression coefficients.

To assess whether prediction-locked pupil dilations were related to behaviour, we examined whether participants who had larger bidirectional relationships between learning and state transitions (that is, increased learning in response to changepoints and decreased learning in response to oddballs) also had pupil dilations that were more sensitive to the difference between the changepoint and oddball conditions. To do so, we correlated the pupil regression STP × condition coefficients from the time window when group effects differed significantly from zero with the sPE × STP × condition coefficients from the behavioural regression.

Since the timing of this event was made somewhat unclear by our baselining process, we also performed the same analysis on the derivative of the pupil area during the same time window (two seconds before prediction to four seconds after prediction). We convolved the pupil signal across a window of 150 ms, took the derivative of the convolved signal and ran the same running regression across those data. Significance was assessed by performing a *t*-test across each participant’s STP × condition coefficient and then performing a permutation test to assess temporal clusters of significant *P* values.

### Joint pupil–EEG analysis

For analyses using a single value for arousal, pupil area and EEG data were combined. The individual cluster trial scores for both the EEG and pupil data were (in the case of EEG) summed and then normalized to create one value each for pupil area and EEG that were weighted equally. These two values were then summed to create a single value corresponding to arousal during the trial. Any trials that were rejected previously in the EEG data or had a blink percentage higher than 50% were removed from the analysis. We then performed the same regression on the joint pupil–EEG arousal signal that we did on the separate EEG and pupil signals to quantify the relationship between arousal and learning or bias. To visualize the joint arousal data, we sorted each block of participant data into five quantiles and plotted the mean learning rate or bias across participants for each quantile, similarly to how we visualized the separated pupil and EEG data.

To quantify the relationship between learning/arousal slopes and bias/arousal slopes across both blocks, we calculated the bias/arousal slope combined across blocks and plotted it against the learning/arousal slopes for each block separately that had been previously calculated from the joint pupil–EEG data. Each bias/arousal value had two coordinates associated with it, as it was plotted against the values calculated for the participant’s changepoint and oddball learning/arousal slopes separately. We then performed a regression on the learning/arousal values as follows:

$$\begin{array}{ll}{\rm{Learning}}/{\rm{arousal\; slope}}={\beta }_{0}+{\beta }_{1}({\rm{bias}}/{\rm{arousal\; slope}})\\\qquad\qquad\qquad\qquad\qquad\,\,\,\,\,\,+\,{\beta }_{2}({\rm{bias}}/{\rm{arousal\; slope}})\\\qquad\qquad\qquad\qquad\qquad\,\,\,\,\,\,\times\,({\rm{condition}})+{\beta }_{3}({\rm{condition}})\end{array}$$

To determine the relationship between bias/arousal slope and learning/arousal slope, we looked for a significant value in *β*_2_; a negative relationship would show that participants with stronger learning/arousal slopes tended to have stronger bias/arousal slopes in both blocks.

We also wanted to investigate whether the relationships we found in the earlier investigations were robust even if STP was removed. To do this, we regressed mean trial STP from the trial average learning rates, trial average bias and trial-by-trial EEG and pupil signals. Next, we performed the pupil and EEG data combination method described above and repeated our analyses above to calculate learning/arousal and bias/arousal relationships in both blocks. We then performed the regression of STP-regressed bias/arousal slope against STP-regressed learning/arousal slope as described above to determine whether the relationship between bias slope and learning slope survived after regressing out STP.

### Reporting summary

Further information on research design is available in the Nature Portfolio Reporting Summary linked to this article.

## Supplementary information

Reporting Summary

Peer Review file

## Data Availability

EEG and pupillometric data are available via Dryad at https://doi.org/10.5061/dryad.wm37pvn36 (ref. ^88^). Behavioural data are available via GitHub at https://github.com/learning-memory-and-decision-lab/Li-Marble-2025.git.

## Code availability

All scripts for data collection and analysis are publicly accessible via GitHub at https://github.com/learning-memory-and-decision-lab/Li-Marble-2025.git. Task scripts were run using MATLAB v.R2019b (ref. ^89^), and preprocessing and analysis scripts were run using MATLAB v.R2024b (ref. ^90^) and EEGLAB v.2024.2.1 (ref. ^91^) with an additional EYE-EEG package^92^.
