# Supplementary material for: Fluctuations in arousal reflect latent state transitions that facilitate behavioural optimization
Source: Nat Hum Behav. 2026 Aug 5;10(9):1790–807. doi: 10.1038/s41562-026-02533-1 (PMC13590411; doi:10.1038/s41562-026-02533-1)
Supplement: Supplementary file 2 [file 41562_2026_2533_MOESM2_ESM.pdf]

nature portfolio

Peer Review File

Fluctuations in arousal reflect latent state transitions for behavioral optimization

Corresponding Author: Professor Matthew Nassar

A version of this paper was originally rejected for publication by Nature Human Behaviour, however that decision was reconsidered after appeal by the authors.

Version 0:

Decision Letter:

9th April 2025

Dear Professor Nassar,

Thank you for submitting your manuscript entitled, "Fluctuations in arousal reflect latent state transitions for behavioral optimization", based on a protocol which was previously considered as a Registered Report, and for your patience during the peer review process.

Your manuscript has now been evaluated by 3 reviewers, which includes both reviewer(s) who originally reviewed the Registered Report as well as new reviewer(s). Their comments are included at the end of this letter. In the light of their advice, I regret that we cannot offer to publish your manuscript in Nature Human Behaviour.

While the reviewers find your work of some interest, they raise concerns about the conceptual advance your findings represent over earlier work, as well as over the design of the experimental procedure (in particular Reviewer #3). We feel that these reservations are sufficiently important as to preclude publication of this work in Nature Human Behaviour.

I am sorry that we cannot be more positive on this occasion but hope that you will find our reviewers' comments helpful when preparing your paper for submission elsewhere.

Sincerely,
[redacted]

[redacted]
Nature Human Behaviour

Reviewers' Comments:

Reviewer #1 (Remarks to the Author):

People often encounter unexpected events that trigger brief activity in the brain's arousal systems but the exact function of such arousal responses has remained unclear. Using measurements of pupil size and EEG during a color prediction task, the authors confirmed five preregistered predictions regarding the relationship between arousal responses and behavioral signs of (biased) perception and learning. They conclude that LC/NE-linked signals changes in the environment to facilitate rapid shifts of mental context. It is commendable that all hypotheses and analyses were pre-registered. The work is timely, of broad interest to anyone researching perception and/or learning. Having said that, I do have several major concerns.

Major:

The task description was unclear. What was the rationale for using two stimuli (left and right), instead of one? On every trial, did subjects always first reproduce/predict one side and then the other, or was this counterbalanced? Did both sequences (left and right) undergo independent state transitions? I think the authors should mention something about the difficulty of this task: reproducing colors is difficult (see estimation error in unstructured block) and simultaneously keeping track of two independent sequences exhibiting multiple change points seems almost too much to ask for.

The basic behavior should be described in much more detail:
- The authors write that reproduction errors were lower in the task than in the unstructured control block (line 188). Why did they use the standard deviation of the estimation error in this analysis, instead of the average absolute estimation error? Is it not a problem that this unstructured (practise) block always came first? Thus, we might be looking at some kind of perceptual learning, rather than the idea that participants incorporated a prior in the main task.
- It would be important to compare the subject's estimation error to that produced by multiple synthetic evidence accumulation approaches including basing the current belief on (i) the last sample, (ii) perfect accumulation, (iii) leaky accumulation, and (iv) the ideal (Bayesian) model. (here, one could apply the empirical estimation error in the unstructured blocks to the synthetic agent's trial-wise belief).
- Would reaction time be an interesting variable in this task?
- Did subjects have a side bias? In other words, do the above suggestions look different for sequences on the left vs right?

The same question about left vs right sequences applies to the main hypotheses. Could authors repeat these main analyses separately per side (e.g., Fig 2C)? Related, does it matter for the results if the authors would take the max bias across both stimuli, instead of the average (e.g., Fig. 3F-H)?

I do very much appreciate that arousal responses increase learning in the change point task, and decrease learning in the oddball context (Fig. 4). However, Fig. 4B,C indicates that subjects learn from extreme subjective prediction errors, even in the oddball contexts. So the question is if subjects really understood the different contexts? Related, what do Fig. 4F-H look like when focusing only relatively large subjective prediction errors?

I have some questions about data normalization. For example, from the Methods it seems that pupil time series were not normalized (to for example units of z-score or percent signal change). I could also not find any mentioning of demeaning or z-scoring predictors in various regression models.
In many figure panels, tickmarkers and labels on the y-axis are missing. See for example Fig. 2A-C, Fig. 2F-I, Fig. 3D, Fig. 3I, etc.

Throughout I think the authors should be more nuanced about the neural correlates of pupil size fluctuations. I appreciate their model-derived hypotheses (focusing on the LC-NA system), but we do know that the pupil is indeed nonspecific (as the authors also write on line 627). I think the authors should at least add a paragraph about this non-specificity and that pupil also tracks ACh, 5HT and orexin.

The Methods seems to be missing a section on Subjects.

Minor:
- The text often cycles back and forth between the paste and present tense.
- Figure panels are often not referenced in order. For example, in the main text, "Figure 2F&G" is referenced before "Figure 2D-E"; note that the authors also use different formatting here (& vs. -).
- "Thus, participants biased their reproductions toward their predictions and reduced this bias in response to surprising stimuli reflecting likely state transitions" (line 288). It would be nice if the authors can test this idea: directly compare bias on trials just before and just after state transitions?
- What do the results in Fig. 3F-G look like for the average absolute bias (across the two stimuli), computed as the perceptual error divided by the objective prediction error (as in Fig. 3A)? If this is different than what is presented, what would this mean?

Reviewer #2 (Remarks to the Author):

In the present study, the authors tested five predictions made by a model of arousal's role in producing latent state transitions. Overall, the predictions were supported by their data. The data came from a color prediction and reproduction task. On each trial, participants were asked to make a prediction about one of two upcoming colored items. Then they would see two colored items, then reproduce the color of one of the two items after a delay. In both critical blocks, the colors of the items were drawn either from a von Mises distribution. Further, trials were occasionally drawn from a uniform distribution. In the changepoint environment, these abrupt discrepant trials signaled a permanent change to a new center point of the von Mises distribution. In the oddball blocks, the discrepant trials lasted only one trial. Arousal signals were drawn from both EEG event-related potentials and pupillary dilations.

Overall, my evaluation of the manuscript was positive. I thought the task had an interesting design with which to ask some interesting questions. I have a few questions I would like the authors to consider.

1) The pupil responds transiently in many different environments, and in addition to being a signal of arousal, it has been used as a signal of cognitive effort, even tasks where there is no "surprise" associated with the task. I was curious about a few things. First, what did the pupillary (and perhaps EEG signals) look like on non-discrepant trials? Do those trials provide

any information about the attentional state people were in? Does that have any bearing on the latent state transition theory?

2) What are some new and untested predictions of the theory that might generate future work? There are a few mentioned in passing in the Discussion, but I think this addition would go a long way in increasing the theoretical advancement of the paper.

3) Perhaps I missed this info, but why did the task have two colored items instead of just one? Was that an important feature of the task? Along those lines, I assume the colors of the items on the two sides of the screen were generated from different distributions. Is that true? If so, doesn't that mean that people are continually updating two models about the colors? Does that fundamentally change the nature of the task, compared to other paradigms where only one source of information is being used?

4) The axis labeling in Figure 2 was odd. There are no units mentioned for many of the y axes. I also don't know what is meant by "intercept" on the y-axes of Figures 2B, 2F, and 2G.

5) Where did the authors file their preregistration? Perhaps I missed that too but I couldn't find a link to that.

6) There are times in the manuscript when the citing is awkward. For example, P. 20 when it's stated, "…the 'network reset hypothesis' was proposed by Bouret and Sara in 2005 (Bouret & Sara, 2005). That is the case for the sentence regarding Yu and Dayan (2005) too.

7) When the authors say they used interpolation for the eye data, what kind did they use? Linear? Cubic spline?

8) It's probably sufficient to say p < .001 instead of using scientific notation for p-values. Using dof for degrees of freedom seemed odd to instead of the traditional notation of t(df) = t-value, p-value, effect size.

9) Was there a specific reason for doing the per-participant regressions rather than a simultaneous analysis like a linear mixed effects model?

Reviewer #2 (Remarks on code availability):

I am not proficient enough with MATLAB to re-conduct the analyses. I did however ensure the link to the code worked and was accessible.

Reviewer #3 (Remarks to the Author):

Li and colleagues study how fluctuations in arousal, as measured by changes in pupil size and the P3a ERP component, associate with a reduction in bias during a color reproduction and color prediction task. Overall the task the authors use is interesting and the question that they address is relevant. The paper is generally well-written, the analyses are overall solid, the figures are good and results interesting. The pre-registration is truly appreciated and a strong aspect of the paper.

I do have some suggestions for improvements and concerns that need to be addressed.

1. Theoretical background: The link between pupil size/P3a and the LC noradrenaline system is too simplisticly presented. Previous (mostly animal) work has shown that pupil size is probably driven by many neuromodulators including dopamine, noradrenaline, serotonin, orexin etc. The paper would benefit from a more nuanced interpretation of the data and more accurate link to theories of neuromodulatory function.

2. Theoretical background/novelty: Overall the paper could benefit from a better embedding in the literature. The link between the pupil/P3 and surprise, prediction errors and learning has been studied for a long time (including modelling) and the authors focus only on a very selective part of that. Especially in experimental psychology many relevant studies have been performed, which should be incorporated, at least to a certain extent. The paper is also framed in a way that the novelty of the results is slightly exaggerated giving a large body of work out there, even when considering that writing for journal such as Nature human behavior may limit the number of references that one may use. Also considering that the reduction in decision bias with increased pupil size has been established for a few years now (see refs below, also cited by the authors), I think the authors can do a better job of highlighting the exact novel aspects of their study. Obviously, the specific experiment that the authors do has not been performed and the direct comparison between the two conditions (changepoint and oddball) is still relevant and interesting, but it needs better embedding and understanding(see also points 4-5).

3.Methods: Regarding the EEG, I'm a bit puzzled by the large baseline differences before stimulus presentation in Fig 2 and worry about data quality, which may be due to the very low trial numbers per block for EEG, but also for pupil analyses (120, meaning there are only a few changepoints). Please specify how many changepoints there were in each condition. Do the authors have an idea why the baseline differences are so large for Pz? The head maps also look fairly noisy given the sample size.

4. Methods: Because the subjects are fully instructed about the task design of the two different blocks it's clear for them that samples that are out of the ordinary (deviant ones) are task relevant in the change point condition and task irrelevant in the oddball condition. In the oddball condition these large deviations can be ignored. Therefore, when a task relevant change is

detected (changepoint condition), and strongly associated with arousal changes due to that, this has a large impact on behavior (reduction in bias), whereas the opposite is true for the oddball condition (you should not attend or take notice of those changes at all in fact, and if you do this is detrimental for behavior). Based on this, can all the results be explained by differential attentional modulations of the pupil/P3a to task relevant versus task irrelevant stimuli? (see also point 5 below). Please discuss, I have added some relevant references below.

5. Interpretation: I think the authors should be a bit more upfront about the fact that the frontal P3a effects were unexpected and that the authors were expecting a parietal P3b effect (Fig 2) in the results section already (as well as abstract). Now the authors address this issue in the discussion section only. The discussion on this issue is also relatively superficial, citing very few studies. The P3a is often associated with attentional orienting and less so with surprise/novelty. Especially given point 4, it may be that the P3a mostly reflects attentional processes triggered by unexpected task-relevant vs irrelevant stimuli in such a perceptual task. At least some further discussion and better embedding in the literature is required here (even better would be analyses that tackle this issue). Also in the abstract the authors refer to "the P300" and the informed reader will directly assume that this relates to the parietal P3b (given the known link to arousal modulations), only later to learn that it's about the P3a.

Minor

-I assume that the intercept plots in Fig 2F/G reflect averaged ERPs, and if so, this should be clarified (for the pupil in 2yB this becomes clear). The reader not familiar with the regression approach may not grasp this directly.

- What is meant by usable data (line 860)? A better rationale for why some subjects were excluded is necessary.

- I often do not fully get the details of the methods or analysis (or only after re-reading parts here and there) and I think some of the methods/analyses require a bit more explanation in the results section for the reader to follow what's going on. In general, the write up of the methods and results could be a bit more intuitive and less technical. I would prefer to have more task description in the introduction/results to better follow the results section.

- I think the authors should be slightly more cautious in the interpretation about "optimization of perception". What the authors show is that larger pupil/P3a responses are associated with a reduced bias, whether perception is optimized is whole different question.

Some relevant refs on pupil/P3/attention/neuromodulation (some are cited by the authors as well)

1. Reimer, J. et al. Pupil fluctuations track rapid changes in adrenergic and cholinergic activity in cortex. Nat Commun 7, 13289 (2016).
2. Alamia, A., VanRullen, R., Pasqualotto, E., Mouraux, A. & Zenon, A. Pupil-linked arousal responds to unconscious surprisal. The Journal of Neuroscience 39, 5369 –5376 (2019).
3. Murphy, P. R., Vandekerckhove, J. & Nieuwenhuis, S. Pupil-Linked Arousal Determines Variability in Perceptual Decision Making. PLoS Comput Biol 10, e1003854 (2014).
4. Leong, Y. C., Dziembaj, R. & D'Esposito, M. Pupil-Linked Arousal Biases Evidence Accumulation Toward Desirable Percepts During Perceptual Decision-Making. Psychol Sci 32, 1494–1509 (2021).
5. Van Kempen, J. et al. Behavioural and neural signatures of perceptual decision-making are modulated by pupil-linked arousal. Elife 8, 1–27 (2019).
6. de Gee, J. W. et al. Pupil-linked phasic arousal predicts a reduction of choice bias across species and decision domains. Elife 9, 1–25 (2020).
7. Zhao, S. et al. Pupil-linked phasic arousal evoked by violation but not emergence of regularity within rapid sound sequences. Nat Commun 10, 1430 (2019).
8. Strauch, C., Wang, C. A., Einhäuser, W., Van der Stigchel, S. & Naber, M. Pupillometry as an integrated readout of distinct attentional networks. Trends in Neurosciences vol. 45 635–647 Preprint at https://doi.org/10.1016/j.tins.2022.05.003 (2022).
9. Murphy, P. R., Robertson, I. H., Balsters, J. H. & O'connell, R. G. Pupillometry and P3 index the locus coeruleus-noradrenergic arousal function in humans. Psychophysiology 48, 1531–1542 (2011).
10. Liao, H.-I., Yoneya, M., Kashino, M. & Furukawa, S. Pupillary dilation response reflects surprising moments in music. J Eye Mov Res (2018) doi:DOI: 10.16910/jemr.11.2.13.
11. LoTemplio, S., Silcox, J., Federmeier, K. D. & Payne, B. R. Inter- and intra-individual coupling between pupillary, electrophysiological, and behavioral responses in a visual oddball task. Psychophysiology 1–14 (2020) doi:10.1111/psyp.13758.
12. Hoeks, B. & Levelt, W. J. M. Pupillary dilation as a measure of attention: a quantitative system analysis. Behavior Research Methods, Instruments, & Computers 25, 16–26 (1993).
13. Liao, H. I., Kidani, S., Yoneya, M., Kashino, M. & Furukawa, S. Correspondences among pupillary dilation response, subjective salience of sounds, and loudness. Psychon Bull Rev (2016) doi:10.3758/s13423-015-0898-0.
14. Vincent, P., Parr, T., Benrimoh, D. & Friston, K. J. With an eye on uncertainty: Modelling pupillary responses to environmental volatility. PLoS Comput Biol 15, e1007126. (2019).
15. Richer, F. & Beatty, J. Contrasting Effects of Response Uncertainty on the Task-Evoked Pupillary Response and Reaction Time. Psychophysiology 24, 258–262 (1987).
16. Friedman, D., Hakerem, G., Sutton, S. & Fleiss, J. L. Effect of stimulus uncertainty on the pupillary dilation response and the vertex evoked potential. Electroencephalogr Clin Neurophysiol (1973) doi:10.1016/0013-4694(73)90065-5.
17. Liao, H. I., Yoneya, M., Kidani, S., Kashino, M. & Furukawa, S. Human pupillary dilation response to deviant auditory stimuli: Effects of stimulus properties and voluntary attention. Front Neurosci (2016) doi:10.3389/fnins.2016.00043.
18. Kloosterman, N. A. et al. Pupil size tracks perceptual content and surprise. European Journal of Neuroscience 41, 1068–1078 (2015).

19. Raisig, S., Welke, T., Hagendorf, H. & van der Meer, E. I spy with my little eye: Detection of temporal violations in event sequences and the pupillary response. International Journal of Psychophysiology 76, 1–8 (2010).
20. Mückschel, M., Chmielewski, W., Ziemssen, T. & Beste, C. The norepinephrine system shows information-content specific properties during cognitive control – Evidence from EEG and pupillary responses. Neuroimage 149, 44–52 (2017).
21. Colizoli, O., de Gee, J. W., Urai, A. E. & Donner, T. H. Task-evoked pupil responses reflect internal belief states. Sci Rep 8, 1–13 (2018).
22. Graves, J. E., Egr, P., Pressnitzer, D. & De Gardelle, V. An implicit representation of stimulus ambiguity in pupil size. Proc Natl Acad Sci U S A doi:10.1073/pnas.2107997118/-/DCSupplemental.
23. Kamp, S. M. & Donchin, E. ERP and pupil responses to deviance in an oddball paradigm. Psychophysiology (2015) doi:10.1111/psyp.12378.
24. Bianco, R., Ptasczynski, L. E. & Omigie, D. Pupil responses to pitch deviants reflect predictability of melodic sequences. Brain Cogn 138, (2020).
25. Filipowicz, A. L. S., Glaze, C. M., Kable, J. W. & Gold, J. Pupil diameter encodes the idiosyncratic, cognitive complexity of belief updating. Elife 9:e57872, (2020).
26. Cazettes, F., Reato, D., Morais, J. P., Renart, A. & Mainen, Z. F. Phasic Activation of Dorsal Raphe Serotonergic Neurons Increases Pupil Size. Current Biology 192–197 (2020) doi:10.1016/j.cub.2020.09.090.
27. Wetzel, N., Buttelmann, D., Schieler, A. & Widmann, A. Infant and adult pupil dilation in response to unexpected sounds. Dev Psychobiol 58, 382–392 (2016).
28. de Gee, J. W., Knapen, T. & Donner, T. H. Decision-related pupil dilation reflects upcoming choice and individual bias. Proc Natl Acad Sci U S A 111, E618-25 (2014).
29. Megemont, M., McBurney-Lin, J. & Yang, H. Pupil diameter is not an accurate realtime readout of locus coeruleus activity. Elife 11, (2022).
30. Schwiedrzik, C. M. & Sudmann, S. S. Pupil diameter tracks statistical structure in the environment to increase visual sensitivity. Journal of Neuroscience 40, (2020).
31. De Gee, J. W., Correa, C. M. C., Weaver, M., Donner, T. H. & Van Gaal, S. Pupil Dilation and the Slow Wave ERP Reflect Surprise about Choice Outcome Resulting from Intrinsic Variability in Decision Confidence. Cerebral Cortex 31, 3565–3578 (2021).
32. Mars, R. B. et al. Trial-by-trial fluctuations in the event-related electroencephalogram reflect dynamic changes in the degree of surprise. J Neurosci 28, 12539–12545 (2008).
33. Nuiten, S. A., de Gee, J. W., Zantvoord, J. B., Fahrenfort, J. J. & van Gaal, S. Catecholaminergic neuromodulation and selective attention jointly shape perceptual decision-making. Elife 12, (2023).
34. DONCHIN, E. et al. Cognition and Event-Related Potentials II. The Orienting Reflex and P300. Ann N Y Acad Sci 425, 39–57 (1984).
35. Wu, Y. & Zhou, X. The P300 and reward valence, magnitude, and expectancy in outcome evaluation. Brain Res 1286, 114–122 (2009).
36. Polich, J. Task difficulty, probability, and inter-stimulus interval as determinants of P300 from auditory stimuli. Electroencephalogr Clin Neurophysiol 68, 311–320 (1987).
37. Braem, S., Coenen, E. & Bombeke, K. Open your eyes for prediction errors. (2015)
38. Kopp, B. & Lange, F. Electrophysiological indicators of surprise and entropy in dynamic task-switching environments. Front Hum Neurosci 7, 1–16 (2013).
39. Harris, D. J. et al. Task-evoked pupillary responses track precision-weighted prediction errors and learning rate during interceptive visuomotor actions. Sci Rep 12, 1–13 (2022).
40. Loughnane, G. M. et al. Catecholamine modulation of evidence accumulation during perceptual decision formation: A randomized trial. J Cogn Neurosci 31, 1044–1053 (2019).
41. Fouragnan, E., Queirazza, F., Retzler, C., Mullinger, K. & Philiastides, M. Spatiotemporal characterization of the neural correlates of outcome valence and surprise during reward learning in humans. Sci Rep (2017) .
42. Gelbard-Sagiv, H., Magidov, E., Sharon, H., Hendler, T. & Nir, Y. Noradrenaline Modulates Visual Perception and Late Visually Evoked Activity. Current Biology 28, 2239-2249.e6 (2018).
43. Dahl, M. J., Mather, M. & Werkle-Bergner, M. Noradrenergic modulation of rhythmic neural activity shapes selective attention. Trends in Cognitive Sciences vol. 26 38–52 (2022).
44. Harris, K. D. & Thiele, A. Cortical state and attention. Nat Rev Neurosci 12, 509–523 (2011).
45. Thiele, A. & Bellgrove, M. A. Neuromodulation of Attention. Neuron vol. 97 769–785 (2018).
46. de Gee, J. W. et al. Dynamic modulation of decision biases by brainstem arousal systems. Elife 6, (2017).
47. Urai, A. E., Braun, A. & Donner, T. H. Pupil-linked arousal is driven by decision uncertainty and alters serial choice bias. Nat Commun 8, 1–11 (2017).

***

**Following suitable revisions, you may want to consider transferring your manuscript. Although we cannot offer to publish your manuscript, I suggest that you consider Scientific Reports as a suitable venue for this work. To transfer your manuscript, please use our manuscript transfer portal. You will not have to re-supply manuscript metadata and files, unless you wish to make modifications. For more information, please see our <a

href="http://www.nature.com/authors/author_resources/transfer_manuscripts.html?
WT.mc_id=EMI_NPG_1511_AUTHORTRANSF&WT.ec_id=AUTHOR">manuscript transfer FAQ page.

Version 1:

Decision Letter:

2nd August 2025

Dear Professor Nassar,

Thank you once again for your correspondence requesting that we reconsider our decision on your manuscript entitled "Fluctuations in arousal reflect latent state transitions for behavioral optimization."

Please accept my sincere apologies for the delay in handling this appeal.

Having considered the points you raised, we would be happy to consider a formal appeal on the decision. To proceed with the appeal, please send us a full point-by-point response to the reviewers, along with a fully revised version of the manuscript.

Once you send me the appeal documents as outlined in your letter, my colleagues and I will send the manuscript back to review.

Please use the link below to submit your revised manuscript and related files:

Link Redacted

Note: This URL links to your confidential home page and associated information about manuscripts you may have submitted, or that you are reviewing for us. If you wish to forward this email to co-authors, please delete the link to your homepage.

We look forward to seeing the revised manuscript and thank you for the opportunity to review your work. Please do not hesitate to contact me if you have any questions or would like to discuss these revisions further.

Sincerely,
[redacted]

[redacted]
Nature Human Behaviour

Version 2:

Decision Letter:

Our ref: NATHUMBEHAV-25010481B

8th April 2026

Dear Dr. Nassar,

Thank you for submitting your revised manuscript "Fluctuations in arousal reflect latent state transitions for behavioral optimization" (NATHUMBEHAV-25010481B). It has now been seen by the original referees and their comments are below.

Please accept my sincere apologies for the delay in sending this decision.

As you can see, the reviewers find that the paper has improved in revision. We will therefore be happy in principle to publish it in Nature Human Behaviour, pending minor revisions to comply with our editorial and formatting guidelines.

We are now performing detailed checks on your paper and will send you a checklist detailing our editorial and formatting requirements within two weeks. Please do not upload the final materials and make any revisions until you receive this additional information from us.

Please do not hesitate to contact me if you have any questions.

Sincerely,
[redacted]

[redacted]
Nature Human Behaviour

Reviewer #1 (Remarks to the Author):

The authors have conducted a comprehensive revision and have satisfactory addressed all of my concerns.

Reviewer #2 (Remarks to the Author):

I appreciate the authors addressing my prior questions and concerns with the manuscript. I have no additional comments/concerns.

Reviewer #2 (Remarks on code availability):

For a MATLAB user (which I am not), the code appears usable and the README sufficiently detailed to execute it.

Reviewer #3 (Remarks to the Author):

The authors have done a good job in responding to the majority of my comments and concerns.

Open Access This Peer Review File is licensed under a Creative Commons Attribution 4.0 International License, which permits use, sharing, adaptation, distribution and reproduction in any medium or format, as long as you give appropriate credit to the original author(s) and the source, provide a link to the Creative Commons license, and indicate if changes were made.
In cases where reviewers are anonymous, credit should be given to 'Anonymous Referee' and the source.
The images or other third party material in this Peer Review File are included in the article's Creative Commons license, unless indicated otherwise in a credit line to the material. If material is not included in the article's Creative Commons license and your intended use is not permitted by statutory regulation or exceeds the permitted use, you will need to obtain permission directly from the copyright holder.
To view a copy of this license, visit https://creativecommons.org/licenses/by/4.0/

We thank the reviewers for their helpful comments. We have addressed each point raised in review below. For ease of reading, we have included our responses in blue and text added to the revised manuscript in *"blue italicized quotations"*

Reviewer #1 (Remarks to the Author):

People often encounter unexpected events that trigger brief activity in the brain's arousal systems but the exact function of such arousal responses has remained unclear. Using measurements of pupil size and EEG during a color prediction task, the authors confirmed five preregistered predictions regarding the relationship between arousal responses and behavioral signs of (biased) perception and learning. They conclude that LC/NE-linked signals changes in the environment to facilitate rapid shifts of mental context. It is commendable that all hypotheses and analyses were pre-registered. The work is timely, of broad interest to anyone researching perception and/or learning. Having said that, I do have several major concerns.

We thank the reviewer for the positive evaluation of our work and have addressed all concerns below point-by-point.

Major:

The task description was unclear. What was the rationale for using two stimuli (left and right), instead of one? On every trial, did subjects always first reproduce/predict one side and then the other, or was this counterbalanced? Did both sequences (left and right) undergo independent state transitions? I think the authors should mention something about the difficulty of this task: reproducing colors is difficult (see estimation error in unstructured block) and simultaneously keeping track of two independent sequences exhibiting multiple change points seems almost too much to ask for.

We apologize for any lack of clarity on our part.

The rationale for two stimuli is that this design feature led to imprecision in perceptual reports that improved our ability to measure biases and reductions therein. In piloting a version of the task with a single stimulus we found that perceptual reports were extremely precise. In-line with normative predictions for such cases, this resulted in extremely small biases, since the perceptual information was far more precise than predictions were. Having two stimuli led to more imprecise color reports, and this imprecision could be reduced by incorporating additional information related to predictions. The higher resulting perceptual bias improved our power to detect the reductions in bias that we hypothesized would occur at state transitions and in response to transient fluctuations in arousal.

In terms of reproductions and predictions, the order across left and right stimuli was randomized across trials resulting in each location being probed first about half of the time.

Left and right stimulus locations underwent completely independent generative processes, such that latent state transitions in one location were independent of those in the other.

We agree with the reviewer that the task has multiple demand elements that could potentially lead to degraded performance. As explained above, degraded color reproduction performance was necessary to achieve reasonable levels of perceptual bias, from which we could measure reductions on state transition trials – and this is why we included two stimuli presented simultaneously. While the structured task included more demands in terms of complexity, it was also easier for participants as measured by color reproduction performance (see Figure S1A). Furthermore, a basic analysis of the overall error distributions showed that the level of improvements in the structured blocks matched those expected from Bayesian integration of sensory information with stored predictions (see Figure S1C). That said, participant predictions themselves were not optimal. They achieved average absolute prediction errors of 39.2°, whereas our normative model made smaller prediction errors (25.3°). This is due in large part to the fact that many participants did not adjust predictions in a context-appropriate manner, as displayed in figure 4B. We note that having some participants who failed to capitalize on the contextual task structure was useful for our design, as it improved our power to detect individual differences in the relationships between arousal, learning, and bias (see Figure 6).

We have now added text to revised manuscript to clarify our motivation for two stimuli (pages 26-27), the randomization of order across sides (page 27), the independence of the generative processes for the two sides (page 27), and to characterize the overall prediction and reproduction performance in our task (page 6).

"The advantage of presenting two simultaneous stimuli was two-fold: first, it allowed us to increase the number of surprising events that would be included in an experimental session, and second it led to reproductions that, while related to the presented color, were somewhat imprecise, meaning that they could be improved through combination of sensory information with prior expectations. Stimuli were prompted in a randomized order."

"…each trial had 2 stimuli that underwent independent generative processes…"

"Participants were generally accurate in their color reproductions but individuals varied in the accuracy of their predictions. The mean absolute color reproduction error across the two prediction blocks was 19.9°, whereas the mean absolute prediction error across both blocks was 39.2°. Predictions were slightly more accurate in the changepoint block (Mean PE = 36.8°) than in the oddball block (Mean PE = 41.7°; $t(62) = 3.73$, $p = 0.0004$, Cohen's d =

0.42). Prediction accuracy differed considerably across individuals both for the changepoint (standard deviation = 9.8°) and oddball (standard deviation = 11.6°) conditions, such that predictions were more reliable indicators of the upcoming color in some individuals than in others"

The basic behavior should be described in much more detail:
- The authors write that reproduction errors were lower in the task than in the unstructured control block (line 188). Why did they use the standard deviation of the estimation error in this analysis, instead of the average absolute estimation error?

We apologize for our lack of clarity here. It is worth mentioning up front that both measures that were used in our previous manuscript (absolute error and standard deviation of the error distribution) tell the same story. Subjects' average absolute estimation errors during the task (mean/SEM = 19.0°/0.85°) were significantly lower than in the practice block (mean/SEM = 21.7°/1.0°; $t = 4.93$, $p < 0.001$). Their estimation errors during the task were also significantly lower than their average prediction errors (mean/SEM = 39.2°/1.2°, $t = 24.3$, $p < 0.001$).

Standard deviation of the error distribution provides a measure of error that is highly correlated with mean absolute errors across participants (In practice block; $R = 0.97$, $p < 0.001$). While the standard deviation of the error distribution may be less intuitive, it has the advantage that its square (error variance, σ^2) can be used to make exact analytical predictions for Bayesian combination of purely perceptual and purely predictive information using Eq. 4 in the manuscript:

$$\sigma^2_{Predicted} = \frac{1}{\frac{1}{\sigma^2_{EstErr}} + \frac{1}{\sigma^2_{PredErr}}}$$

When we quantified the variance of subject color reproductions in the task, we found that it was similar to that predicted by Bayesian combination of subject prediction errors ($\sigma^2_{PredErr}$) and their estimation errors in the randomized practice block (σ^2_{EstErr}); predicted Mean/SEM = 26.6°/0.89°, actual Mean/SEM = 27.0/°1.3°; $t = -0.51$, $p = 0.6$). Thus, we surmised that participants combined these two sources of information at least roughly in accordance with Bayes rule to make their color estimations in the prediction blocks. This account also explains why participants would be biased towards their predictions in these blocks, as a bias toward the predicted value is what allows Bayes rule to achieve improved color estimations.

We have now made our error measures more consistent and clarify why we have opted to use standard deviation for comparing to the theoretical predictions of Bayesian integration (page 33).

"To verify that participants' perceptions were biased in the prediction blocks and that this bias was comparable to what would be expected given Bayesian combinations of priors (predictions) and likelihoods (color presentations), we calculated the theoretical variance that would be expected on perceptual reports that reflected Bayesian integration of prior information (predictions) and likelihood information (unbiased perceptual reports from the unstructured block). To do so we first calculated the variance of participant prediction errors, as a measure of uncertainty on prior probabilities of an upcoming stimulus. Next, we calculated the variance on reproduction errors made during the unstructured practice block as a measure of the likelihood (unbiased percept). We then calculated the variance expected for a weighted combination of these sources of information using the equation below…"

Is it not a problem that this unstructured (practise) block always came first? Thus, we might be looking at some kind of perceptual learning, rather than the idea that participants incorporated a prior in the main task.

The reviewer raises an important point – that participants may have improved their perceptual or memory sensitivity throughout the session – potentially confounding our claim that participants were able to leverage predictions to achieve better color reproductions. In order to examine this possibility, we tested how average reproduction errors changed over the course of the entire session including two initial unstructured blocks followed by the two subsequent structured (changepoint/oddball) blocks. If the improved reproductions in structured sessions were due to some sort of learning process, we should see that errors decrease over the course of structured blocks, with the smallest reproduction errors occurring at the very end of the session.

We have attached a simple figure plotting the average reproduction error against trial number. While perceptual learning could explain the decrease in average reproduction error during the practice block (first 20 trials), it does not explain the steep drop at the start of the prediction block. Note that we omit the practice block from all analyses since the point of this block was to allow participants get used to sequential reproductions that, as the reviewer points how, include a number of control demands. Reproduction errors steadily increase through the 2nd, 3rd, and 4th blocks, indicating that decrease in performance (likely due to fatigue) is greater than any improvement in performance through practice in these blocks. Comparing errors for the earliest/latest trials within each task block and the earliest/latest trials performed in the unstructured block reveals a consistent

performance advantage for the task blocks (See figure below). We also found that there was a significant difference in subject average estimation error from the uninstructed block to the two task blocks (Uninstructed Mean Error/SEM = 21.7°/1.0°; Task Mean Error/SEM = 19.0°/0.9°; $t(62) = 4.93$; $p = 6.44*10^{-6}$), but no difference in average estimation error between the two task blocks (Block 1 Mean Error/SEM = 19.6°/1.3°; Block 2 Mean Error/SEM = 18.5°/0.7°; $t(62) = 0.99$; $p = 0.327$). Thus, we feel confident in saying that reproduction errors decrease from the unstructured to structured blocks as a result of the ability to rely on priors, rather than because of a continuous learning process.

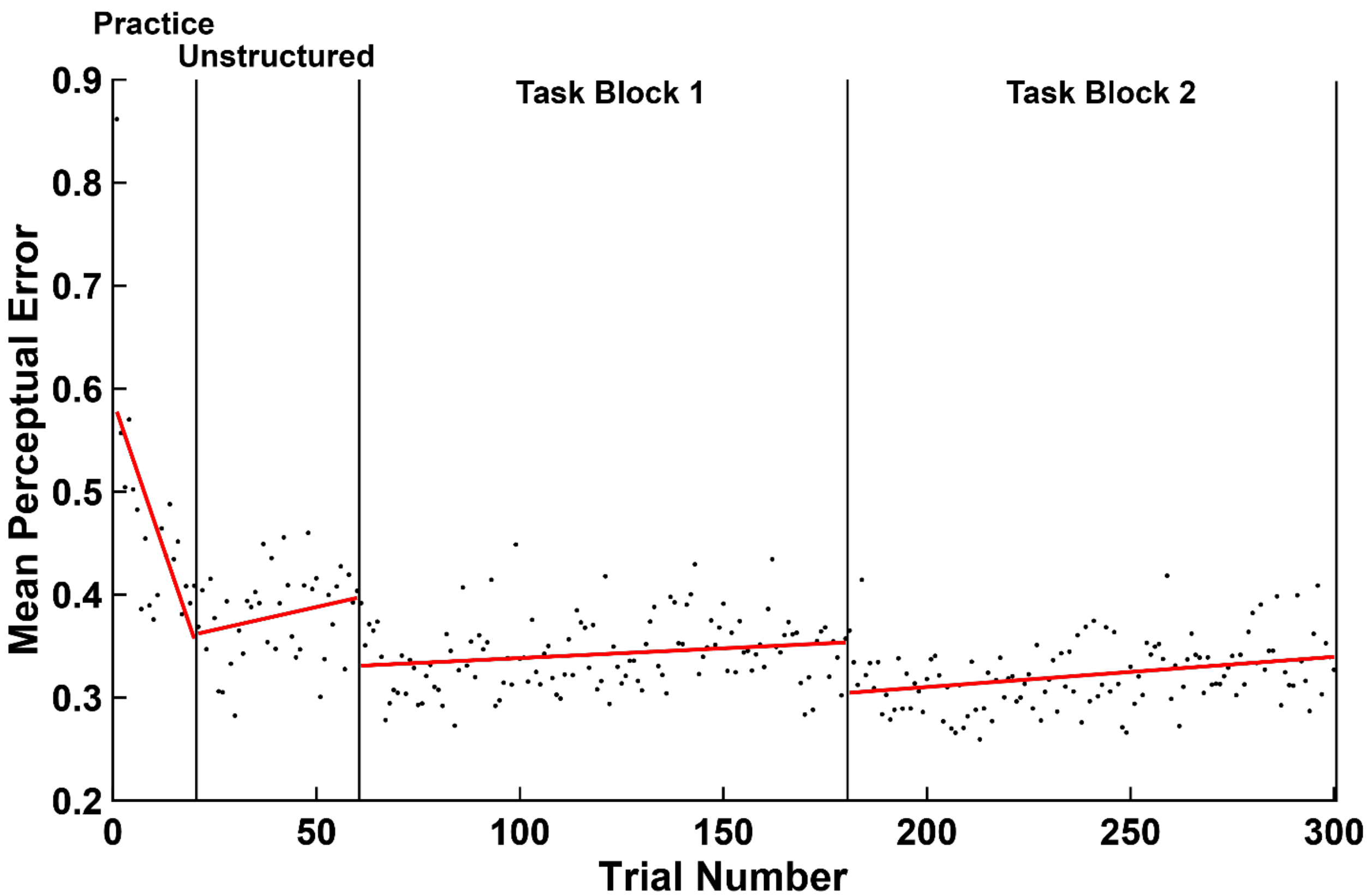

-It would be important to compare the subject's estimation error to that produced by multiple synthetic evidence accumulation approaches including basing the current belief on (i) the last sample, (ii) perfect accumulation, (iii) leaky accumulation, and (iv) the ideal (Bayesian) model. (here, one could apply the empirical estimation error in the unstructured blocks to the synthetic agent's trial-wise belief).

Our previous manuscript demonstrated that state transitions, as identified by a Bayesian model of learning and perceptual inference, provided insight into adjustments in learning and perceptual bias, as well as into its arousal signatures. However, as the reviewer noted, we did not directly compare this model to any other models or evaluate the degree to which

predictions from our model matched actual predictions or reproductions of human participants. We have now done so and report the results of this line of inquiry below.

We first simulated predictions from four heuristic models and compared them to our Bayesian model in terms of their ability to match predictions made by human participants. First, we considered a perfect accumulator, that makes a prediction that reflects the circular mean of all colors observed to this point in the block (Perfect Accum). We also considered a model that resets the accumulation process at state transitions (Accum), such that it predicts the upcoming color according to the mean of all color perceptions since the last changepoint/oddball. We note that instantiating this model would still require recognizing state transitions and resetting the accumulator at these events – however we consider it a more viable candidate than a model without such resets. We also considered a leaky accumulation model (Leaky), which predicts the upcoming color as a normalized geometrically weighted mean of color perceptions from previous trials. We fit the leak parameter at the group level and found the best performance when past observations were discounted by a decay rate of 0.3. Finally, we also considered a very simple model that makes predictions about the upcoming color completely based on its percept of the last observed color. For all of our models, percepts from previous trials were set to be the actual color of the presented stimulus plus mean zero Gaussian perceptual noise with standard deviation equal to the mean standard deviation of reproduction errors, across participants, in the unstructured block. In order to minimize the degree to which the exact pattern of perceptual noise affected fit quality, we simulated 1000 repetitions of each agent's behavior and averaged the color predictions across these repetitions. We note that, for the heuristic models, the expectation of this procedure would be equivalent to computing predictions in the absence of perceptual noise, however since this is not the case for our Bayesian model, we have performed the exact same procedure for all models to put all models on equal footing for comparison.

We evaluated the degree to which each model could explain participant predictions by running five separate linear regressions that explained the participant predictions in terms of those made by each heuristic model. Since data are circular, this regression included a circular (Von Mises) error distribution, with the concentration (1/variance) of the residual error distribution providing a measure of goodness of fit. Model fits (as defined by the concentration of residual errors) for the Bayesian model and the leaky accumulation model were very clearly better than the perfect accumulation models with and without knowledge of state transitions and the perceptual model (see left figure below; bars and error bars represent mean and 95% confidence interval across participants respectively). While the Bayesian and leaky accumulator models were similar in their ability to fit participant prediction behavior, the Bayesian model performs significantly better than the leaky

accumulation model in a statistical comparison (Bayes-Leaky; $t(62) = 3.50$; $p = 0.001$; Cohen's $d = 0.44$). This difference is especially pronounced in subjects with higher model concentrations, which tend to be those with more accurate predictions (see right panel below).

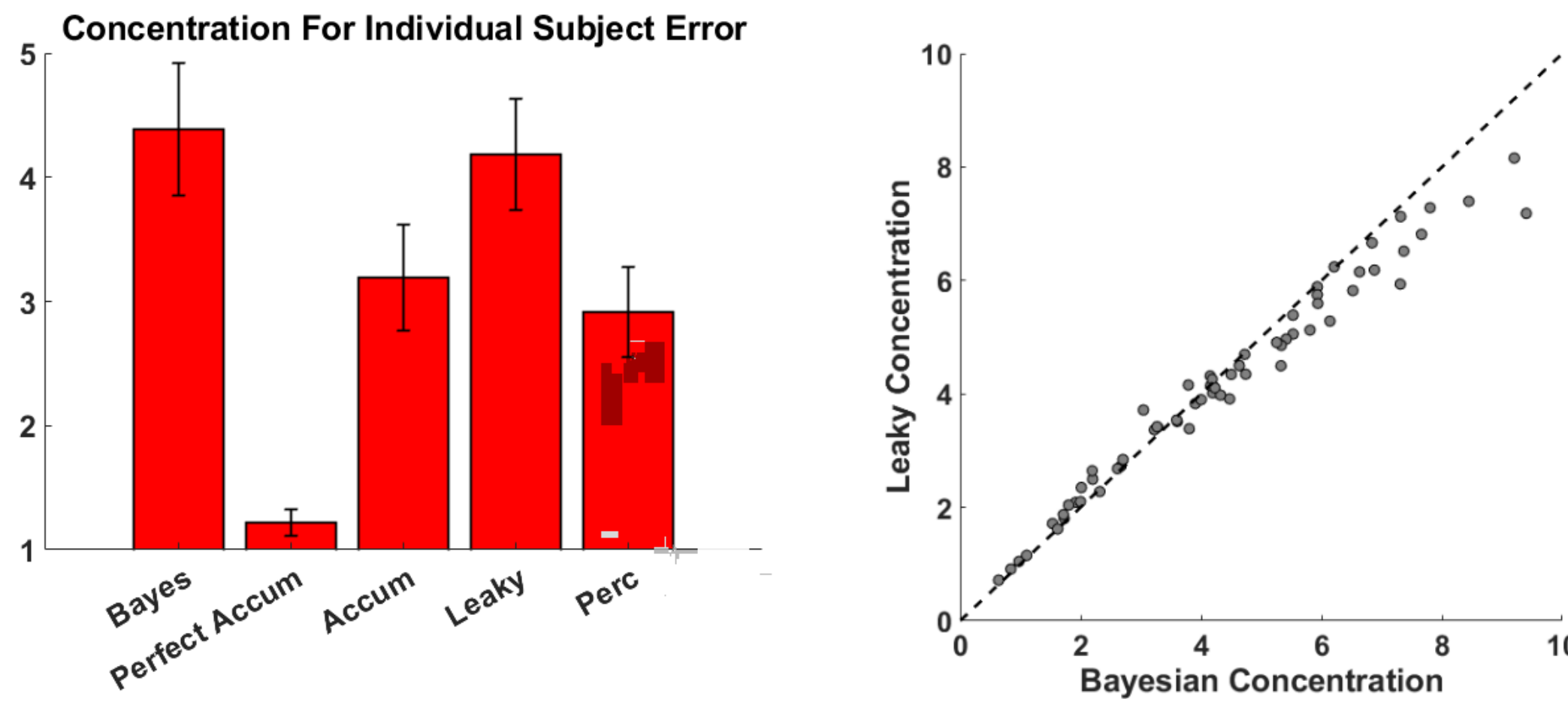

We also compared our model's ability to explain bias in perceptual reports to that of the best heuristic model (leaky accumulator). In this case, we asked to what degree the errors predicted by each model correlated with the reproduction errors produced by participants. Our Bayesian model dynamically adjusts its bias according to the inferred state transition probability, and thus we considered its contributions to explain bias in two ways; first, we quantified the degree to which human reproduction errors are biased toward a-priori predictions of the Bayesian model (Bayesian Prediction), next, we quantified the degree to which the same errors are biased toward a-posteriori perceptual inferences of the Bayesian model (Bayesian Perception). For the leaky integrator model, we could only quantify the degree to which its integrated average of previous observations explained biases in reproduction errors (Leaky). In all cases, we correlated model error (averaged across 1000 simulations) with human subject reproduction error across trials. Note that such correlations should be small, since the vast majority of variability in the reproduction errors is, by design, due to uncertainty about the exact color, as for all delayed reproduction tasks. Nonetheless, correlations tended to be positive for all models (See histogram of correlations across participants below). On average, the Bayesian perception provided a better correlation with the subject error (Mean $r = 0.049$) than both the Bayesian prediction (Mean $r = 0.036$; R Bayes Percept-R Bayes Pred; $t(62) = 2.12$, $p = 0.0381$) and the leaky accumulator (Mean $r = 0.026$; R Bayes Percept-R Leaky; $t(62) = 3.60$, $p < 0.001$; see right panel below for per-subject comparison of the two models). The Bayesian model's prediction error was also more strongly correlated with subject error than the leaky accumulator's (R Bayes Pred-R Leaky; $t(62) = 2.07$, $p = 0.0429$).

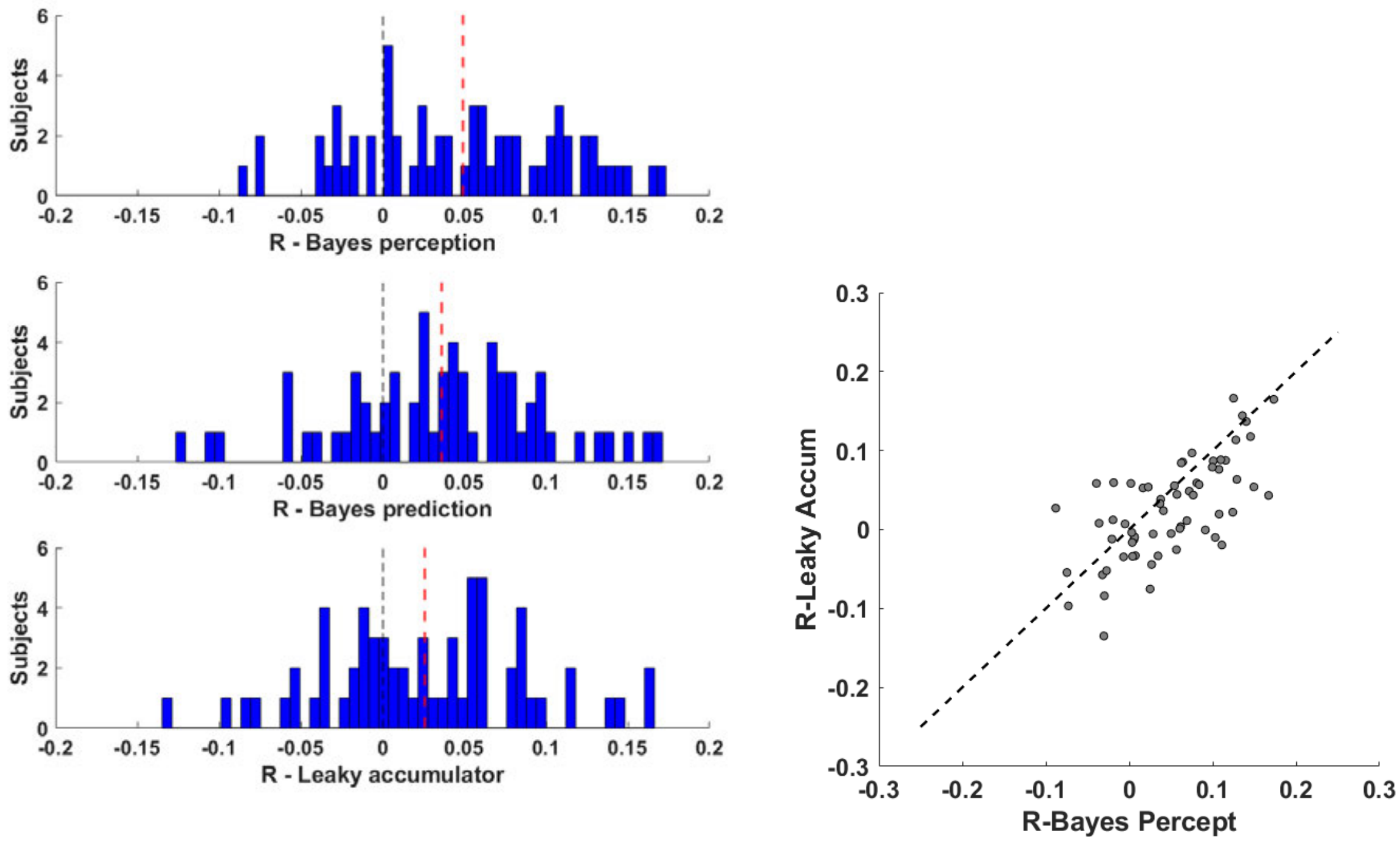

Taken together with the model comparison for predictions above, these results support our claim that the Bayesian model provides a better description of behavior than that achieved by simple heuristics. An overview of the methods and results presented here has been added to the manuscript and described in Supplementary Figure 3 (page 47).

- Would reaction time be an interesting variable in this task?

We have done some analyses on reaction time, but the continuous nature of color selection in the task makes it difficult to examine, since reaction time is closely correlated to how close the starting color on the prompt (randomized uniformly) was to the color participants selected ($r = 0.30$). We have tried to investigate how initiation reaction time, measured by the time taken by the participant to begin moving the mouse, relates to surprise in the task, but found no significant differences between reaction times on surprising and typical trials in the changepoint (mean surprise RT = 3.16 s; mean typical RT = 3.23 s; $t(62) = -1.85$, $p = 0.069$), oddball (mean surprise RT = 3.14 s; mean typical RT = 3.15 s; $t(62) = 0.08$, $p = 0.93$), or both blocks combined (mean surprise RT = 3.16 s; mean typical RT = 3.19 s; $t(62) = -0.97$, $p = 0.34$). For these analyses, we removed trials where the participant timed out or when reaction time was less than 50 ms. Thus, while we share the reviewer's belief that one would expect processing time to differ across conditions that involve versus do not involve a latent state transition, unfortunately our task was not well posed to measure these differences

- Did subjects have a side bias? In other words, do the above suggestions look different for sequences on the left vs right?

There was no difference in reproduction errors on the left and right sides (Left Error Mean/SEM = 19.8°/0.89°; Right Error Mean/SEM = 20.0°/0.86°; $t(62) = 0.66$; $p = 0.51$).

For prediction errors, there was a slightly higher average prediction error for the right stimulus location for low state transition probability trials (Left Error Mean/SEM = 33.0°/1.3°; Right Error Mean/SEM = 34.2°/1.4°; $t(62) = 2.65$, $p = 0.01$). This difference is numerically small, however, and seems to be driven by the participants with the worst performance (highest absolute prediction errors (see figure below).

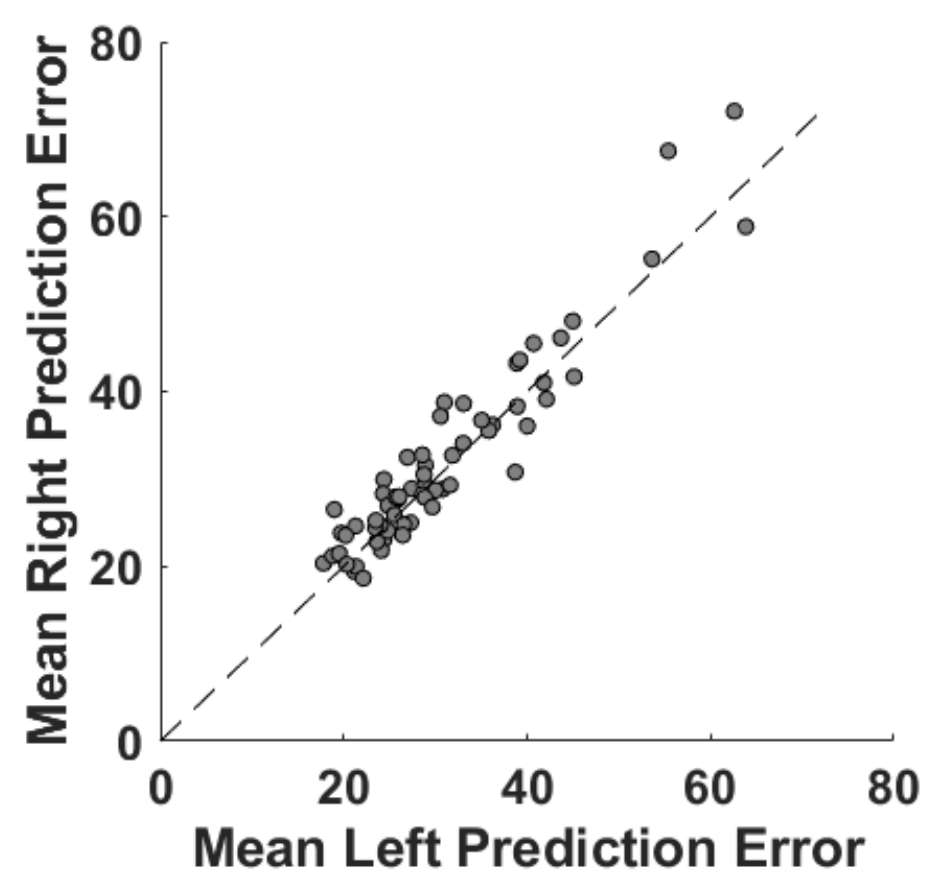

Average subject level bias rates did not differ across left and right stimuli (Left Bias Mean/SEM = 0.352/0.006; Right Bias Mean/SEM = 0.349/0.005; $t(62) = 0.63$, $p = 0.53$). Subject level learning rates also did not differ across left and right stimuli (Left Learning Rate Mean/SEM = 0.568/0.006; Right Learning Rate Mean/SEM = 0.560/0.006; $t(62) = 1.73$, $p = 0.09$).

The same question about left vs right sequences applies to the main hypotheses. Could authors repeat these main analyses separately per side (e.g., Fig 2C)?

We understand that the reviewer is interested in understanding whether our primary results might be specific to one stimulus location or the other. Rather than doing our primary analyses separately for each side, which would cut the number of state transitions in half thereby reducing our power considerably, we have instead included additional interaction regressors in our model to evaluate whether learning or perceptual bias were measurably different across the left versus right stimulus locations.

There does not seem to be any effect of stimulus side on learning or bias behavior. The plot below shows the individual subject variables for PE * Side regressors in our estimation error

and prediction update models. Neither of the means of these regressors differ significantly from zero (Estimation Error Coefficient, t(62) = 0.08, p = 0.94; Prediction Update Coefficient, t(62) = 1.10, p = 0.27). While some subjects may individually be more likely to be more biased or learn more from changes on one side over the other, there is no consistent group level side bias.

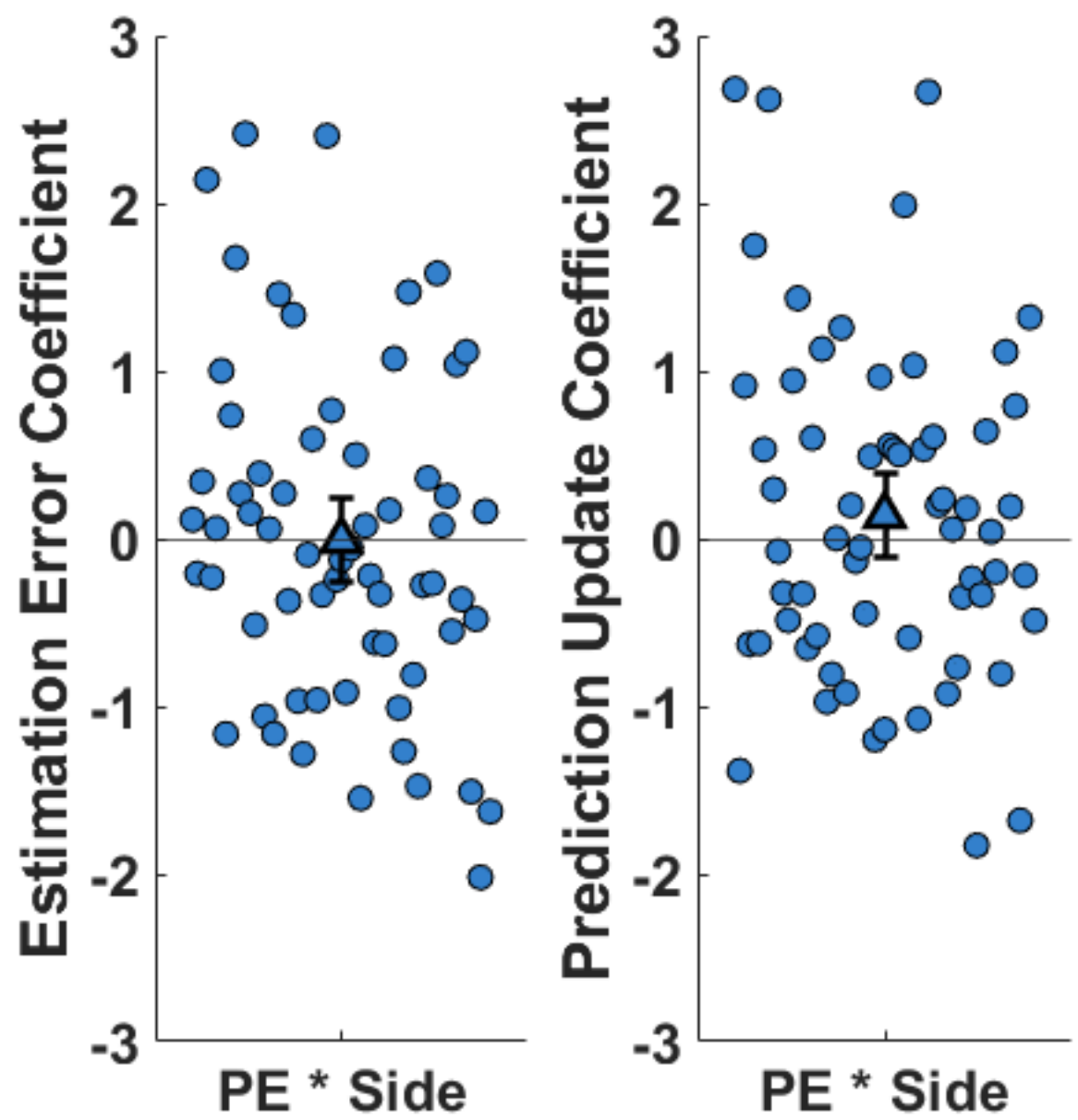

There also does not appear to be a significant difference in the pupil response when looking separately at surprise on the left vs right stimuli (see figure below). There is a trend toward participants responding slightly earlier for the right stimulus, but these differences are small. Below on the left is plotted the coefficients of a running pupil regression where left STP and right STP were treated as separate explanatory variables. The right plot shows the coefficient of a running pupil regression where instead of including STP an explanatory variable, we instead included the difference between left and right STP (shaded areas show standard error of mean, no points where $p<0.05$).

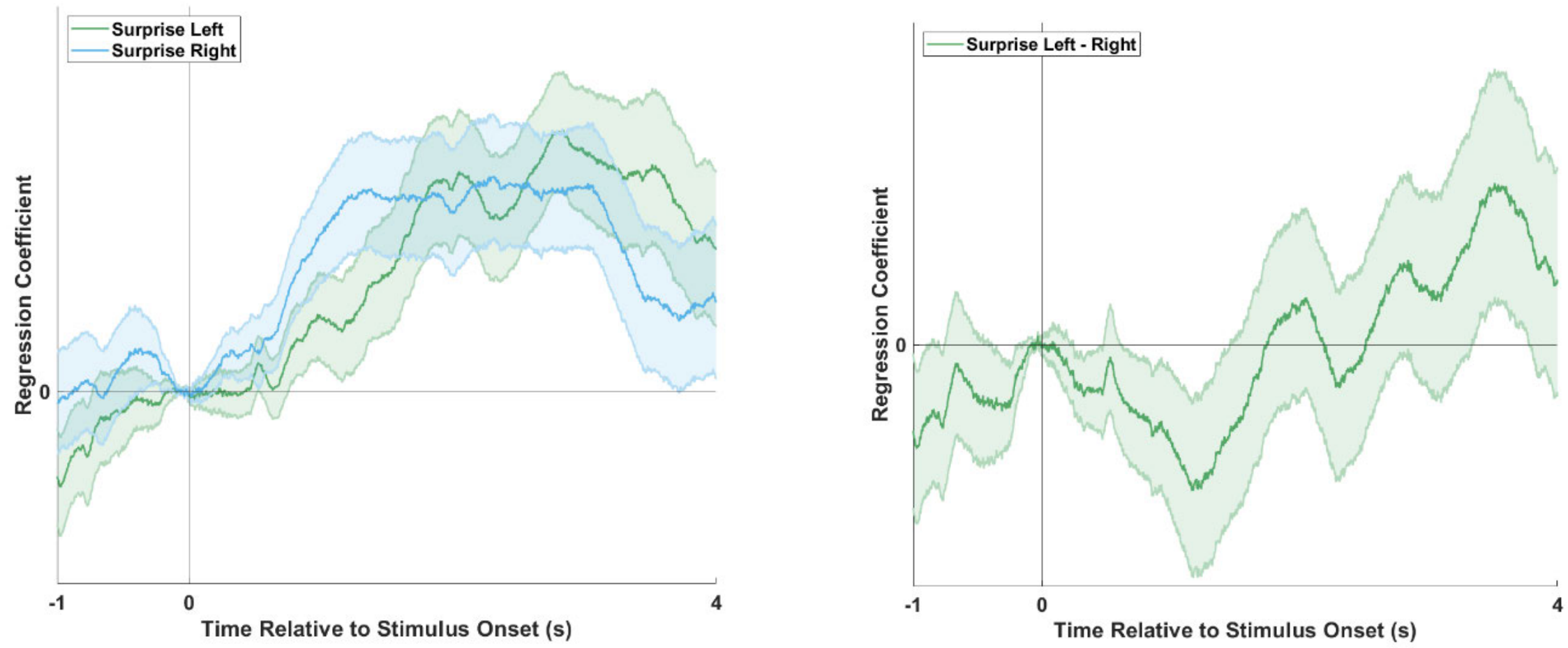

With respect to EEG, we did find a side specific effect of surprise, however it was not related to learning or bias. While both the left and right stimuli induce the P3a signal increase around 450 ms (see top left and top middle panels of figure below), there is a slight side difference that peaks around 500-600 ms post-stimulus where state transitions on the left induce a more positive signal in the left parietal region and state transitions on the right induce a more positive signal in the right frontotemporal region. In short, it seems that there is some laterality here, where state transitions induce weak positive signals on the same side as where the stimulus was presented. When we perform a regression including a term for the difference between left STP and right STP, we see that there is a significant ERP cluster around 400-700 ms post stimulus in the right frontotemporal region ($p = 0.0040$). This ERP was not related to learning or bias in either block, as was verified by performing the analyses used to generate figures 3F and 4F in the main paper and finding that the learning did not differ across blocks in its relationship to the cluster (Slope difference, $t(56) = 1.03$, $p = 0.31$) and bias does not reduce with increased cluster strength (Slope sum, $t(56) = 1.77$, $p = 0.08$). Thus, while there is some lateralization of the state transition representation in our EEG data, it does not interact with the main effects that we report, which are common to state transitions in both target locations.

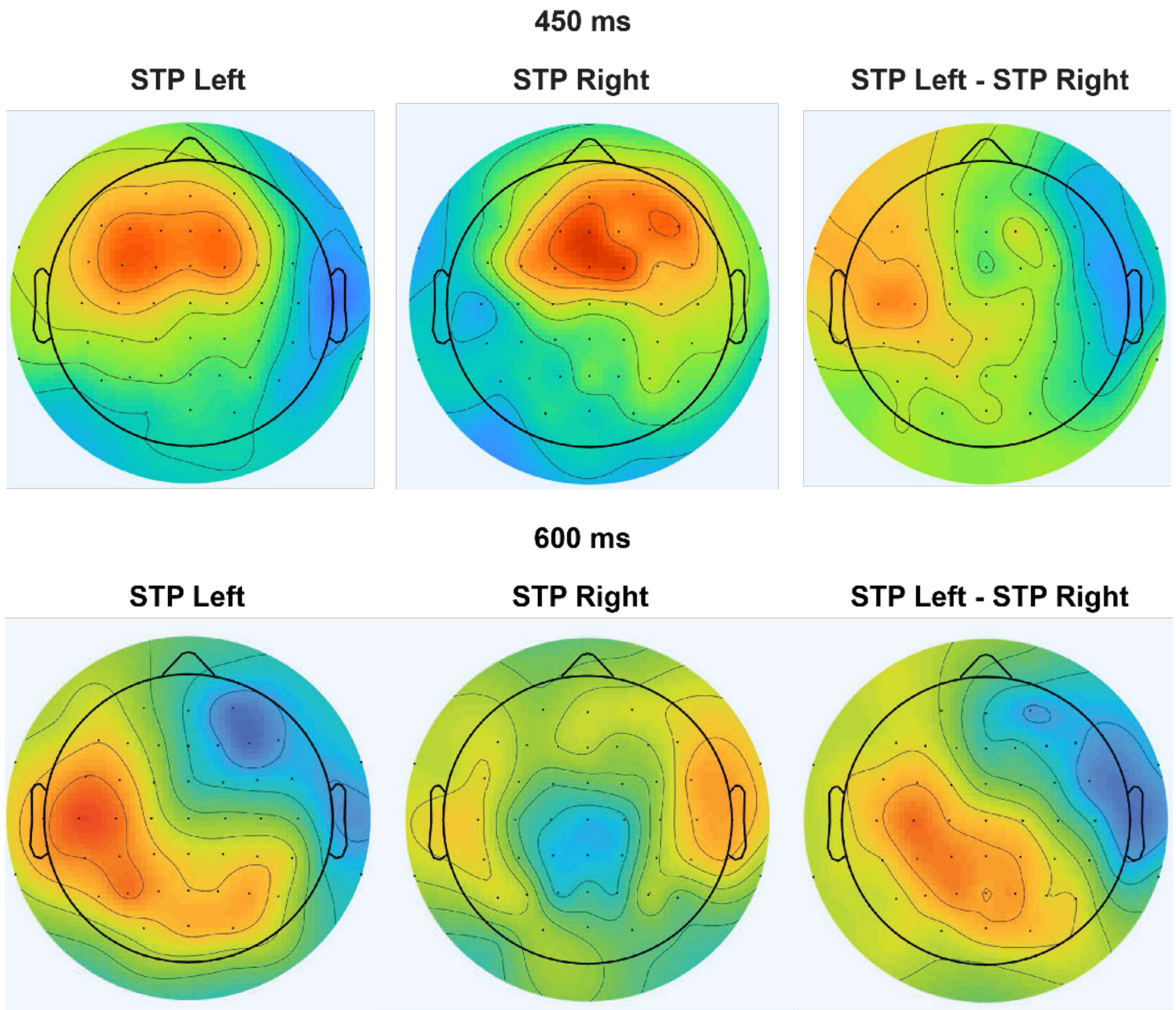

Related, does it matter for the results if the authors would take the max bias across both stimuli, instead of the average (e.g., Fig. 3F-H)?

The general pattern of EEG and pupil results looks similar when “maximum bias” is used in place of the “mean bias” reported in our manuscript, albeit with some minor discrepancies. For example, the effect of “max bias” on arousal appears to be slightly stronger in the oddball block than the changepoint block, especially when aggregating across pupil and EEG data (see figure below). Whenever there are two possible ways to perform an analysis that yield slightly different (even if not significantly different) results, it is worthwhile to consider justification for using one method rather than the other.

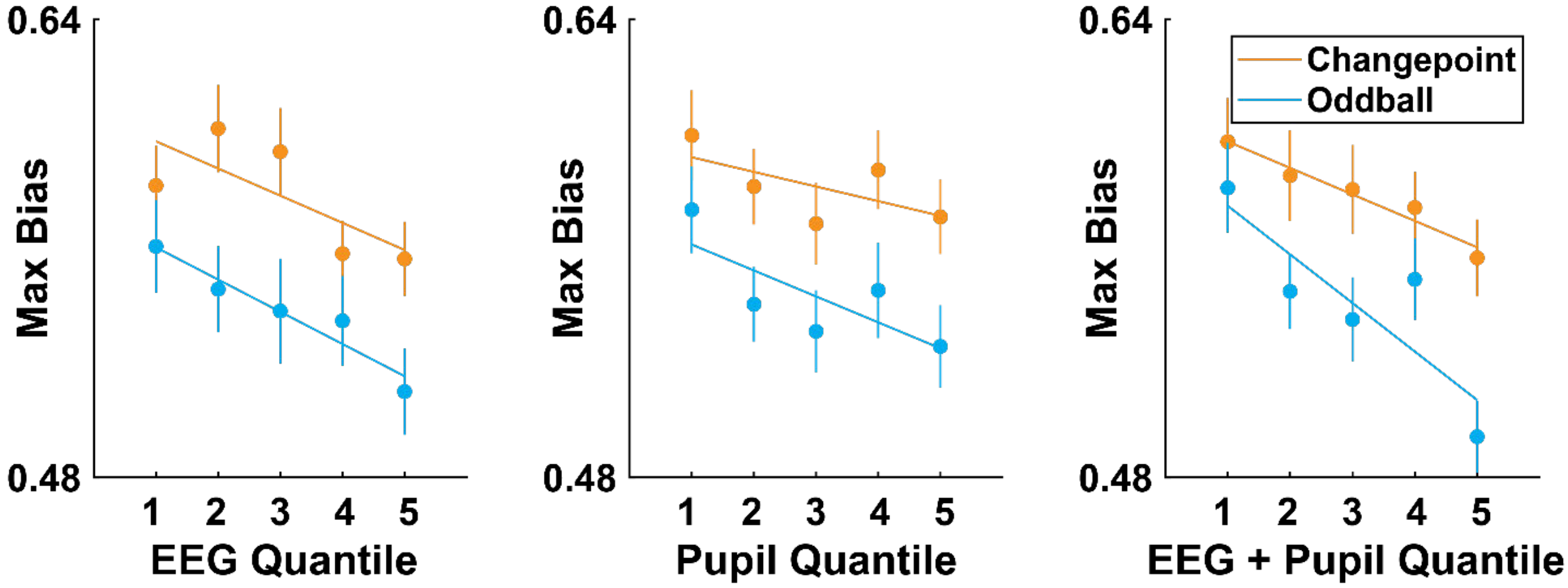

In this case, the reason we used “mean bias” as our behavioral measure was that we performed a series of simulation experiments in the Bayesian model and found that “mean bias” estimated from simulated behavior had a stronger relationship to the underlying state transition probabilities than did any other measure. In particular, model state transition probability was more correlated with mean bias than other measures of bias, including maximum bias, which has the shortcoming that it often results in bias measures of exactly one when bias measures above one are reset to one (which also turns out to improve robustness of relationships to underlying latent state probability in our modeling). On low prediction error trials, small changes in reproductions have large impacts on bias, and taking the bias value for only one of the two stimuli instead of aggregating across both, decreases the sensitivity of the bias measure. Considering that both our mean bias measure and the max bias measure proposed by the reviewer produce similar results, and that we have more confidence that mean bias relates to the underlying latent state probabilities that motivated our work, we have maintained our analyses in terms of mean bias in the revised manuscript.

I do very much appreciate that arousal responses increase learning in the change point task, and decrease learning in the oddball context (Fig. 4). However, Fig. 4B,C indicates that subjects learn from extreme subjective prediction errors, even in the oddball contexts. So the question is if subjects really understood the different contexts?

We agree with the reviewer that on average, participants still made updates on oddball events as shown in Figure 4B-C of our previous manuscript. It is noteworthy that these updates were proportionally smaller than those made in the changepoint condition for the same error magnitudes (compare blue to orange) and those made for moderate errors in the oddball condition (compare slope of inlying to outlying blue datapoints in Figure 4C). Thus, across all participants, it is clear that participants are not applying a fixed proportional bias and instead reducing this bias for large errors in the oddball condition.

However, in line with the reviewer's point, not all participants substantially reduced their proportional bias in response to oddball events. One way look at this is through the update behavioral regression coefficients for the term that measures the degree to which updates correspond to prediction errors more on changepoint than oddball trials (STP*PE*Condition). Note that these coefficients, which are shown in Figure 4D of our manuscript, are bimodally distributed, with one cluster of participants near zero, and another cluster of participants taking more positive coefficients.

We take these two clusters of participants to mean that some subjects were able to correctly utilize the differential task structures to improve predictions (positive cluster), whereas others were not (cluster near zero). We see this variability as a strength, since it can allow us to better link the state transition signals we measure to their functional consequences. In particular, the reviewer may predict that the individuals who show near zero STP*PE*Condition coefficients may have arousal signatures that are less bidirectionally linked to learning – and indeed this appears to be the case.

Below, we provide alternate versions of Figure 4F-H where the analyses are applied separately to the cluster of participants with positive coefficients (top; n(EEG) = 35; n(pupil) = 37) and the cluster of participants with near-zero coefficients (bottom; n(EEG) = 22; n(pupil) = 23). Note that the relationships between learning and EEG/pupil signal are overall stronger in subjects with higher STP * PE * condition update coefficients than in subjects with near-zero coefficients. To show this, we partitioned participant data using a density-based spatial clustering approach. For participants who showed clear behavioral evidence of context-dependent learning rate adjustments (high STP * PE * condition coefficients), stronger physiological indicators of state transitions corresponded to higher learning in the changepoint condition in lower learning in the oddball condition (top panels). However, this trend is far less pronounced for participants who did not show clear behavioral evidence of adjusting to the changepoint and oddball contexts (bottom). Indeed, if we perform statistics on these two groups separately, we see that the results we report in our manuscript are only statistically significant in the group that showed evidence of context-dependent updating. Specifically, when EEG and pupil data are combined, this group had a significant difference in the slope of the relationship between learning and arousal signals between the changepoint and oddball blocks EEG ($t(34) = 4.32$, $p = 0.0001$, Cohen's $d = 0.73$), pupil ($t(35) = 3.37$, $p = 0.0018$, Cohen's $d = 0.56$), and combined pupil and EEG data ($t(34) = 3.95$, $p = 0.0002$, Cohen's $d = 0.67$). However, this relationship did not hold up in subjects with smaller differences in learning behavior between the two blocks in EEG ($t(21) = 0.91$, $p = 0.37$, Cohen's $d = 0.19$), pupil ($t(23) = 1.50$, $p = 0.15$, Cohen's $d = 0.31$), or combined pupil and EEG data ($t(20) = 1.81$, $p = 0.076$, Cohen's $d = 0.39$).

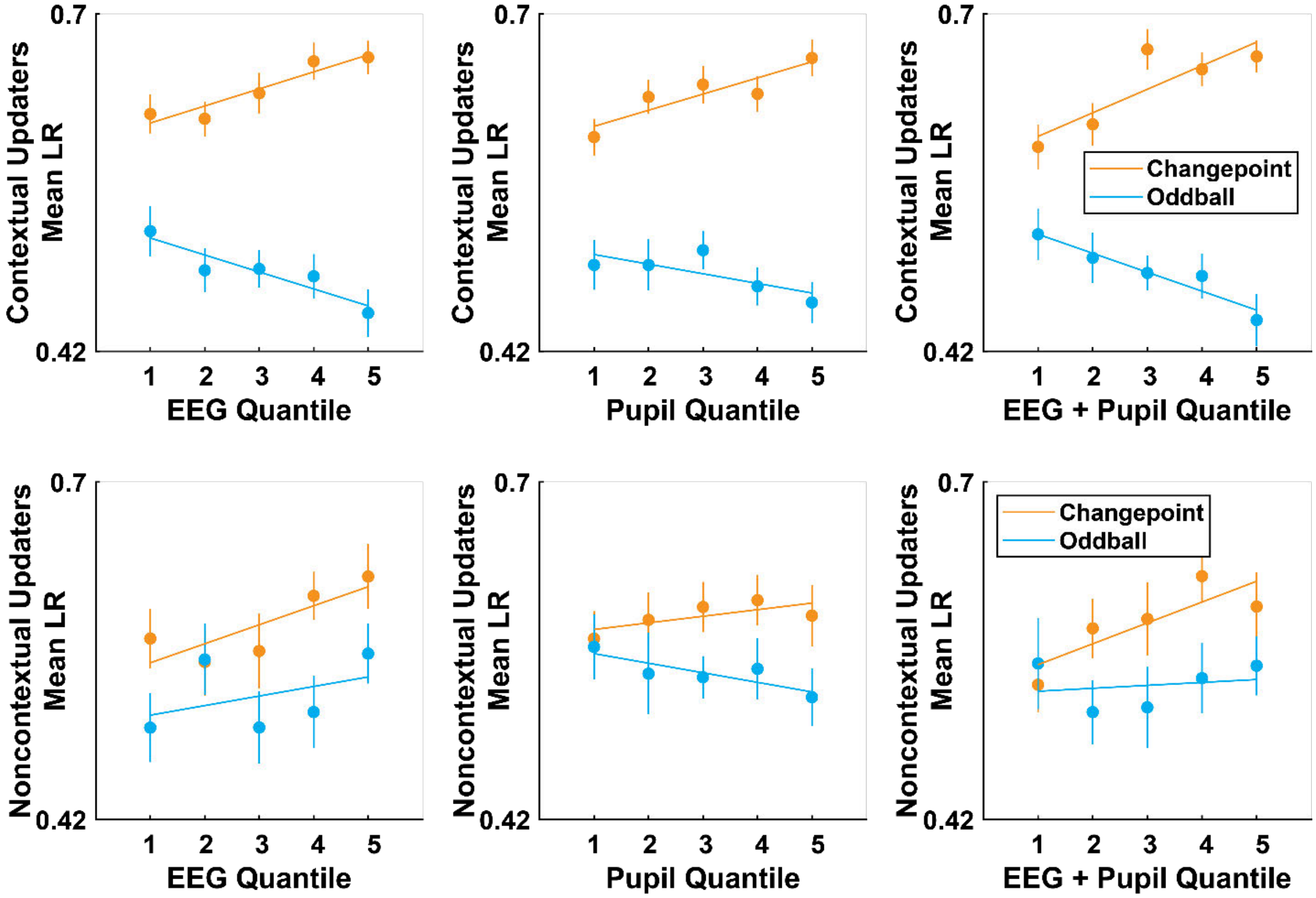

We interpret this as suggesting that the relationship between arousal signals and behavior is highly contingent on an individual's understanding of how states transition to one another (i.e. changepoint or oddball), which would be another direct prediction of our latent state theory, albeit one that we did not include in our original preregistration. We now include a description and discussion of these results in our manuscript (pages 15 & 21-22) and have added the above figure as Supplementary Figure 6.

*"[Learning-arousal] relationships were primarily driven by the participants who modulated learning behavior most dramatically across the conditions as measured by sPE * STP * Condition coefficients in the behavioral regression model (Figure S6)."*

*"Our results also highlight strong relationships between task behavior and the strength of the arousal relationships. Although optimal task performance required adjusting learning across changepoint and oddball blocks many participants behaved similarly across these two conditions (note variability in PE*STP*Condition coefficients in Figure 4D), failing to dynamically adjust learning behavior appropriately. Arousal signatures tracked this behavioral heterogeneity, relating bidirectionally to learning only in those individuals who adjusted learning behavior appropriately across conditions (Figure S6), These same individual differences in behavior contributed to the variability that allowed us to*

demonstrate that the strength of bias-arousal relationships was related to learning-arousal relationships, supporting a common latent states transition mechanism (Figure 6). Thus, while behavioral data highlight that not all participants took full advantage of the distinct temporal structures we employed, our pupil and eye-tracking data demonstrate that when they did, arousal could be used to reliably anticipate their degree of bias and learning."

Related, what do Fig. 4F-H look like when focusing only relatively large subjective prediction errors?

Based on our theoretical framework, we would predict that performing the analyses that generated figures 4F-H using only extremely large errors would yield far less pronounced relationships between arousal signals and learning behavior, since all of these trials would be subjectively perceived as state transitions. This would, in our framework lead to universally high learning rates in the changepoint block and universally low learning rates in the oddball block. However, if all trials analyzed are state transitions, then the arousal signatures, which we propose reflect noisy reflections of subjective internal state transition signals, should not tell us much about behavior that we do not already know based on the particulars of the trial.

We see results very consistent with this idea when we performed the same analyses on trials with large subjective prediction errors. We repeated the analyses used to generate figures 4F-H, while only selecting out trials where at least one stimulus of the trial had >60 degrees of subjective prediction error. Below we see the results are much like what we expected. Note that overall learning rates in these trials are very high in the changepoint condition and very low in the oddball condition. That said, there is a slight trend toward the bidirectional learning effect we originally observed. However, these relationships are not as strong as they were in the original analyses, for example, the learning/arousal slope difference across blocks in aggregated pupil and EEG data ($t(55) = 1.93$; $p = 0.059$; Cohen's $d = 0.26$). There are slight slopes in the direction of increasing learning in relation to arousal in the changepoint block and the opposite in the oddball block.

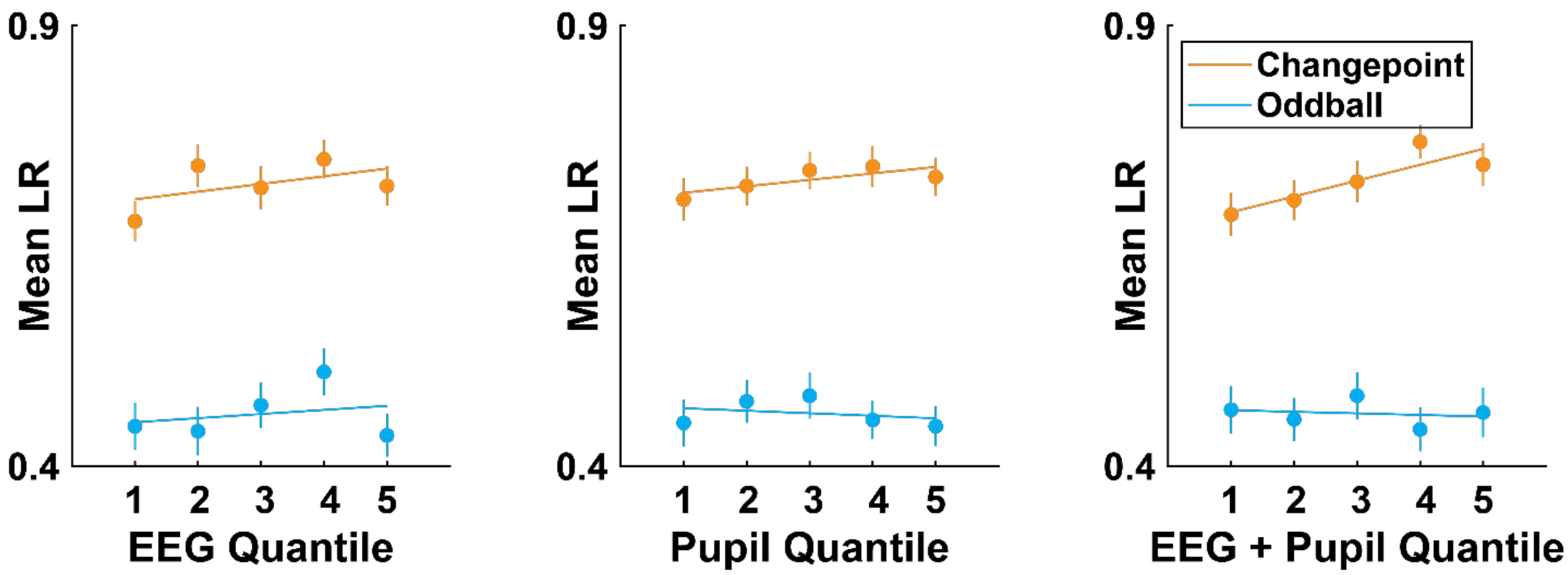

I have some questions about data normalization. For example, from the Methods it seems that pupil time series were not normalized (to for example units of z-score or percent signal change). I could also not find any mentioning of demeaning or z-scoring predictors in various regression models.

Our apologies for not including these details in the original manuscript.

For pupil regressions, we z-scored pupil area for each subject across all timepoints and trials. Note that, since regressions were run separately for each timepoint, the dependent variable itself in these regressions was neither normalized nor mean centered, allowing the intercept term in these regressions to capture dilations across timepoints. All other predictors in the pupil regression model were z-scored, to aid interpretability of coefficients and their magnitudes. Thus, all coefficients in the pupil regression reflect a change in standard units of pupil diameter conferred by a standard unit of the predictor variable.

For EEG regressions, we maintained the original measurement units of microvolts for our dependent variable. Again, for EEG regressions, we z-scored all predictor variables other than the intercept term to aid with interpretability. Thus, all coefficients in the EEG regression reflect a change in microvolts conferred by a standard unit of the predictor variable. In response to this question, we also ran the EEG regressions by z-scoring each channel across all timepoints and trials similarly to how we handled the pupil data, and we verified that this normalization decision does not impact our results.

We now provide an explicit explanation of our normalization in the methods as follows (page 34):

"All regressors and pupil data were mean centered and normalized before regression. EEG voltages were not normalized in order to retain our original units of millivolts."

Additionally all units for regression coefficients are now specified in figure captions.

In many figure panels, tickmarkers and labels on the y-axis are missing. See for example Fig. 2A-C, Fig. 2F-I, Fig. 3D, Fig. 3I, etc.

We have now added them.

Throughout I think the authors should be more nuanced about the neural correlates of pupil size fluctuations. I appreciate their model-derived hypotheses (focusing on the LC-NA system), but we do know that the pupil is indeed nonspecific (as the authors also write on line 627). I think the authors should at least add a paragraph about this non-specificity and that pupil also tracks ACh, 5HT and orexin.

We completely agree with the reviewer that this is an important aspect to note when interpreting the results from our pupil data. We have since included a paragraph in the discussion (page 23) of the multiple neural correlates and causal contributors to pupil dilation and how it relates to the project. We have reproduced this paragraph below for ease of review:

"While our study relied on peripheral markers of arousal—specifically EEG and pupillary responses to latent state transitions—our results have implications for the systems that underlie them. In particular, our theoretical framework is grounded in well-established physiology, pharmacology, and anatomy of the LC/NE system. LC neurons respond robustly to surprising stimuli irrespective of modality and project broadly to cortical and subcortical regions, where they release norepinephrine (NE) (Foote et al., 1980; Aston-Jones & Cohen, 2005). In these target regions, NE has been shown to amplify feedforward processing (Devilbiss & Waterhouse, 2011; Rodenkirch et al., 2019) and suppress local recurrent activity (Cools & Arnsten, 2022; Hasselmo et al., 1997), thereby providing a mechanism by which active latent state representations might be "reset" in response to new inputs (Nassar, 2024). Moreover, activation or inactivation of the LC/NE system facilitates or impairs behavioral flexibility (Donchin & Coles, 1988a; Jepma et al., 2016), suggesting its involvement in adaptive state updating. Although we did not directly measure LC activity, we used pupil area and the P300 component of the EEG signal as indirect markers—both of which have been proposed as proxies for LC/NE system activation (Joshi et al., 2016; Joshi & Gold, 2021; Murphy et al., 2011; Nieuwenhuis et al., 2005; Vazey et al., 2018).

However, these proxies are inherently nonspecific. For example, pupil dilation in our task also likely corresponds to activation of multiple neuromodulatory systems—including acetylcholine (ACh), serotonin (5HT), and orexin—which each play distinct roles in modulating brain state and behavior (Cazettes et al., 2020; Grujic et al., 2023, 2024; Joshi & Gold, 2021 ; Maheu et al., 2025; Reimer et al., 2016). Thus, while our study was motivated to test a theory of arousal informed by LC/NE function (Li et al., 2023; Nassar, 2024), our results

may reflect dynamics of any or all of these neuromodulatory systems. ACh and 5HT have also been linked to adjustments of learning rate (Yu & Dayan, 2005; Kurtenbach et al., 2024; Grossman et al., 2022), perhaps elevating their consideration as the possible neural basis for the latent state transition like signals that we measured. It is worth noting, however, that relationships between ACh and pupil diameter typically occur over a longer timescale than the transient dilations that serve as the basis of our conclusions here (Reimer et al., 2016), and that rodent optogenetic studies suggest contributions of serotonin to pupil fluctuations are smaller than those of LC/NE (Maheu et al., 2025). Thus, while it is possible that any of these systems could underlie our observed effects, their timing and magnitude, coupled with their agreement with an existing theory of LC, make norepinephrine a candidate neuromodulatory system of particular interest."

The Methods seems to be missing a section on Subjects.

We apologize for this oversight and have now included more information about our participants (page 30):

"We collected behavioral, EEG, and pupillometry data from 63 healthy adult participants (33 female; mean age = 24.43 years, SD = 5.58, range: 18–40 years)."

Minor:
- The text often cycles back and forth between the paste and present tense.

Apologies, we have gone through carefully to try to fix the tense issues that the reviewer refers to.

- Figure panels are often not referenced in order. For example, in the main text, "Figure 2F&G" is referenced before "Figure 2D-E"; note that the authors also use different formatting here (& vs. -).

The formatting issue has been fixed. For some of these figures, we reference panels out of order as their arrangements would not be ideal had they been arranged in the order they were discussed in the manuscript. In the example referenced here, we discuss the mean EEG signals before discussing the surprise signals. However, we wanted to keep the channel ERP traces together at the bottom of the figure rather than separating them.

- "Thus, participants biased their reproductions toward their predictions and reduced this bias in response to surprising stimuli reflecting likely state transitions" (line 288). It would be nice if the authors can test this idea: directly compare bias on trials just before and just after state transitions?

Our manuscript previously tested this idea through a behavioral regression, which indicated that prediction errors were less influential on perceptual errors for trials when the state transition probability was high (negative PE*STP interaction coefficients; see Figure 3D in manuscript). However, we agree that the analysis suggested by the reviewer provides another way to get at the same idea, and we've implemented it below. When comparing the average raw bias for a stimulus just before a surprising trial (t-1) with the average raw bias just after a surprising trial (t+1), we find that bias is significantly lower after the surprise trial (bias before surprise mean/SEM = 0.342/0.008; bias after surprise mean/SEM = 0.313/0.010; $t(62) = 3.32$; $p = 0.0015$). The plot below shows each subject's average bias before all surprise trials plotted against their average bias after all surprise trials.

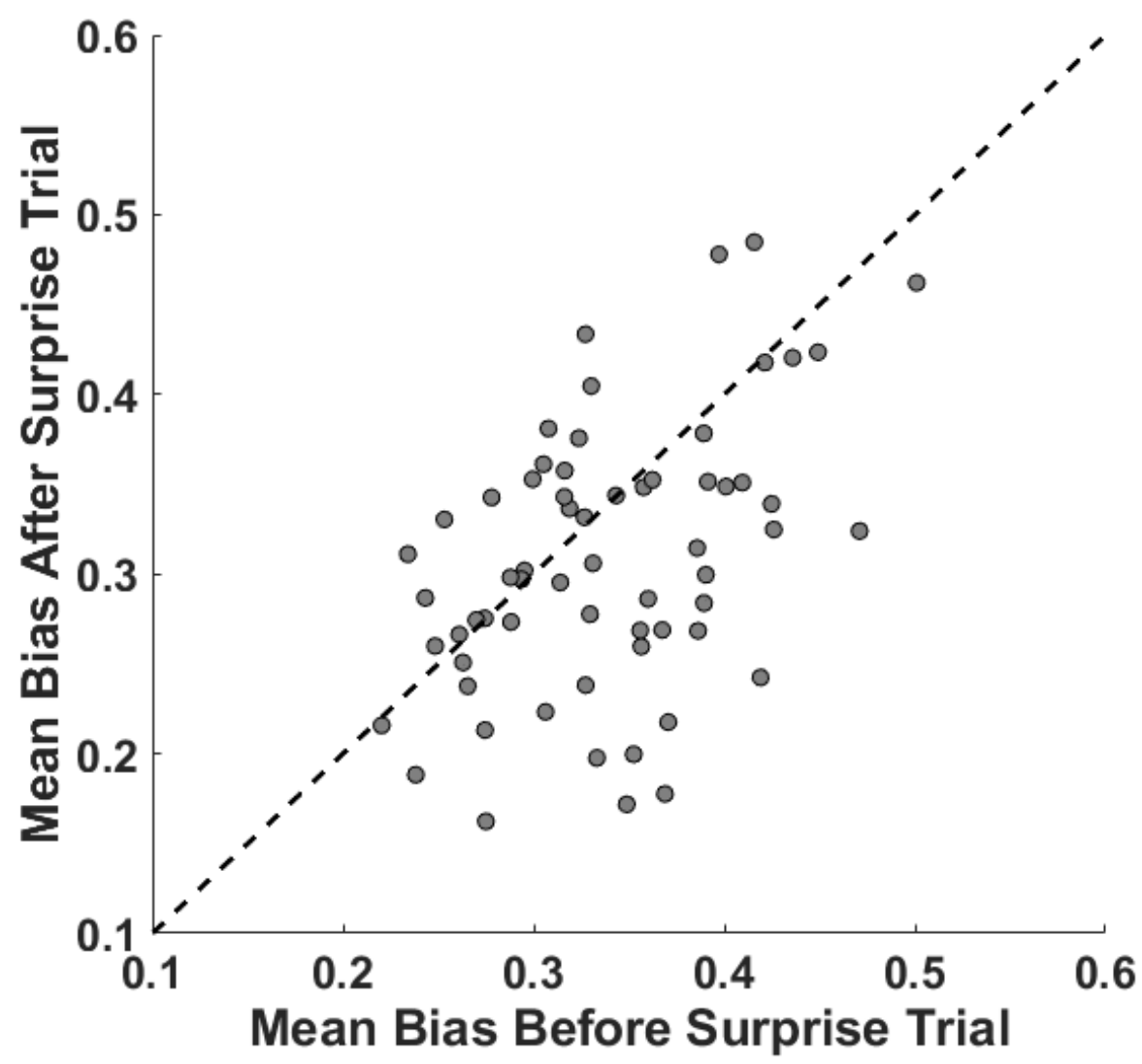

- What do the results in Fig. 3F-G look like for the average absolute bias (across the two stimuli), computed as the perceptual error divided by the objective prediction error (as in Fig. 3A)? If this is different than what is presented, what would this mean?

We understand the reviewer's question here to be highly related to the question above about using "max bias". As we noted in our response about that possible measure, our "mean bias" measure was selected based on its relationship to state transition probability in synthetic data generated from our Bayesian model. While there are other ways to quantify bias, we found that the mean bias measure reported in the paper provided the best correlation to underlying state transitions in synthetic data from our Bayesian model. The advantage of our method is that it includes data from both stimuli in a single regression model, enabling coefficients to depend more heavily on the stimulus with a greater prediction error, and therefore more leverage in the regression. This is important since some trials include prediction errors near zero – and computing bias according to a single stimulus (by dividing the perceptual error by the prediction error for that stimulus) – will

yield very extreme bias measurements in these cases. We attempted to minimize the impact of these small prediction error trials by constraining bias measures between 0 and 1, however in practice, this led to a very large number of bias measures at exactly 0 and 1. In synthetic data from our Bayesian model, mean absolute bias measures computed in this way did correlate with latent state transitions (r = -0.40), as did maximum bias measures (r = -0.24) but such correlations were much weaker than those observed with bias levels inferred using the method in our paper (r = -0.68). Indeed, we selected our method for measuring bias based on simulations with the Bayesian model in order to maximize its negative correlation with latent state transitions, reasoning that a method that works best to capture known relationships in our model would serve as the best tool to test for those relationships in the human data.

Consistent with this, when we used the mean absolute bias measure proposed by the reviewer, which is less reliably correlated with latent state transitions, we see weaker versions of the same relationships reported in our paper (See figure below). We interpret this as indicating that the primary effects we report are real, and evident using other bias measures, but that the magnitude of the effects observed will depend on the signal-to-noise characteristics of the bias measure. For this reason we have retained our original methods in the paper, which were optimized for their ability to identify signals of interest in synthetic data.

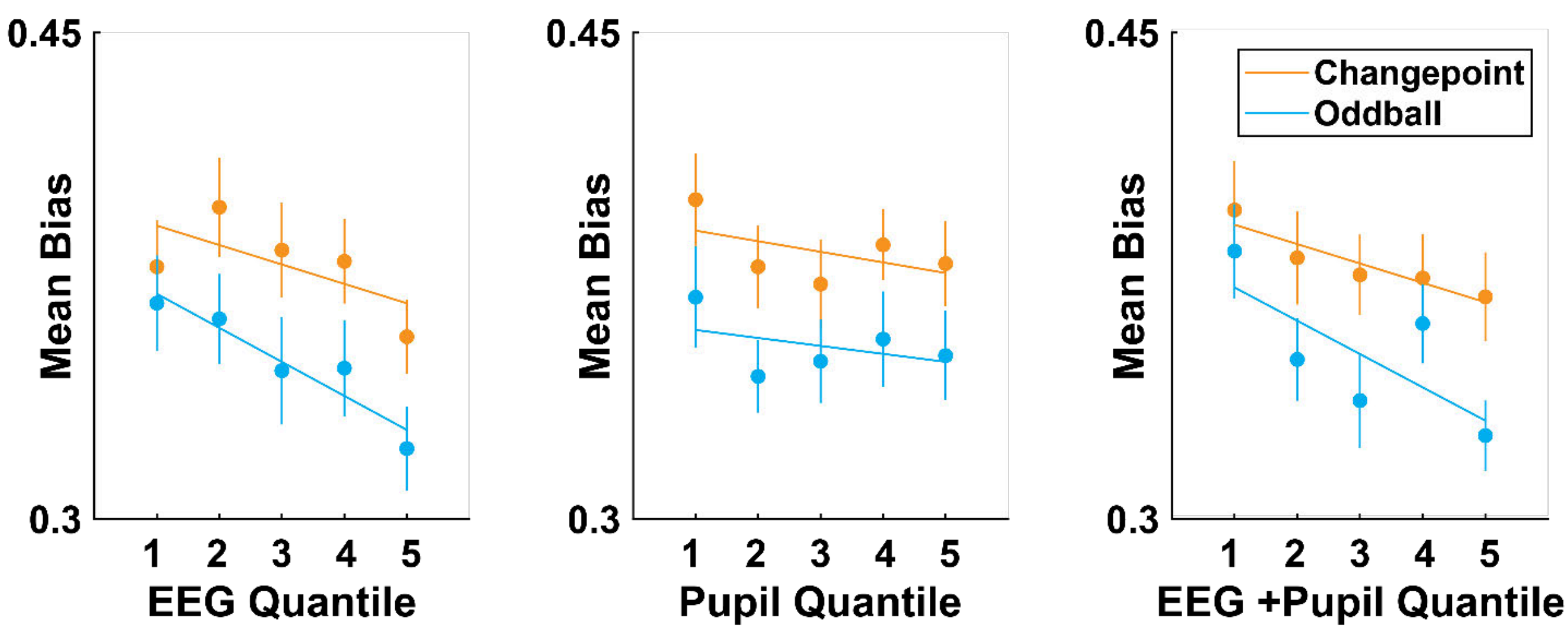

REVIEWER 2

In the present study, the authors tested five predictions made by a model of arousal's role in producing latent state transitions. Overall, the predictions were supported by their data. The data came from a color prediction and reproduction task. On each trial, participants were asked to make a prediction about one of two upcoming colored items. Then they would see two colored items, then reproduce the color of one of the two items after a delay. In both critical blocks, the colors of the items were drawn either from a von Mises distribution. Further, trials were occasionally drawn from a uniform distribution. In the changepoint environment, these abrupt discrepant trials signaled a permanent change to a new center point of the von Mises distribution. In the oddball blocks, the discrepant trials lasted only one trial. Arousal signals were drawn from both EEG event-related potentials and pupillary dilations.

Overall, my evaluation of the manuscript was positive. I thought the task had an interesting design with which to ask some interesting questions. I have a few questions I would like the authors to consider.

We thank the reviewer for the positive evaluation of our work and have addressed all concerns below point-by-point.

1)The pupil responds transiently in many different environments, and in addition to being a signal of arousal, it has been used as a signal of cognitive effort, even tasks where there is no "surprise" associated with the task. I was curious about a few things. First, what did the pupillary (and perhaps EEG signals) look like on non-discrepant trials? Do those trials provide any information about the attentional state people were in? Does that have any bearing on the latent state transition theory?

We completely agree with the reviewer that effortful cognitive actions elicit pupil dilations even in the absence of surprising external stimuli. In some sense, our finding that oddball events are preceded by a delayed pupil dilation that exceeds that observed in changepoints might be considered to fall in this category – since oddballs and changepoints are matched in terms of surprise, but the former requires an additional, potentially effortful cognitive action, to retrieve the latent state relevant to the upcoming prediction. We have now made this link explicit in our discussion (pages 22-23).

"Another account of arousal that relates to our results is that of effort (Kahneman & Beatty, 1966; Varazzani et al., 2015; Bornert & Bouret, 2021). One could argue that more effort might be required for surprising trials that reflect latent state transitions than for more typical

ones. This explanation could also extend to the pupil dilations observed during the second latent state transition predicted to occur on oddball trials since these trials have an additional memory component absent on changepoint trials. More generally, if transitioning internal representations of latent state is an effortful process, then signatures of effort should always accompany them, making these two constructs difficult to tease apart with correlative methods. Here we show that the impacts of arousal on behavior can be well predicted by a latent state account both within and across participants, but future work manipulating arousal directly could be useful in untangling the causal structure of the relationships between arousal, latent state transitions, and effort."

It is noteworthy though, that these pupil differences were delayed and only observed when aligning pupil traces to the subsequent prediction, rather than the stimulus onset.

Regarding the more specific suggestions, we have plotted the mean pupil response to stimulus onset for normal trials and surprise trials separately for both blocks below. Changepoint and oddball trials indicated are trials with greater than 0.2 average state transition probability. Participants respond similarly to normal trials in both blocks, but the dilation magnitude is slightly higher in the changepoint block. We also see that surprise trials induce similar dilations in both environments. Changepoints induce dilations more quickly that tend to last a little longer, while oddballs induce slightly slower shorter lasting dilations. We used the regression approach in our manuscript since it is more sensitive to the continuous scale of surprise in the task and can consider other influences (e.g. condition, entropy, presented color) simultaneously. However, if the reviewer feels that there is information in the direct comparison of neutral and high STP trials that could be useful in the manuscript we would be more than happy to incorporate these figures.

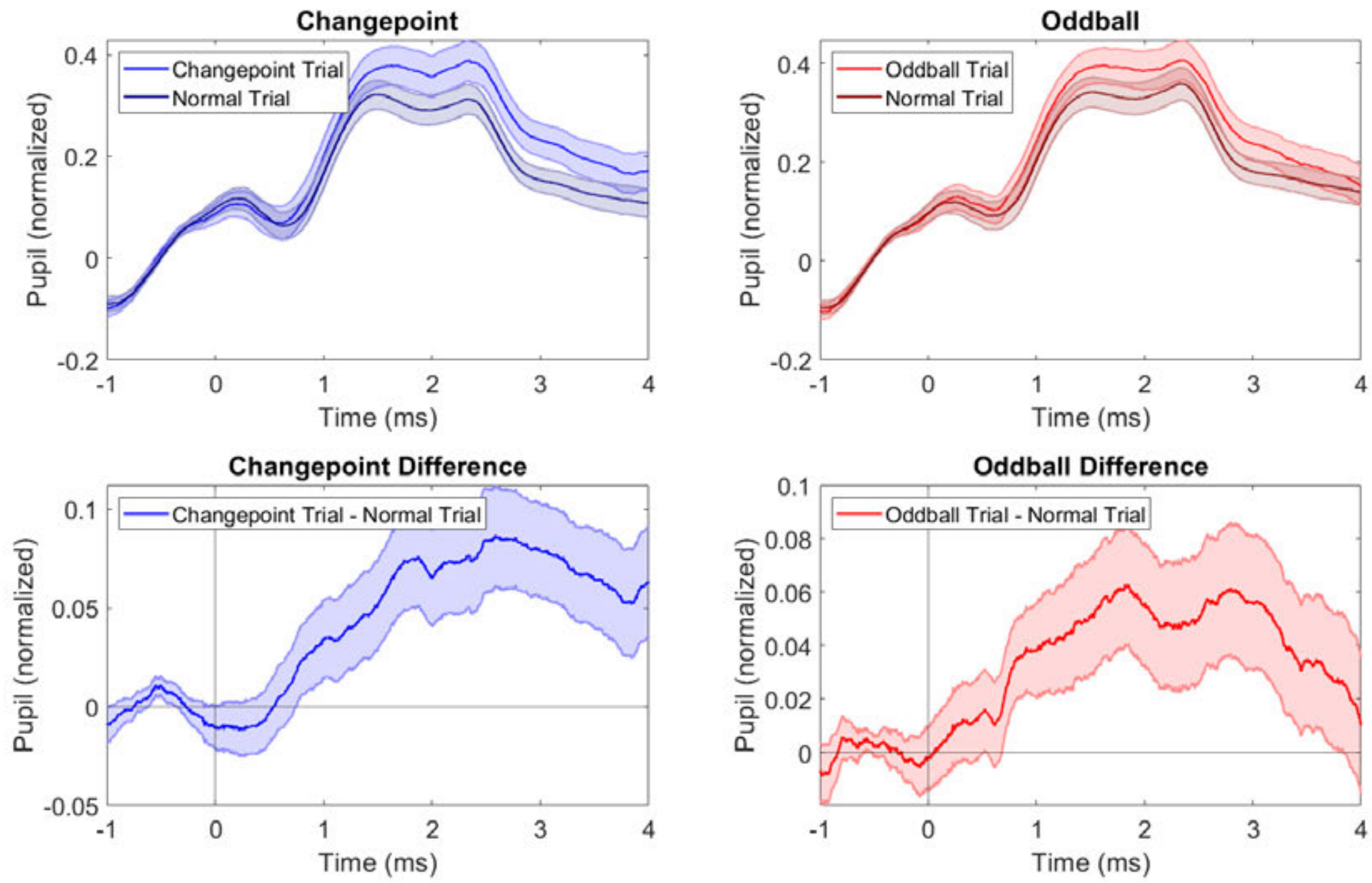

2) What are some new and untested predictions of the theory that might generate future work? There are a few mentioned in passing in the Discussion, but I think this addition would go a long way in increasing the theoretical advancement of the paper.

One important prediction of our theory is that the relationship between arousal and learning behavior should depend critically on subjective expectations about how states will transition. In particular, if an individual expects an outlying event to reflect a changepoint, then stimulus-locked arousal responses in that individual should positively predict learning, whereas if an individual expects an outlying event to reflect an oddball, then their arousal responses should negatively predict learning. The new analysis performed in response to concerns raised by reviewer one provides a limited test of this idea and supports it.

However, the basic idea has much broader implications which could and should be tested. If supported, such tests might explain the considerable variability in the literature with respect to when and how arousal signals relate to learning behavior (Nassar et al., 2012, O'Reilly et al., 2013, Jepma et al., 2016). One directly testable prediction from our study is that, in absence of explicit knowledge about the generative environment, relationships between arousal and learning should emerge and evolve over time as people learn the latent state transition structure. In terms of neural mechanisms, our theory predicts that two individuals who differ in their relationships between arousal and behavior likely do so because they are assuming different transition structures – an idea that would be testable

in studies leveraging fMRI to directly measure how latent states transition in response to surprising feedback (Vaidya & Badre, 2022, Nassar et al., 2019, Muller et al., 2019, Schuck et al., 2016). We have now added an additional paragraph to our discussion highlighting these predictions and their implications (page 22).

"These results, coupled with our motivating theory, provide a potential explanation for some of the heterogeneity in the broader literature linking arousal to measures of learning (Nassar et al., 2012; O'Reilly et al., 2013; Jepma et al., 2016). In particular, the prediction of our model, which is consistent with the individual differences in relationships observed in our study, is that two tasks or individuals that differ in their structural assumptions about the environment, could and should have very different relationships between their arousal level and subsequent learning. This prediction could be tested explicitly in future studies that probe structural understanding directly, measure arousal relationships as structure is learned de novo, or that rely on neuroimaging to identify proxies of current and anticipated future latent states (Schuck et al., 2016; Nassar et al., 2019b; Vaidya & Badre, 2022)."

3) Perhaps I missed this info, but why did the task have two colored items instead of just one? Was that an important feature of the task? Along those lines, I assume the colors of the items on the two sides of the screen were generated from different distributions. Is that true? If so, doesn't that mean that people are continually updating two models about the colors? Does that fundamentally change the nature of the task, compared to other paradigms where only one source of information is being used?

Our decision to include two colored items per trial was primarily to increase the difficulty of the visual working memory component of the task. Our bias predictions come from the idea that the brain would perform a Bayesian (precision weighted) combination of two sources of information: 1) sensory/memory content regarding the color observed and 2) prior information about the color that was expected. When the precision associated with sensory reports is high, as it was when we piloted a version of a task with only a single memorandum, our Bayesian model predicted that participants would rely almost completely on sensory information and should thus be only very minimally biased towards their predictions. This design would be problematic since our theory predicted a reduction in bias from baseline at state transitions and during periods of transient arousal. To increase our power to see such a reduction, we added a second stimulus, which enabled reduced the precision of color reports and consequently increased the optimal levels of bias. Having a second stimulus also had a side benefit that it increased the number of changepoint/oddball events in our paradigm, allowing us to collect more of the measurements critical to test our theory within a fixed number of trials. We now report more information about our decision to use two stimuli in our methods (pages 26-27).

"The advantage of presenting two simultaneous stimuli was two-fold: first, it allowed us to increase the number of surprising events that would be included in an experimental session, and second it led to reproductions that, while related to the presented color, were somewhat imprecise, meaning that they could be improved through combination of sensory information with prior expectations. Stimuli were prompted in a randomized order."

4) The axis labeling in Figure 2 was odd. There are no units mentioned for many of the y axes. I also don't know what is meant by "intercept" on the y-axes of Figures 2B, 2F, and 2G.

We apologize for any confusion this caused. "Intercept" in these figures refers to the average intercept term in the running pupil and EEG regressions. The units for these figures are microvolts for the EEG regression's intercept term, microvolts per standardized unit of STP for the STP term, and pupil size z-units for the pupil regressions. Ticks and tick labels have been added for readability in these figures in the revised version of the manuscript, and units have been added to the figure captions.

5) Where did the authors file their preregistration? Perhaps I missed that too but I couldn't find a link to that.

Our preregistration was filed on OSF: (osf.io/2wcxt). We have now included a link to it in our manuscript, both in the main text and in the references.

6) There are times in the manuscript when the citing is awkward. For example, P. 20 when it's stated, "…the 'network reset hypothesis' was proposed by Bouret and Sara in 2005 (Bouret & Sara, 2005). That is the case for the sentence regarding Yu and Dayan (2005) too.

Fixed.

7) When the authors say they used interpolation for the eye data, what kind did they use? Linear? Cubic spline?

We apologize for not specifying this previously. Linear interpolation was used. This has now been specified in the methods section.

8) It's probably sufficient to say p < .001 instead of using scientific notation for p-values. Using dof for degrees of freedom seemed odd to instead of the traditional notation of t(df) = t-value, p-value, effect size.

We have changed our notation for t values and degrees of freedom as requested and added effect sizes. We opted to not change our p-values for the sake of specificity and replicability of our analyses.

9) Was there a specific reason for doing the per-participant regressions rather than a simultaneous analysis like a linear mixed effects model?

We performed regressions separately for each participant because it enabled a streamlined approach for multiple comparisons correction: we could sign-flip subject level coefficients for all timepoints. This multiple comparisons approach would not work for a mixed effects model, since subject level coefficients in such a model are not independent of one another.

However, to verify that our results are not overly conditioned on our analysis choices, we have now performed a running linear mixed effects model on the two EEG channels that we plotted in our initial manuscripts figures 2F-I and these results are pictured below (see following page; top and middle left). The results for the intercept and STP terms in these models are comparable to the results we found using our individual subject regressions (top and middle right). We also performed a running linear mixed effects model on the pupil data similar to how it is plotted in figures 2B-C. The results for this model (bottom left) were also similar to the results we found using our previous pupil model (bottom right).

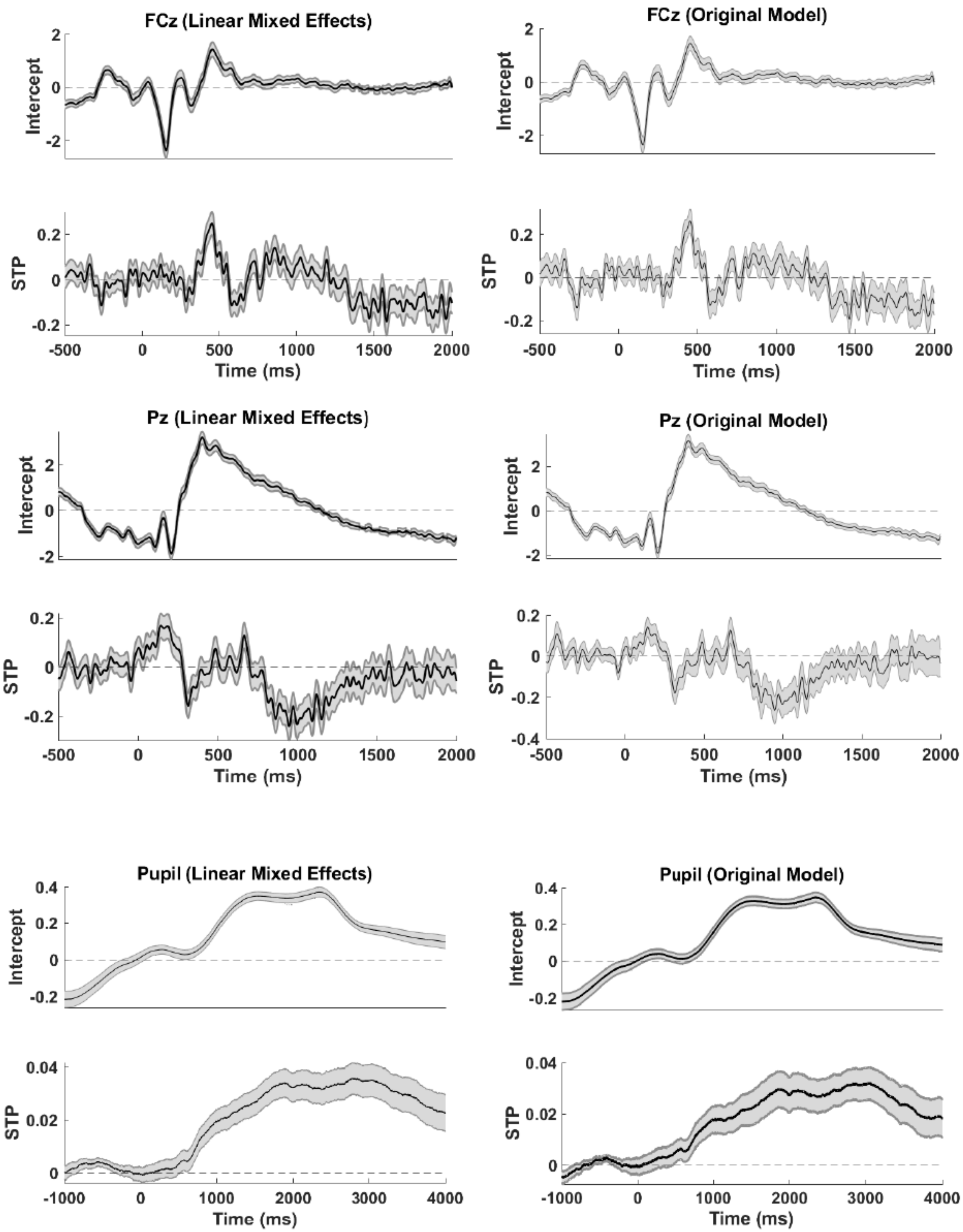
FCz (Linear Mixed Effects)
FCz (Original Model)
Pz (Linear Mixed Effects)
Pz (Original Model)
Pupil (Linear Mixed Effects)
Pupil (Original Model)
Intercept
STP
Time (ms)

REVIEWER 3

Li and colleagues study how fluctuations in arousal, as measured by changes in pupil size and the P3a ERP component, associate with a reduction in bias during a color reproduction and color prediction task. Overall the task the authors use is interesting and the question that they address is relevant. The paper is generally well-written, the analyses are overall solid, the figures are good and results interesting. The pre-registration is truly appreciated and a strong aspect of the paper.

We thank the reviewer for the positive evaluation of our work and have addressed all concerns below point-by-point.

I do have some suggestions for improvements and concerns that need to be addressed.

1. Theoretical background: The link between pupil size/P3a and the LC noradrenaline system is too simplisticly presented. Previous (mostly animal) work has shown that pupil size is probably driven by many neuromodulators including dopamine, noradrenaline, serotonin, orexin etc. The paper would benefit from a more nuanced interpretation of the data and more accurate link to theories of neuromodulatory function.

We completely agree with the reviewer that this is an important aspect to note when interpreting the results from our pupil data. We have since included a paragraph in the discussion (page 23) of the multiple neural correlates and causal contributors to pupil dilation and how it relates to the project. We have reproduced this paragraph below for ease of review:

"However, these proxies are inherently nonspecific. For example, pupil dilation in our task also likely corresponds to activation of multiple neuromodulatory systems—including acetylcholine (ACh), serotonin (5HT), and orexin—which each play distinct roles in modulating brain state and behavior (Cazettes et al., 2020; Grujic et al., 2023, 2024; Joshi & Gold, 2021 ; Maheu et al., 2025; Reimer et al., 2016). Thus, while our study was motivated to test a theory of arousal informed by LC/NE function (Li et al., 2023; Nassar, 2024), our results may reflect dynamics of any or all of these neuromodulatory systems. ACh and 5HT have also been linked to adjustments of learning rate (Yu & Dayan, 2005; Kurtenbach et al., 2024; Grossman et al., 2022), perhaps elevating their consideration as the possible neural basis for the latent state transition like signals that we measured. It is worth noting, however, that relationships between ACh and pupil diameter typically occur over a longer timescale than the transient dilations that serve as the basis of our conclusions here (Reimer et al., 2016), and that rodent optogenetic studies suggest contributions of serotonin to pupil fluctuations are smaller than those of LC/NE (Maheu et al., 2025). Thus, while it is possible that any of

these systems could underlie our observed effects, their timing and magnitude, coupled with their agreement with an existing theory of LC, make norepinephrine a candidate neuromodulatory system of particular interest."

2. Theoretical background/novelty: Overall the paper could benefit from a better embedding in the literature. The link between the pupil/P3 and surprise, prediction errors and learning has been studied for a long time (including modelling) and the authors focus only on a very selective part of that. Especially in experimental psychology many relevant studies have been performed, which should be incorporated, at least to a certain extent. The paper is also framed in a way that the novelty of the results is slightly exaggerated giving a large body of work out there, even when considering that writing for journal such as Nature human behavior may limit the number of references that one may use. Also considering that the reduction in decision bias with increased pupil size has been established for a few years now (see refs below, also cited by the authors), I think the authors can do a better job of highlighting the exact novel aspects of their study. Obviously, the specific experiment that the authors do has not been performed and the direct comparison between the two conditions (changepoint and oddball) is still relevant and interesting, but it needs better embedding and understanding (see also points 4-5).

We agree that our previous submission did not highlight the specific novel empirical contributions as clearly as it could have. The goal of our study was to understand the mechanisms through which arousal systems influence behavior. The key conceptual advances of our study were to 1) propose a latent state transition signal that should theoretically lead to adjustments in both learning and bias, and 2) demonstrate that EEG- and pupil-derived markers of arousal confirm five preregistered predictions for such a signal, and 3) thereby provide a normative unitary explanation for two prominent lines of work linking arousal to behavior. Thus, our work conducted a number of tests of a theory with potential to explain two well established lines of prior literature, one rooted in learning, and another in perception and decision making, through a single overarching theory.

Because of this focus, we framed our previous manuscript in terms of the theory that we tested. However, we now recognize that this framing failed to highlight which of our specific preregistered predictions were conceptually new – which seems to be the primary concern of the reviewer. Indeed, our first prediction, that both P300 and pupil diameter would increase at latent state transitions, reflects a conceptual replication of a large body of work – in line with the reviewer concern. However, each of the subsequent four predictions included testing of a novel hypothesis qualitatively distinct from any previous study. Two of these predictions included multiple parts, and in each case one of the tests corresponded

to a conceptual replication of previous work (pupil dilation corresponding to reduced perceptual bias; P300 corresponding to bidirectional learning rate adjustments) and the other part to a novel test (P300 corresponding to reduced perceptual bias; pupil dilation corresponding to bidirectional learning rate adjustments). The fourth prediction tested is completely unique to latent state transition theory, and the fifth prediction tested the core idea that bias and learning rate adjustments emerge through a common arousal-related mechanism. Taken together, the results from these tests provide strong evidence that state transition signals measured through EEG and pupil diameter jointly predict downstream behavioral consequences of latent state transitions including decreased bias a and bidirectional modulation of learning. We now clarify these points in an introductory paragraph of our revised manuscript (page 4), which we include below.

"While the latent state transition theory (LSTT) accounts for a wealth of data linking arousal signals to surprise (Alamia et al., 2019; Fouragnan et al., 2017; Kloosterman et al., 2015; Kopp & Lange, 2013; Liao et al., 2018; Mars et al., 2008), prediction errors (Bianco et al., 2020; Braem et al., 2015; Kamp & Donchin, 2015; Raisig et al., 2010; Zhao et al., 2019), and behaviors including learning (Colizoli et al., 2018; Filipowicz et al., 2020; Ghosh et al., 2021; Harris et al., 2022; Jepma et al., 2016; Nassar et al., 2012) and perceptual biases (de Gee et al., 2014, 2017, 2020; Krishnamurthy et al., 2017; Leong et al., 2021; Urai et al., 2017), it also makes a number of novel predictions that have yet to be tested. Related to learning, LSTT was developed to explain why EEG-derived signatures of arousal, such as the P300, correspond to increased learning following changepoint events but decreased learning following oddball events (Nassar et al., 2019a). But it further predicts that these bidirectional learning relationships should also hold for other peripheral measures of arousal such as pupil dilation, which has yet to be tested. Related to bias, LSTT accounts for findings that pupil dilations correspond to reduced perceptual bias in changing environments (de Gee et al., 2020; Krishnamurthy et al., 2017b; Urai et al., 2017). But it further predicts that this relationship should hold across different statistical environments (changepoints/oddballs) and across different signals that are sensitive to transient changes in arousal, such as the P300. Both of these predictions are yet to be tested. Finally, if arousal impacts both learning and bias by energizing latent state transitions, then individuals who carve time into discrete latent states should show stronger relationships between arousal and both behavioral measures, supporting a single arousal construct. Here we develop an empirical framework that allows us to test these and other key predictions of LSTT, using EEG and pupillometry-based markers for arousal as indirect proxies for LC/NE activity."

3.Methods: Regarding the EEG, I'm a bit puzzled by the large baseline differences before stimulus presentation in Fig 2 and worry about data quality, which may be due to the very low trial numbers per block for EEG, but also for pupil analyses (120, meaning there are only a few changepoints). Please specify how many changepoints there were in each condition. Do the authors have an idea why the baseline differences are so large for Pz? The head maps also look fairly noisy given the sample size.

We believe that the reviewer is referring to figure 2G of our manuscript, which shows the intercept coefficient from a regression model applied to EEG data recorded from the Pz channel. The reviewer's concern is that data on the Pz electrode implies a difference in signal amplitude during the baseline period, before the stimulus was actually observed. However, there seems to be a slight misunderstanding here, as that is not what we observed. We acknowledge that this misunderstanding is likely due in part to the lack of clarity in our previous manuscript regarding the normalization in our regression models – which we have now revised (see response to reviewer 2 and new text in methods; page 34). However, below we explain our methods in detail, why deviations from zero in the intercept term should be expected to occur even before stimulus onset, and that we do not see any evidence for stimulus-related changes in EEG signaling occurring before stimulus onset.

Our manuscript presented regression coefficients reflecting the intercept and state transition probability (STP) coefficients from a regression model that was applied to each channel and timepoint. STP reflects the difference between state transition trials (changepoints/oddballs) and neutral ones – and thus seeing non-zero coefficients for this term before participants observed an outcome would suggest, as the reviewer says, a major problem. But instead, we observed STP coefficients that are indistinguishable from zero throughout the baseline period, with changes from zero only occurring after >200 ms and not reaching statistical significance until considerably later (Figure 2H-I). Thus, the aspect of the data that concerned the reviewer with respect to our methods is not actually present.

We believe that the confusion arose from misinterpretation of the intercept coefficients, which were plotted above the STP coefficients (Figure 2F-G). The intercept coefficients are intended to capture the mean of the EEG signal at each time relative to stimulus presentation. As such, they can and will deviate from zero, even during the baseline period, which would imply that the baseline measurement window has a mean EEG signal amplitude that differs from the grand mean. We believe that the reviewer, perhaps thought

that the intercept coefficient that was plotted reflected a baseline subtracted ERP. Baseline subtraction should indeed force the signal to be zero during the baseline period, however such an approach is not statistically advisable, since baseline subtraction can add noise associated with the baseline measurement to the ERP (see here for explanation and simulations: https://datacolada.org/39). Our regression approach allows us to include mean centered baseline period measurements as a nuisance regressor such that we can remove shared variance from our signal coupled to the baseline without adding any additional noise associated with the baseline measurements. In short, non-zero coefficients are not an indication of a problem, but instead highlight the statistical rigor of our methods, which go beyond those typically applied in the field.

4. Methods: Because the subjects are fully instructed about the task design of the two different blocks it's clear for them that samples that are out of the ordinary (deviant ones) are task relevant in the change point condition and task irrelevant in the oddball condition. In the oddball condition these large deviations can be ignored. Therefore, when a task relevant change is detected (changepoint condition), and strongly associated with arousal changes due to that, this has a large impact on behavior (reduction in bias), whereas the opposite is true for the oddball condition (you should not attend or take notice of those changes at all in fact, and if you do this is detrimental for behavior). Based on this, can all the results be explained by differential attentional modulations of the pupil/P3a to task relevant versus task irrelevant stimuli? (see also point 5 below). Please discuss, I have added some relevant references below.

While the reviewer is correct that subjects in this study were fully instructed regarding the generative structure of colors in each condition, it is **not** true that participants could ignore oddball stimuli. In terms of the visual short term memory component of the task, participants are still required to memorize and report the color of oddballs, and doing so effectively requires minimizing the level of bias in those reports, much like for changepoints (see figures 3B and 3D in previous manuscript). Thus, for color reproductions, oddballs and changepoints place similar demands on attentional systems – stimuli must be encoded, and ideally recognized as deviated from expectations, so as to allow them to be interpreted with minimal bias towards those expectations.

With respect to making an upcoming prediction (learning), we agree that, in principle, participants could have achieved normative behavior by increasing attention to changepoints and ignoring oddball events. However, this explanation is inconsistent with our results. The reviewer asks whether "all the results be explained by differential attentional modulations of the pupil/P3a to task relevant versus task irrelevant stimuli?" An explanation like this would be possible if arousal signals (P3a, Pupil dilations) were

increased at changepoints, consistent with attentional upregulation, and decreased at oddballs, consistent with attentional downregulation. We note that such a strategy would be problematic in that it would likely have consequences for the color reproductions for oddball events. But more importantly, it is also **not what we observed**.

Instead, we see both P3a and pupil dilations increased at both changepoints and oddballs (see Figure 2C-D). A key advantage of our experimental design is that we can not only relate arousal signals to conditions of interest, but also to subsequent behavior – enabling us to make inferences about the mechanistic role of these signals. Doing so enabled us to see that arousal signals relate to perceptual bias similarly in changepoint and oddball conditions (see Figure 3F&H), but that they relate to opposite behavioral patterns in terms of learning, with heightened arousal linked to faster learning rates in the changepoint condition and slower learning rates in the oddball condition (see Figure 4F&H). These results mirror predictions of the latent state transition theory we tested but are inconsistent with the notion that they reflect task relevance of stimuli as suggested by the reviewer – as this idea would imply that that oddball events would have yielded smaller P3a and pupil dilations, and these signals would have been positively correlated with subsequent learning behavior – where in fact we see the opposite.

An alternative interpretation of the reviewer comment, which could be a bit more consistent with our data, is that increased attention is required for both changepoints and oddballs – in one case to focus attention on a data point that is important for upcoming predictions, and in the other to redirect attention in response to an oddball, first redirecting it to focus on the oddball color, so as to be able to accurately make a reproduction, but then away from it, so as to enable predictions that are unaffected by it. That said, it is not exactly clear from this view how the same attentional signal, if it is directed at the stimulus itself, could predict a more unbiased representation of an oddball stimulus to emerge (consistent with more attention to stimulus), but also predict less impact on future predictions (consistent with less attention to stimulus). Our latent states framework opens the door to another version of the attention story, whereby attention serves as a pointer to the active latent state representation rather than to the stimulus itself. In this view shifts in this pointer are accompanied by a P3a response. This idea would be completely consistent with our theory, since in this case a successful attentional shift at an oddball event would direct attention away from previously active representations, thereby reducing bias toward them. On the other hand, this attentional shift in response to the stimulus, if accompanied by a subsequent shift back to the previous latent state before the prediction phase, would prevent the oddball event from contributing at all to predictions. In short, there are many ways that one could envision attention being employed in our task, and while many of

these possibilities are inconsistent with our results, some of them are completely consistent and complementary to our results and overarching theory.

We have now added a discussion paragraph in which we explicitly lay out this logic and discuss how our results fit in with previous work regarding attentional modulation according to task relevance (page 25).

"The P3a has previously been observed in response to novel and surprising stimuli (Polich, 2007; Polich & Comerchero, 2003; Friedman et al., 2001; Escera et al., 2000) as well as related to internally mediated shifts in attention (Berti, 2008, 2015; Holig & Berti, 2010). These situations roughly map onto the ones requiring externally triggered and internally mediated latent state transitions in our task, respectively. Indeed, through this lens, the problem of latent state updating could be thought of as an attentional control problem, where latent state representations are selected through an attentional spotlight that is controlled according to internal and external factors. Our theory and data thus extend this line of work by showing how attentional control should be, and is, applied to dynamically adjust learning and perceptual bias in temporally structured environments. This theory provides mechanistic insights that differ qualitatively from common notions of relevance within the attentional field, since our theory predicts that even though oddballs could be ignored for purposes of learning behavior, they necessitate a latent state transition, which could be conceptualized a shift in attention to an alternate "oddball" latent state. P3a data from our task support this mechanistic view, since oddballs elicit larger P3a responses that in turn relate to reduced learning from oddball events."

Also, we are not quite sure why the reviewer's concern is related to the fully instructed nature of our task, but it is probably worth mentioning that we have collected data on an uninstructed version of our task for a follow-up EEG study and that the key ERP results described above generally hold in that study, with primary differences being the emergence of relationships over time. Thus, we do not think that our results are particularly tied to the fully instructed nature of our generative structures.

5. Interpretation: I think the authors should be a bit more upfront about the fact that the frontal P3a effects were unexpected and that the authors were expecting a parietal P3b effect (Fig 2) in the results section already (as well as abstract). Now the authors address this issue in the discussion section only. The discussion on this issue is also relatively superficial, citing very few studies. The P3a is often associated with attentional orienting and less so with surprise/novelty. Especially given point 4, it may be that the P3a mostly reflects attentional processes triggered by unexpected task-relevant vs irrelevant stimuli in such a perceptual task. At least some further discussion and better embedding in the literature is required here (even better would be analyses that tackle this issue). Also in the

abstract the authors refer to "the P300" and the informed reader will directly assume that this relates to the parietal P3b (given the known link to arousal modulations), only later to learn that it's about the P3a.

We apologize for the lack of specificity in this aspect of the discussion. Firstly, when we refer to the prediction that P300 would be related to behavior in the task, we were expecting both the P3a and P3b to be elevated on arousing trials, as those were the results we saw in a previous study that involved a predictive inference task with changepoint and oddball conditions (Nassar et al., 2019). So while it was unexpected that we did **not** see a parietal P3b effect, it was not unexpected that we **did** see a frontal P3a effect. In fact, there was also at least a hint from our previous paper that the frontal P3a might be playing a more functional role in the process than the P3b, since only the earliest P3a component was predictive of upcoming learning behavior beyond that which could be explained by computational modeling (see figure 5 from that paper).

To reiterate, the idea that "the P3a mostly reflects attentional processes triggered by unexpected task-relevant vs irrelevant stimuli" is not in agreement with our findings, since the P3a is elevated both in response to task relevant stimuli (changepoints) and task irrelevant stimuli (oddballs). Furthermore, the level of its elevation is predictive of upcoming behavior, both in terms of the degree to which reproductions are biased toward expectations, and in terms of the degree to which predictions are contextually updated in response to the most recent stimuli.

Nonetheless, we have now revised our manuscript so that 1) our abstract clarifies that our findings relate to the P3a , 2) our introduction specifies our predictions regarding both P3a and P3b (page 5) and 3) our discussion clarifies how our findings relate to previous work on modulation of P3a including the inconsistency of our results with the notion of attentional up or downregulation according to task relevance (page 25):

"*It is noteworthy that, while our preregistration did not distinguish between different subcomponents of the P300 response, we found that only the early P3a component related to latent state transitions and their theorized downstream effects on behavior, whereas the later P3b component was not reliably modulated by latent state transitions.*"

"The P3a has previously been observed in response to novel and surprising stimuli (Polich, 2007; Polich & Comerchero, 2003; Friedman et al., 2001; Escera et al., 2000) as well as related to internally mediated shifts in attention (Berti, 2008, 2015; Holig & Berti, 2010). These situations roughly map onto the ones requiring externally triggered and internally mediated latent state transitions in our task, respectively. Indeed, through this lens, the problem of latent state updating could be thought of as an attentional control problem,

where latent state representations are selected through an attentional spotlight that is controlled according to internal and external factors. Our theory and data thus extend this line of work by showing how attentional control should be, and is, applied to dynamically adjust learning and perceptual bias in temporally structured environments. This theory provides mechanistic insights that differ qualitatively from common notions of relevance within the attentional field, since our theory predicts that even though oddballs could be ignored for purposes of learning behavior, they necessitate a latent state transition, which could be conceptualized a shift in attention to an alternate "oddball" latent state. P3a data *from our task support this mechanistic view, since oddballs elicit larger P3a responses that in turn relate to reduced learning from oddball events."*

Minor

-I assume that the intercept plots in Fig 2F/G reflect averaged ERPs, and if so, this should be clarified (for the pupil in 2yB this becomes clear). The reader not familiar with the regression approach may not grasp this directly.

We apologize for any lack of clarity on our part. These intercept plots do represent average baseline-regressed ERPs. This has been clarified in the figure caption which now reads (page 11):

"(F-I) Lines/shading reflect Mean/SEM coefficients in millivolts for the baseline-regressed mean (intercept) (F/G) and STP (H/I) terms of the EEG regression for channels FCz (F/H) and Pz (G/I)."

- What is meant by usable data (line 860)? A better rationale for why some subjects were excluded is necessary.

We apologize for explaining our rationale for subject exclusion better in the previous version of our manuscript. The reasoning we used when analyzing data is now included in the manuscript as follows (pages 30-31):

"Two participants were unable to complete the task while EEG data was recorded, and one EEG dataset was not recorded due to researcher error. Participants with >50% rejected trials or >25% rejected channels were excluded from further analysis (n=3) … Subjects with more than 50% missing [pupil] data on 50% or more of the trials were rejected from eye data analysis entirely (n=3)"

- I often do not fully get the details of the methods or analysis (or only after re-reading parts here and there) and I think some of the methods/analyses require a bit more explanation in the results section for the reader to follow what's going on. In general, the write up of the methods and results could be a bit more intuitive and less technical. I would prefer to have more task description in the introduction/results to better follow the results section.

We now include more task description at the beginning of the results section in plain language to clarify our procedure.

- I think the authors should be slightly more cautious in the interpretation about ”optimization of perception”. What the authors show is that larger pupil/P3a responses are associated with a reduced bias, whether perception is optimized is whole different question.

We apologize for not being more explicit in what we meant by optimizing. We found that pupil/P3a responses were elevated on state transition trials at which predictions were unrelated to the stimuli that needed to be reproduced. We also found that trial-to-trial variability in pupil/P3a amplitude corresponded to reductions in perceptual bias. Since perceptual biases are typically useful in the task and allow participants to achieve better reproductions (See Figure S1A) and since our Bayesian model reduces bias at state transitions in order to optimize performance (See Figure 3D), we considered the specific reduction of bias on state transition trials, which was directly related to pupil/P3a amplitudes, to be a form of perceptual optimization. That said, we take the reviewer’s point that our definition of optimization is narrow and that our language on the issue could have been more precise. We have now changed the phrasing in several parts of the paper to say “reductions of perceptual bias” and where we discuss optimization, we are more specific about what we mean exactly.

References:

Jepma, M. *et al.* Catecholaminergic Regulation of Learning Rate in a Dynamic Environment. *PLOS Computational Biology* **12**, e1005171 (2016).

Muller, T. H., Mars, R. B., Behrens, T. E. & O’Reilly, J. X. Control of entropy in neural models of environmental state. *eLife* **8**, e39404 (2019).

Nassar, M. R., McGuire, J. T., Ritz, H. & Kable, J. W. Dissociable Forms of Uncertainty-Driven Representational Change Across the Human Brain. *J. Neurosci.* **39**, 1688–1698 (2019).

Nassar, M. R. *et al.* Rational regulation of learning dynamics by pupil-linked arousal systems. *Nat Neurosci* **15**, 1040–1046 (2012).

O’Reilly, J. X. *et al.* Dissociable effects of surprise and model update in parietal and anterior cingulate cortex. *Proceedings of the National Academy of Sciences* **110**, E3660–E3669 (2013).

Schuck, N. W., Cai, M. B., Wilson, R. C. & Niv, Y. Human Orbitofrontal Cortex Represents a Cognitive Map of State Space. *Neuron* **91**, 1402–1412 (2016).

Vaidya, A. R. & Badre, D. Abstract task representations for inference and control. *Trends in Cognitive Sciences* **26**, 484–498 (2022).
